# Longitudinal Relationships Across Bullying Victimization, Friendship and Social Support, and Internalizing Symptoms in Early-to-Middle Adolescence: A Developmental Cascades Investigation

**DOI:** 10.1007/s10964-024-02131-2

**Published:** 2025-01-18

**Authors:** Qiqi Cheng, Kathryn Mills-Webb, Jose Marquez, Neil Humphrey

**Affiliations:** Manchester Institute of Education, University of Manchester, Manchester, UK

**Keywords:** Mental health, Adolescence, Bullying, Friendship, Internalizing symptoms, Developmental cascades

## Abstract

Current understanding of the longitudinal relationships between different aspects of peer relationships and mental health problems in early- to mid-adolescence is limited. In particular, the role played by gender in these developmental cascades processes is unclear, little is known about within-person effects between bullying victimization and internalizing symptoms, and the theorized benefits of friendship and social support are largely untested. Addressing these important research gaps, this study tested a number of theory-driven hypotheses (e.g., interpersonal risk model, transactional model) regarding longitudinal relationships between bullying victimization, friendship and social support, and internalizing symptoms. The study sample was *N* = 26,458 adolescents (50.6% girls, average age 12 years 8 months (SD = 3.58 months) at baseline) attending *k* = 176 schools in Greater Manchester, England. Separating within-person effects from between-person effects, a random-intercept cross-lagged panel model (RI-CLPM) was applied to three annual waves of data. Analyses revealed that developmental cascade pathways varied across gender, as follows: higher rates of bullying victimization led to increased internalizing symptoms (partially for girls, fully for boys) and lower levels of friendship and social support (for girls only); higher levels of friendship and social support did not confer any protection against future bullying victimization (for girls or boys) but did lead to reduced internalizing symptoms (partially for girls, but not for boys); and, higher levels of internalizing symptoms led to increased rates of bullying victimization (for boys only) and lower levels of friendship and social support (partially for girls, fully for boys). Evidence of reciprocal relationships between bullying victimization and internalizing symptoms (for boys only) and between internalizing symptoms and friendship and social support (for girls only) was also found. Effect sizes of developmental cascade pathways varied but were mostly in the moderate-to-large range relative to the empirical distribution of cross-lagged effects in existing studies (i.e., 50th to 75th percentile). Sensitivity analyses indicated that findings were largely robust to a number of researcher-led analytic choices. The current study indicates that approaches to prevent or reduce the effects of bullying victimization should be prioritized, given the consistent evidence of its substantial role in increasing internalizing symptoms for both genders, in addition to its deleterious impact on girls’ friendship and social support. Preregistration: This study was preregistered at https://osf.io/xrwfq. The study design, hypotheses, and target analyses were registered.

## Introduction

Adolescence is characterized by the growing importance of peer relationships (Blakemore, 2018) and increased vulnerability for the development of mental health problems (Blakemore, 2019). Current understanding of the longitudinal relationships between different aspects of peer relationships and mental health problems is limited (Bernasco et al., 2022). More specifically, little is known about within-person effects between bullying victimization and internalizing symptoms (and vice versa) (Bartlett et al., 2023), and existing evidence is inconclusive about the direction of any associations (Sentse et al., 2017). The theoretically promotive benefits of friendship and social support in this context also remain underexplored (Bernasco et al., 2022). Finally, the potential moderating role of gender is currently poorly understood. Adopting a developmental cascades framework (Masten & Cicchetti, 2010) and using a statistical method that separates between- (i.e., stable differences between individuals) from within-person (i.e., situational changes within individuals) effects (Random Intercept Cross-Lagged Panel Model, RI-CLPM; Mulder & Hamaker, 2021), this study addresses these research gaps.

### Peer Relationships, Internalizing Symptoms, and Gender in Adolescence: Theoretical Perspectives

During adolescence, peer relationships become increasingly important as young people seek greater independence from adults and develop their identities as individuals (Blakemore, 2018). These relationships and interactions are transactional and reciprocal, and play a key role in development (Osher et al., 2020). Viewed through a developmental cascades lens (Masten & Cicchetti, 2010), the quality of peer relationships can influence, and be influenced by, other domains and functions, including mental health. With regard to the specific focus of the current study, three theoretical models have been proposed. First, the symptoms-driven model proposes that internalizing symptoms (e.g., feelings of worry, sadness and loneliness) precede relational difficulties with peers (Kochel et al., 2012). For example, individuals with elevated internalizing symptoms may be more likely to be withdrawn in social groups, which could elicit perceived negative responses from their peers, in turn leading to peer rejection or victimization (Beeson et al., 2020).

Second, the interpersonal risk model highlights the role of social relationships and experiences in the development of psychopathology, and posits that positive peer relationships promote wellbeing, while negative peer experiences such as bullying victimization will have social consequences for friendships, increase feelings of loneliness and lack of belonging, and precede and predict internalizing symptoms (Coyle et al., 2021). Finally, the transactional model acknowledges the dynamic interactions between an individual and their social environment (Sameroff & Mackenzie, 2003), recognizing the potential for reciprocal, direct and indirect influence of peer experiences on psychopathology and vice versa (Reijntjes et al., 2010).

There is reason to expect the nature and magnitude of these developmental cascade relationships may vary by gender (Panayiotou & Humphrey, 2018). Adolescence is a particularly salient period of gender intensification in which there are societal pressures to conform to normative gender expectations (Sravanti & Sagar Kommu, 2020). Girls and boys are socialized to think, feel, and behave differently in terms of emotional expression (Chaplin et al., 2005) and expectations and practices regarding friendship and social support (Van Droogenbroeck et al., 2018). The peer-socialization model (Rose & Rudolph, 2006) provides a useful integrative framework, proposing that exposure to same-sex peers contributes to the development of gendered peer relationship styles, stress and coping processes, and relationship provisions, which in turn are hypothesized to influence girls’ and boys’ mental health in different ways. For example, the effects of relational losses on the development of internalizing symptoms are proposed to be more salient for girls than for boys. This, it is theorized, is due to their greater emphasis placed on close dyadic friendships. When peer relations are perceived to be in jeopardy, this may lead to greater concerns over social approval and status, and in turn greater internalization of problems (Bakker et al., 2010; Ledwell & King, 2013). In sum, gender could be an important moderator of mental health, peer relationships, and the developmental associations between them (Van Droogenbroeck et al., 2018), meaning that empirical support for the three models noted above may be gender-specific. For example, the associations between peer relationships and internalizing symptoms may align more strongly with the interpersonal risk model for girls than for boys. In the subsections that follow, we review the available evidence, noting any such gender differences in developmental cascade effects.

### Bullying Victimization and Internalizing Symptoms

There is evidence of significant autoregressive paths (i.e., temporal stability: past values predicting current values) for both internalizing symptoms and experiences of bullying victimization at the within-person level (Fredrick et al., 2022; Siennick & Turanovic, 2023). Considering between-person associations between these constructs, the evidence base is inconclusive, finding mixed support for the symptoms-driven, interpersonal risk, and transactional theoretical models. For example, while some research has supported the transactional model, with reciprocal relationships between bullying victimization and internalizing symptoms (Boyes et al., 2014), other work has found partial support for different models depending on which aspect of internalizing symptoms (depression or anxiety) was measured (Bartlett et al., 2023; Sentse et al., 2017). For example, one study found evidence of reciprocal associations between bullying victimization and depressive symptoms over 12 months, supporting the transactional model. When considering social anxiety, they found that bullying victimization predicted anxiety over time but not vice versa, supporting the interpersonal risk model (Bartlett et al., 2023).

Another study found that depression predicted bullying victimization, supporting the symptoms-driven model (Sentse et al., 2017). These authors also found that while the relationship between anxiety and bullying victimization was reciprocal for girls, this was not the case for boys, where victimization predicted anxiety but not vice versa, supporting the interpersonal risk model. Time lag between measurements has varied in these studies (e.g., three times over one year vs every 12 months) and it is possible that the effects of bullying victimization on internalizing symptoms take longer to materialize. It is also worth noting that all these studies investigated these relationships in adolescents living in different countries and with different socio-economic and cultural backgrounds, and their divergent findings may be partially explained by these varying circumstances.

Building on this CLPM evidence, other research has investigated both between- and within-person relationships between bullying victimization and internalizing symptoms using RI-CLPM, finding reciprocal relationships at the between-person level, but when considering within-person associations, evidence for a symptoms-driven model among males, and a transactional, reciprocal model for females (Fredrick et al., 2022). Contrastingly, another study found reciprocal relationships between bullying victimization and internalizing symptoms, but did not test for gender differences (Siennick & Turanovic, 2023). This fact, and discrepancies between studies in terms of research design, such as length of time lags, age of participants, and analytical models used, mean that further research is warranted that examines the role of gender in the relationship between bullying victimization and internalizing symptoms in adolescence.

### The Promotive Benefits of Friendship and Social Support

While there is a growing evidence base using advanced statistical models to demonstrate both between- and within-person associations between bullying victimization and internalizing symptoms in adolescence (albeit with mixed results concerning the direction and reciprocity of associations), there is less research and agreement about the relationships between friendship and internalizing symptoms, and if social support confers benefits for young people who are the victims of bullying (Schacter et al., 2021). This is partly due to a predominance of cross-sectional and non-RI-CLPM longitudinal research (Schacter et al., 2021), with only a handful of studies investigating the direction and strength of within-person longitudinal relationships (with some exceptions; Murray et al., 2021; Siennick & Turanovic, 2023).

There is also heterogeneity in how friendship has been operationalized and measured, with different studies focused on different dimensions such as friendship quality, friendship quantity, and the role of best friends (Schacter et al., 2021). The current study focuses on friendship quality and social support, specifically relationships which are characterized by reciprocal positive affect and where an individual perceives these relationships to provide access to social resources. Theoretically, in both the interpersonal risk and transactional models outlined above, if a young person is the victim of bullying and perceives they have good friendships and access to social support, they may be more resilient to developing internalizing symptoms (Coyle et al., 2021). Conversely, if a young person does not feel that they have good friendships, they may feel they have less access to social support as a coping mechanism and be less resilient to the consequences of bullying. Lending partial support to the interpersonal risk hypothesis, one study found that positive perceptions of peer relationships had a protective within-person effect on the development of internalizing symptoms between ages 11 and 13, but not between ages 13 and 15. The authors found no evidence of a reciprocal relationship to support the symptoms-driven or transactional models (Murray et al., 2021). Another study reported slightly different findings, with no evidence of any within-person association between friendship support and internalizing symptoms among adolescents aged 11–14 years (Siennick & Turanovic, 2023).

Looking specifically at the within-person relationships between friendship and social support, bullying victimization and internalizing symptoms, there is currently limited empirical support for the potential promotive effects of friendship and social support. A recent study found that support from one friend had a concurrent buffering effect on the relationships between bullying victimization and internalizing symptoms between-persons, but this did not hold at the within-person level (Bernasco et al., 2022). When controlling for gender, there were associations between friendship support and internalizing symptoms in opposite directions for boys and girls. While these gender differences were not statistically significant, they could be indicative of different gender conceptions and societal expectations for boys and girls (Van Droogenbroeck et al., 2018).

Considering within-person longitudinal relationships, reciprocal negative relationships between friendship support and bullying victimization have been found, but no evidence that there was an indirect pathway from bullying victimization to internalizing symptoms via friendship support or vice versa (Siennick & Turanovic, 2023).

It is worth noting that baseline assessments for much of the literature reviewed above were carried out in the first decade of the 2000s. Since then, there have been huge technological advancements, increased globalization, a global recession, and a pandemic amongst other things. All of these events have changed the world in which adolescents are growing up and could have implications for development and wellbeing. Further, considering the studies which investigated the relationships between bullying victimization and internalizing symptoms, while several used more recent samples (Bartlett et al., 2023; Fredrick et al., 2022), data collection for all took place before the Covid-19 pandemic. In the UK, there is robust evidence to suggest that the proportion of adolescents with internalizing symptoms has increased since this time (Mansfield et al., 2022), and it is important to understand potential risk and protective factors to ensure prevention and intervention efforts are as effective as possible. Addressing these limitations, the current study draws on a large longitudinal dataset collected during the post-Covid-19 recovery period.

## Current Study

**Fig. 1:**
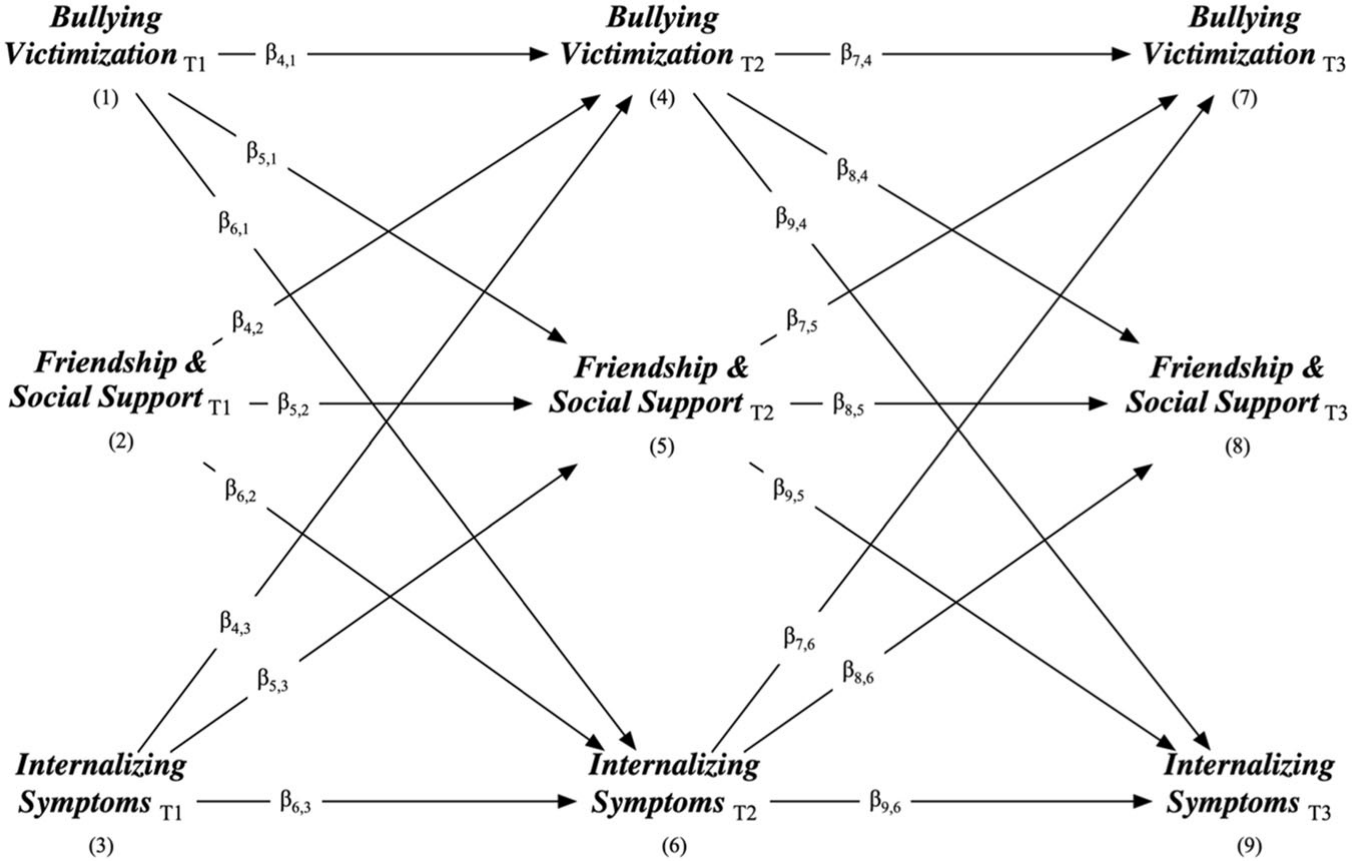
Conceptual diagram

Longitudinal relationships between peer relationships and mental health problems in early- to mid-adolescence remain poorly understood, particularly regarding the role of gender in these developmental processes and the potential benefits of friendship and social support. The current study addresses these gaps by examining within-person relationships between bullying victimization, friendship and social support, and internalizing symptoms. Prior to data analysis, the current study was pre-registered at https://osf.io/xrwfq. Several hypotheses were made. Bullying victimization was expected to have adverse consequences for later peer relationships and mental health (Hypothesis 1). Positive peer relationships were predicted to protect against later bullying victimization and internalizing symptoms (Hypothesis 2). Mental health difficulties were expected to precede relational difficulties with peers (Hypothesis 3). Both peer relationships and mental health were hypothesized to demonstrate within-person stability over time (Hypothesis 4). Peer relationships and mental health were expected to share a reciprocal relationship over time (Hypothesis 5). Finally, developmental cascades between peer relationships and mental health were predicted to vary by gender (Hypothesis 6). Table 1 delineates these hypotheses in more detail (e.g., theoretical and empirical support for each signposted, possible outcomes and interpretation) and Fig. 1 depicts the conceptual cross-lagged relationships across variables.

**Table 1:** Study hypotheses, possible outcomes, and interpretation

Hypothesis and underpinning theory/model		Prediction(s) (path directions in Fig. 1 where applicable)	Possible outcomes	Interpretation	Empirical support
H1: Bullying victimization has adverse consequences for later peer relationships and mental health. Interpersonal risk model (Coyle et al., 2021)	H1A: Bullying victimization negatively predicts later friendship and social support	${\beta }_{\mathrm{5,1}} < 0$, ${\beta }_{\mathrm{8,4}} < 0$	${\beta }_{\mathrm{5,1}} < 0$, ${\beta }_{\mathrm{8,4}} < 0$ ${\beta }_{\mathrm{5,1}} < 0,$ ${\beta }_{\mathrm{8,4}}\ge 0$ (or ${\beta }_{\mathrm{5,1}}\ge 0$, ${\beta }_{\mathrm{8,4}} < 0$) ${\beta }_{\mathrm{5,1}}\ge 0,$ ${\beta }_{\mathrm{8,4}}\ge 0$	H1A supported H1A partially supported H1A rejected	Siennick and Turanovic (2023)
	H1B: Bullying victimization positively predicts later Internalizing symptoms	${\beta }_{\mathrm{6,1}} > 0$, ${\beta }_{\mathrm{9,4}} > 0$	${\beta }_{\mathrm{6,1}} > 0$, ${\beta }_{\mathrm{9,4}} > 0$ ${\beta }_{\mathrm{6,1}} > 0$, ${\beta }_{\mathrm{9,4}}\le 0$ (or ${\beta }_{\mathrm{6,1}}\le 0$, ${\beta }_{\mathrm{9,4}} > 0$) ${\beta }_{\mathrm{6,1}}\le 0$, ${\beta }_{\mathrm{9,4}}\le 0$	H1B supported H1B partially supported H1B rejected	Sentse et al. (2017), Bartlett et al. (2023)
H2: Positive peer relationships confer protection against later bullying victimization and Internalizing symptoms. Interpersonal risk model (Coyle et al., 2021)	H2A: Friendship and social support negatively predicts later bullying victimization	${\beta }_{\mathrm{4,2}} < 0$, ${\beta }_{\mathrm{7,5}} < 0$	${\beta }_{\mathrm{4,2}} < 0$, ${\beta }_{\mathrm{7,5}} < 0$ ${\beta }_{\mathrm{4,2}} < 0$, ${\beta }_{\mathrm{7,5}}\ge 0$(or ${\beta }_{\mathrm{4,2}}\ge 0$, ${\beta }_{\mathrm{7,5}} < 0$) ${\beta }_{\mathrm{4,2}}\ge 0$, ${\beta }_{\mathrm{7,5}}\ge 0$	H2A supported H2A partially supported H2A rejected	Siennick and Turanovic (2023)
	H2B: Friendship and social support negatively predicts later Internalizing symptoms	${\beta }_{\mathrm{6,2}} < 0$, ${\beta }_{\mathrm{9,5}} < 0$	${\beta }_{\mathrm{6,2}} < 0$, ${\beta }_{\mathrm{9,5}} < 0$ ${\beta }_{\mathrm{6,2}} < 0$, ${\beta }_{\mathrm{9,5}}\ge 0$(or ${\beta }_{\mathrm{6,2}}\ge 0$, ${\beta }_{\mathrm{9,5}} < 0$) ${\beta }_{\mathrm{6,2}}\ge 0$, ${\beta }_{\mathrm{9,5}}\ge 0$	H2B supported H2B partially supported H2B rejected	Murray et al. (2021), Bernasco et al. (2022), Van Droogenbroeck et al. (2018)
H3: Mental health difficulties precede relational difficulties with peers. Symptoms-driven model (Kochel et al., 2012)	H3A: Internalizing symptoms positively predict later bullying victimization	${\beta }_{\mathrm{4,3}} > 0$, ${\beta }_{\mathrm{7,6}} > 0$	${\beta }_{\mathrm{4,3}} > 0$, ${\beta }_{\mathrm{7,6}} > 0$ ${\beta }_{\mathrm{4,3}} > 0$, ${\beta }_{\mathrm{7,6}}\le 0$ (or ${\beta }_{\mathrm{4,3}}\le 0$, ${\beta }_{\mathrm{7,6}} > 0$) ${\beta }_{\mathrm{4,3}}\le 0$, ${\beta }_{\mathrm{7,6}}\le 0$	H3A supported H3A partially supported H3A rejected	LoParo et al. (2023), Sentse et al. (2017)
	H3B: Internalizing symptoms negatively predict later friendships and social support	${\beta }_{\mathrm{5,3}} < 0$, ${\beta }_{\mathrm{8,6}} < 0$	${\beta }_{\mathrm{5,3}} < 0$, ${\beta }_{\mathrm{8,6}} < 0$ ${\beta }_{\mathrm{5,3}} < 0$, ${\beta }_{\mathrm{8,6}}\ge 0$ (or ${\beta }_{\mathrm{5,3}}\ge 0$, ${\beta }_{\mathrm{8,6}} < 0$) ${\beta }_{\mathrm{5,3}}\ge 0$, ${\beta }_{\mathrm{8,6}}\ge 0$	H3B supported H3B partially supported H3B rejected	Beeson et al. (2020)
H4: Peer relationships and mental health are stable within-persons over time. Developmental cascades model (Masten & Cicchetti, 2010)	H4A: Bullying victimization positively predicts later bullying victimization	${\beta }_{\mathrm{4,1}} > 0$, ${\beta }_{\mathrm{7,4}} > 0$	${\beta }_{\mathrm{4,1}} > 0$, ${\beta }_{\mathrm{7,4}} > 0$ ${\beta }_{\mathrm{4,1}} > 0$, ${\beta }_{\mathrm{7,4}}\le 0$ (or ${\beta }_{\mathrm{4,1}}\le 0$, ${\beta }_{\mathrm{7,4}} > 0$) ${\beta }_{\mathrm{4,1}}\le 0$, ${\beta }_{\mathrm{7,4}}\le 0$	H4A supported H4A partially supported H4A rejected	Siennick and Turanovic (2023), Fredrick et al. (2022), Bartlett et al. (2023)
	H4B: Friendship and social support positively predicts later friendship and social support	${\beta }_{\mathrm{5,2}} > 0$, ${\beta }_{\mathrm{8,5}} > 0$	${\beta }_{\mathrm{5,2}} > 0$, ${\beta }_{\mathrm{8,5}} > 0$ ${\beta }_{\mathrm{5,2}} > 0$, ${\beta }_{\mathrm{8,5}}\le 0$ (or ${\beta }_{\mathrm{5,2}}\le 0$, ${\beta }_{\mathrm{8,5}} > 0$) ${\beta }_{\mathrm{5,2}}\le 0$, ${\beta }_{\mathrm{8,5}}\le 0$	H4B supported H4B partially supported H4B rejected	Siennick and Turanovic (2023)
	H4C: Internalizing symptoms positively predict later Internalizing symptoms	${\beta }_{\mathrm{6,3}} > 0$, ${\beta }_{\mathrm{9,6}} > 0$	${\beta }_{\mathrm{6,3}} > 0$, ${\beta }_{\mathrm{9,6}} > 0$ ${\beta }_{\mathrm{6,3}} > 0$, ${\beta }_{\mathrm{9,6}}\le 0$ (or ${\beta }_{\mathrm{6,3}}\le 0$, ${\beta }_{\mathrm{9,6}} > 0$) ${\beta }_{\mathrm{6,3}}\le 0$, ${\beta }_{\mathrm{9,6}}\le 0$	H4C supported H4C partially supported H4C rejected	Siennick and Turanovic (2023), Fredrick et al. (2022), Bartlett et al. (2023)
H5: Peer relationships and mental health share a reciprocal relationship over time. Transactional model (Reijntjes et al., 2010; Sameroff & Mackenzie, 2003)			H1 *or* H2, *and* H3, supported or partially supported Any other outcome	H5 supported H5 rejected	LoParo et al. (2023), Siennick and Turanovic (2023), Fredrick et al. (2022), Bartlett et al. (2023), Boyes et al. (2014)
H6: Developmental cascades between peer relationships and mental health vary by gender. Gender socialization and intensification theory (Sravanti & Sagar Kommu, 2020)		Multi-group RI-CLPM analyses (chi-square difference tests and the test of small difference in fit) will indicate structural variance by gender	Multi-group RI-CLPM analyses indicate structural variance by gender Multi-group RI-CLPM analyses indicate structural invariance by gender	H6 supported, RI-CLPM conducted separately for boys and girls H6 rejected, single RI-CLPM conducted	Fredrick et al. (2022), Sentse et al. (2017)

## Methods

### Design and Sample

Secondary analysis of the longitudinal cohort of the #BeeWell dataset (#BeeWell Research Team, 2021) was undertaken. In brief, #BeeWell is a birth cohort study in which data pertaining to the domains and drivers of adolescent wellbeing are gathered on an annual basis via an online survey from a very large convenience sample of young people attending secondary schools across Greater Manchester, England. These data are subsequently linked to administrative information on socio-demographic characteristics (e.g., gender) drawn from Local Authority or school records. Ethical approval for #BeeWell was granted by the University of Manchester research ethics committee (Ref: 2021-11133-18965). Further information about the project is available at www.beewellprogramme.org.

A cross-lag panel design was used, drawing on three annual data points (T1, autumn 2021; T2, autumn 2022; T3, autumn 2023) for the three focal variables (bullying victimization, friendship and social support, and internalizing symptoms). A ‘drop in’ approach (Masselink et al., 2018) was utilized in order to optimize sample size, with any participant with at least one data point eligible for inclusion in the analysis. Those with missing values in covariates or missing all the measures used in the analysis were excluded. A final sample of *N* = 26,458 observations were utilized in the main analytical model. A pre-planned sensitivity analysis was undertaken in which only participants with at least 2 waves of data were included. Findings are reported in Supplementary Materials (Tables S13 and S21). Table 2 reports the characteristics of the study sample.

**Table 2:** Participant characteristics

Characteristic	Percentage	Number of observations
Gender		
Girls	51%	13,395
Boys	49%	13,063
SEN		
Yes	16%	4333
No	84%	22,125
FSM		
Yes	28%	7522
No	72%	18,936
Ethnicity		
White	65%	17,246
Minority	35%	9212

*SEN* special educational needs, *FSM* free school meal eligibility

### Measures

#### Bullying victimization

A three-item scale adapted from the Understanding Society Youth Questionnaire (Institute for Social and Economic Research, 2021) and the Health Behaviors in School-Aged Children survey (Currie et al., 2014) was used: (1) *How often do you get physically bullied at school? By this we mean getting hit, pushed around, threatened, or having belongings* stolen; (2) *How often do you get bullied in other ways at school? By this we mean getting called names, getting left out of games, or having nasty stories spread about you on purpose*; and, (3) *How often do you get cyber-bullied? By this we mean someone sending mean texts or online messages about you, creating a website making fun of you, posting pictures that make you look bad online, or sharing them with others*. Participants selected responses to these questions from four options focusing on frequency/repetition: *Not bullied at all*; *Not much (1–3 times in the last 6 months)*; *Quite a lot (more than 4 times in the last 6 months)*; and*, A lot (a few times every week)*. Bullying victimization was modeled as a latent variable derived from the resultant data. Internal consistency was good (T1 *α* = 0.72; T2 *α* = 0.75; T3 *α* = 0.79; longitudinal confirmatory factor analysis (CFA) indicated good fit to the data: CFI = 0.998, TLI = 0.994, RMSEA = 0.023, SRMR = 0.009).

#### Friendship and social support

A four-item friendship and social support scale derived from the Child and Youth Resilience Measure (Jefferies et al., 2019) was used: (1) *I get along with people around me*; (2) *People like to spend time with me*; (3)*I feel supported by my friends*; and, (4) *My friends care about me when times are hard (for example if I am sick or have done something wrong*. Participants selected responses to these statements from five options: *Not at all; A little; Somewhat; Quite a bit;* and*, A lot*. Friendship and social support was modeled as a latent variable derived from the resultant data. Internal consistency was excellent (T1 *α* = 0.84; T2 *α* = 0.85; T3 *α* = 0.86; longitudinal CFA indicated good fit to the data: CFI = 0.930, TLI = 0.881, RMSEA = 0.109, SRMR = 0.039).

#### Internalizing symptoms

The 10-item emotional difficulties subscale of the Me and My Feelings measure (Deighton et al., 2013) was used: (1) *I feel lonely*; (2) *I am unhappy*; (3) *Nobody likes me*; (4) *I cry a lot*; (5) *I worry when I am at* school; (6) *I worry a lot*; (7) *I have problems* sleeping; (8) I *wake up in the night*; (9) *I am shy*; and, (10) *I feel scared*. Participants selected responses to these statements from three options: *Never; Sometimes;* and*, Always*. Internalizing symptoms was modeled as a latent variable derived from the resultant data. Internal consistency was excellent (T1 *α* = 0.87; T2 *α* = 0.89; T3 *α* = 0.90; longitudinal CFA indicated good fit to the data: CFI = 0.928, TLI = 0.916, RMSEA = 0.056, SRMR = 0.034).

#### Socio-demographic variables

The following socio-demographic variables were obtained from the linked administrative data or school records: gender^1^ (0 = boys, 1 = girls), ethnicity (0 = White, 1 = UK minority ethnic group), free school meal eligibility (FSM; 0 = not eligible, 1 = eligible), and special educational needs (SEN; 0 = no SEN, 1 = identified as having SEN).

### Analytic Strategy

During preliminary analysis, data were screened for missing values, skewness and kurtosis (Kline, 2023). The Full Information Maximum Likelihood (FIML) method was used to address missing data, with variables identified as significant predictors of missingness used as auxiliary variables. Skewness and kurtosis values were calculated for each item to assess the normality of the distributions. The Maximum Likelihood estimator with Robust standard errors was implemented when absolute univariate skewness and kurtosis values exceeded 2.0 and 7.0, respectively (Finney & DiStefano, 2013).

**Fig. 2:**
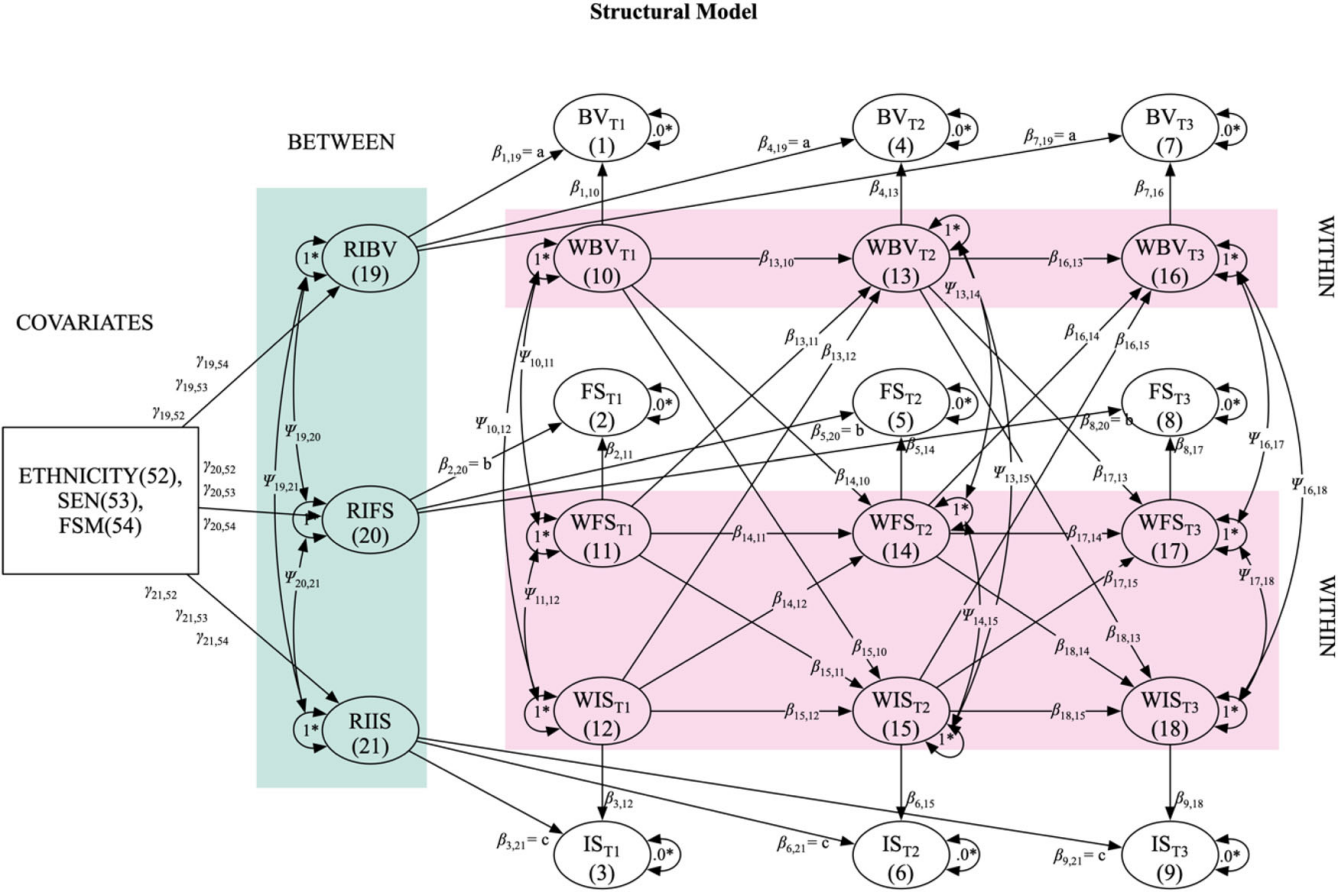
Statistical model for RI-CLPM developmental cascades analysis, including autoregressive effects (e.g., *β*_13,10_), within-person cross-lagged effects (e.g., *β*_13,11_,*β*_13,12_) and concurrent relationships (e.g., *Ψ*_13,14_). *Indicates a parameter fixed at the given value. BV bullying victimization, FS friendship and social support, IS internalizing symptoms. RI and W prefixes represent random intercepts and within components, respectively

The main analysis employed a multigroup random intercept cross-lagged panel model (RI-CLPM) across three measurement waves (see Figs. 1 and 2). This model examined the autoregressive and cross-lagged associations between bullying, friendship and social support, and internalizing symptoms. The model captured between-person effects through covariance between random intercepts and within-person effects through longitudinal lagged regressions and covariance between (residuals of) within-components. The analysis controlled for the potential influence of the following time-invariant sociodemographic variables as predictors of random intercepts: ethnicity, eligibility for free school meals (FSM), and special educational needs (SEN). For within-person cross-lagged path co-efficient values, 0.03, 0.07, and 0.12 were used as empirical benchmarks for small (25th percentile), moderate (50th percentile), and large (75th percentile) effects, as suggested in a recent meta-analysis that mapped the empirical distribution of such effects in 174 psychological studies using RI-CLPM and CLPM (Orth et al., 2024).

Figure 2 illustrates the RI-CLPM specification used. Model fit criteria included Tucker–Lewis index (TLI) and comparative fit index (CFI) values above 0.95, root mean square error of approximation (RMSEA) values below 0.08, and standardized root mean squared residual (SRMR) values below 0.10 (Schermelleh-Engel et al., 2003). Nested model comparisons employed chi-square tests, with significant Δ*χ*² statistics (*p* < 0.05) indicating significant changes in model fit.

The model building process consisted of three sequential steps. The first step employed longitudinal CFA to establish measurement invariance and the final measurement model. Comparisons between configural, metric, and scalar invariance measurement models utilized changes in model fit indices (ΔCFI and ΔRMSEA). The measurement model with more constraints (parsimony model) was selected when ΔCFI and ΔRMSEA fell below 0.01 and 0.015, respectively (Chen, 2007; Cheung & Rensvold, 2002). The second step estimated and compared two models: one with freely estimated parameters (Model 1) and another with within-effects parameters constrained equal across gender groups (Model 2). A chi-square difference test determined the necessity of a multigroup RI-CLPM. The third step compared the multigroup model (Model 1) with a simpler model (Model 3), constraining similarly sized congeneric within-person paths (difference <0.03, the empirical benchmarks for small effects in the RI-CLPM literature) to be equal over time. A chi-square difference test evaluated whether this time-invariant constraint significantly affected model fit and determined the final model.

All analyses were conducted in R 4.3 using structural equation modeling through the *semTools* package (Jorgensen et al., 2022) and the *lavaan* package (Rosseel, 2012). Full details of the study analytical procedures can be found in Supplementary Materials (Appendix A).

## Results

Little’s test for missingness across waves indicated that data were not missing completely at random, with significant, but low normed chi-square, at each timepoint (T1: *χ*²/df = 1.29, *p* < 0.001; T2: *χ*²/df = 1.29, *p* < 0.001; T3: *χ*²/df = 1.42, *p* < 0.001) and across all timepoints (*χ*²/df = 1.01, *p* = 0.013). Logistic regression analyses identified gender, special educational needs, free school meal eligibility, and ethnicity as significant predictors of missingness (see Table S2 in Supplementary Materials), supporting the missing at random assumption and justifying the use of FIML.

Initial data screening revealed high skewness and kurtosis in the first and third bullying victimization items (Table S1), supporting the implementation of the MLR estimator. Following confirmation of measurement unidimensionality, item parceling was applied to minimize the impact of correlated residuals in the measurement model (Appendix C, Supplementary Materials). The measurement testing established both longitudinal scalar invariance and multigroup metric invariance (see Supplementary Materials, Tables S4–S8). Descriptive statistics and bivariate correlations for the latent constructs, derived from the multi-group CFA, are presented in Table 3.

**Table 3:** Means, standard deviations and correlations between freely estimated latent variables for each group

Construct	BVT1	BVT2	BVT3	FST1	FST2	FST3	IST1	IST2	IST3
Girls									
BVT1	1.00								
BVT2	0.63	1.00							
BVT3	0.47	0.60	1.00						
FST1	−0.47	−0.33	−0.22	1.00					
FST2	−0.35	−0.46	−0.31	0.51	1.00				
FST3	−0.29	−0.34	−0.45	0.39	0.52	1.00			
IST1	0.56	0.38	0.28	−0.63	−0.45	−0.36	1.00		
IST2	0.39	0.52	0.34	−0.43	−0.61	−0.42	0.63	1.00	
IST3	0.33	0.39	0.51	−0.31	−0.41	−0.57	0.49	0.57	1.00
Latent Means	1.42	1.43	1.36	3.85	3.77	3.82	0.80	0.82	0.78
Latent Std. Dev.	0.51	0.54	0.51	0.79	0.81	0.79	0.41	0.42	0.43
Boys									
BVT1	1.00								
BVT2	0.53	1.00							
BVT3	0.36	0.47	1.00						
FST1	−0.48	−0.32	−0.24	1.00					
FST2	−0.29	−0.51	−0.29	0.50	1.00				
FST3	−0.26	−0.33	−0.48	0.40	0.52	1.00			
IST1	0.56	0.38	0.28	−0.60	−0.42	−0.35	1.00		
IST2	0.41	0.57	0.36	−0.41	−0.57	−0.42	0.57	1.00	
IST3	0.28	0.34	0.55	−0.29	−0.34	−0.54	0.42	0.49	1.00
Latent Means	1.35	1.38	1.34	3.85	3.80	3.77	0.53	0.50	0.48
Latent Std. Dev.	0.52	0.57	0.59	0.79	0.83	0.85	0.36	0.39	0.42

Results are based on the effects-coded method of identification with the scalar invariant across time and metric invariant across gender

*BV* bullying victimization, *FS* friendship and social support, *IS* internalizing symptoms, *T1, 2, 3* Time 1, 2, 3

### Structural (In)variance by Gender

Two multigroup RI-CLPM models were compared to test for gender differences: Model 1 with freely estimated parameters and Model 2 with parameters constrained to be equal across gender groups. Results revealed that constraining parameters to be equal across genders significantly reduced model fit (∆*χ*^2^ = 51.426, ∆*df* = 27, *p* = 0.003, see Table 4; see also Tables S11 and S12 in Supplementary Materials), providing clear evidence of structural variance by gender, and fully supporting Hypothesis 6. Consequently, all subsequent RI-CLPM analyses are presented separately for boys and girls.

**Table 4:** Model fits and comparisons for RI-CLPMs

Model	*χ*^2^	df	RMSEA (90% CI)	CFI	TLI	SRMR	∆*χ*^2^	∆df	*p*
	Model fits						Comparison with Model 1		
Model 1	5036.796	726	0.030(0.029, 0.031)	0.975	0.970	0.030	–	–	–
Model 2	5058.334	753	0.029(0.028, 0.030)	0.974	0.971	0.031	51.426	27	0.003
Model 3	5016.063	740	0.029(0.029, 0.030)	0.975	0.970	0.031	10.202	14	0.747

Model 1 = freely estimated parameters. Model 2 = within-effects parameters constrained equal across gender groups. Model 3 = time-invariant model with similarly sized congeneric within-person paths constrained equal. Model comparisons were conducted using chi-square difference tests, with *p* < 0.05 indicating significant changes in model fit.

### Model Fit

**Fig. 3:**
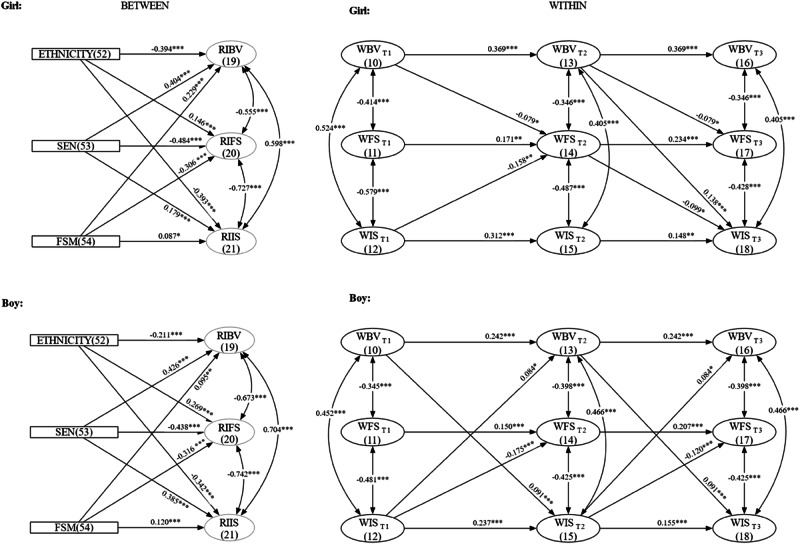
RI-CLPM developmental cascades results. The diagram shows statistically significant unstandardized autoregressive and cross-lagged paths and concurrent relationships for the multigroup RI-CLPM. The left panel shows the relationship between random intercepts and the effect of covariates on random intercepts. The right panel shows the autoregressive, cross-lagged paths and concurrent relationships between within components of the three constructs. For clarity, the graph displays only significant paths, though all were estimated in the model. The model is estimated as a partially stationary process. As such, equality constraints have been applied to congeneric paths that have similar effect sizes (difference less than the pre-registered small effect size) and are not significantly different from each other. BV bullying victimization, FS friendship and social support, IS internalizing symptoms. RI and W prefixes represent random intercepts and within components, respectively. *p < 0.05; **p < 0.01; ***p < 0.001

Following the analysis plan, two models were compared: a baseline multigroup RI-CLPM model with freely estimated parameters (Model 1) and a more parsimonious (Model 3) with similarly sized congeneric paths constrained to be equal over time. Both models demonstrated good fit to the data, with CFIs > 0.95, TLIs > 0.95, RMSEAs < 0.050, and SRMRs < 0.050 (see Table 4). The nested model comparison revealed that the constraints in the parsimonious model did not significantly reduce model fit (∆*χ*^2^ = 10.202, ∆*df* = 14, *p* = 0.75). Consequently, more parsimonious Model 3 was selected. This final multigroup RI-CLPM model (Model 3) showed excellent fit to the data (Table 4). The complete results are presented in Fig. 3, which illustrates the statistically significant autoregressive effects, cross-lagged pathways, concurrent relationships, and covariate effects on random intercepts (see also Table S22). Additional statistical details, including *p*-values, 95% confidence intervals, and standardized coefficients, are reported in Tables 5 and 6.

**Table 5:** Path coefficients of the associations among within-person components of bullying victimization, friendships and social support, and internalizing symptoms

		Girls					Boys				
Path	Time	*b*	*p*	CI	Beta	Hypotheses	*b*	*p*	CI	Beta	Hypotheses
		Cross-lagged pathways									
BV → FS	T1 → T2	−0.08*	0.04	[−0.15, −0.01]	−0.07	H1A supported	0.07	0.13	[−0.02, 0.15]	0.06	H1A rejected
BV → FS	T2 → T3	−0.08*	0.04	[−0.15, −0.01]	−0.08	H1A supported	−0.03	0.45	[−0.10, 0.05]	−0.03	H1A rejected
BV → IS	T1 → T2	0.05	0.30	[−0.04, 0.13]	0.04	H1B partially supported	0.09**	0.007	[0.02, 0.16]	0.09	H1B supported
BV → IS	T2 → T3	0.14***	<0.001	[0.06, 0.22]	0.14	H1B partially supported	0.09**	0.007	[0.02, 0.16]	0.09	H1B supported
FS → BV	T1 → T2	−0.05	0.17	[−0.13, 0.02]	−0.05	H2A rejected	0.00	1.00	[−0.06, 0.06]	0.00	H2A rejected
FS → BV	T2 → T3	−0.05	0.17	[−0.13, 0.02]	−0.05	H2A rejected	0.00	1.00	[−0.06, 0.06]	0.00	H2A rejected
FS → IS	T1 → T2	−0.07	0.10	[−0.15, 0.01]	−0.06	H2B partially supported	−0.04	0.27	[−0.10, 0.03]	−0.03	H2B rejected
FS → IS	T2 → T3	−0.10*	0.01	[−0.18, −0.02]	−0.10	H2B partially supported	−0.04	0.27	[−0.10, 0.03]	−0.04	H2B rejected
IS → BV	T1 → T2	0.00	0.92	[−0.08, 0.07]	0.00	H3A rejected	0.08*	0.02	[0.01, 0.16]	0.08	H3A supported
IS → BV	T2 → T3	0.00	0.92	[−0.08, 0.07]	0.00	H3A rejected	0.08*	0.02	[0.01, 0.16]	0.08	H3A supported
IS → FS	T1 → T2	−0.16**	0.002	[−0.26, −0.06]	−0.15	H3B partially supported	−0.17***	<0.001	[−0.28, −0.07]	−0.17	H3B supported
IS → FS	T2 → T3	−0.08	0.09	[−0.16, 0.01]	−0.08	H3B partially supported	−0.12**	0.003	[−0.20, −0.04]	−0.12	H3B supported
		Autoregressive effects									
BV → BV	T1 → T2	0.37***	<0.001	[0.25, 0.48]	0.34	H4A supported	0.24***	<0.001	[0.16, 0.33]	0.23	H4A supported
BV → BV	T2 → T3	0.37***	<0.001	[0.25, 0.48]	0.37	H4A supported	0.24***	<0.001	[0.16, 0.33]	0.24	H4A supported
FS → FS	T1 → T2	0.17**	0.003	[0.06, 0.28]	0.16	H4B supported	0.15**	0.007	[0.04, 0.26]	0.15	H4B supported
FS → FS	T2 → T3	0.23***	<0.001	[0.13, 0.33]	0.23	H4B supported	0.21***	<0.001	[0.11, 0.30]	0.20	H4B supported
IS → IS	T1 → T2	0.31***	<0.001	[0.20, 0.42]	0.29	H4C supported	0.24***	<0.001	[0.14, 0.34]	0.23	H4C supported
IS → IS	T2 → T3	0.15**	0.002	[0.05, 0.24]	0.15	H4C supported	0.16***	<0.001	[0.07, 0.24]	0.16	H4C supported

*BV* bullying victimization, *FS* friendship and social support, *IS* internalizing symptoms, *RI* random intercept, *T1, 2, 3* Time 1, 2, 3

*p < 0.05; **p < 0.01; ***p < 0.001

**Table 6:** Relationships between bullying victimization, friendship and social support, and internalizing symptoms (includes between-person components random intercepts, and within-person components at each timepoint)

		Girls			Boys		
Path	Time	Beta	*p*	CI	Beta	*p*	CI
		Concurrent relationships between within-person components					
FS ~ BV	T1	−0.41***	<0.001	[−0.48, −0.34]	−0.35***	<0.001	[−0.42, −0.27]
FS ~ BV	T2	−0.35***	<0.001	[−0.39, −0.31]	−0.40***	<0.001	[−0.44, −0.36]
FS ~ BV	T3	−0.35***	<0.001	[−0.39, −0.31]	−0.40***	<0.001	[−0.44, −0.36]
IS ~ BV	T1	0.52***	<0.001	[0.46, 0.59]	0.45***	<0.001	[0.38, 0.52]
IS ~ BV	T2	0.40***	<0.001	[0.37, 0.44]	0.47***	<0.001	[0.43, 0.51]
IS ~ BV	T3	0.40***	<0.001	[0.37, 0.44]	0.47***	<0.001	[0.43, 0.51]
IS ~ FS	T1	−0.58***	<0.001	[−0.63, −0.53]	−0.48***	<0.001	[−0.54, −0.42]
IS ~ FS	T2	−0.49***	<0.001	[−0.53, −0.44]	−0.43***	<0.001	[−0.46, −0.39]
IS ~ FS	T3	−0.43***	<0.001	[−0.47, −0.38]	−0.43***	<0.001	[−0.46, −0.39]
		Relationships between between-person components					
FS ~ BV	T1–T3	−0.55***	<0.001	[−0.66, −0.44]	−0.67***	<0.001	[−0.78, −0.57]
IS ~ BV	T1–T3	0.60***	<0.001	[0.52, 0.68]	0.70***	<0.001	[0.62, 0.79]
IS ~ FS	T1–T3	−0.73***	<0.001	[−0.80, −0.66]	−0.74***	<0.001	[−0.81, −0.67]

*BV* bullying victimization, *FS* friendship and social support, *IS* internalizing symptoms, *T1, 2, 3* Time 1, 2, 3

***p < 0.001

### Autoregressive Effects

The analysis revealed significant stability for all within-person constructs, with scores at each timepoint predicted by scores at the preceding timepoint (T1 → T2, T2 → T3; see Fig. 3 and Table 5). Among these temporal relationships, bullying victimization showed the strongest autoregressive effects, followed by internalizing symptoms, and then friendship and social support. This pattern of stability was similar for boys and girls. These findings fully supported Hypotheses H4A, H4B, and H4C.

### Bullying Victimization and Friendship and Social Support

The random intercepts for bullying victimization and friendship and social support showed significant negative correlations for both boys and girls (Fig. 3 and Table 6). This finding indicates that individuals who experienced higher stable levels of bullying victimization exhibited lower stable levels of friendship and social support. At the within-person level, significant negative concurrent correlations between bullying victimization and friendship and social support for both boys and girls were observed.

In terms of within-person cross-lagged effects (Table 5), higher levels of bullying victimization at both T1 and T2 predicted significant, moderate decreases in friendship and social support at subsequent time points (T2 and T3 respectively) for girls but not boys. Hypothesis 1A was fully supported for girls but rejected for boys. Friendship and social support did not significantly predict subsequent bullying victimization for either girls or boys, leading to the rejection of Hypothesis 2A. Consequently, no reciprocal relationship emerged over time, meaning that Hypothesis 5 was rejected for the relationship between bullying victimization and friendship and social support.

### Bullying Victimization and Internalizing Symptoms

The random intercepts for bullying victimization and internalizing symptoms showed strong positive correlations for both boys and girls (Fig. 3 and Table 6). This finding demonstrates that individuals who experienced higher stable levels of bullying victimization also exhibited higher stable levels of internalizing symptoms. At the within-person level, significant positive concurrent correlations between bullying victimization and internalizing symptoms for both boys and girls were also observed.

In terms of within-person cross-lagged effects (Table 5), boys’ bullying victimization predicted significant, moderate increases in their internalizing symptoms across both time lags (T1 → T2 and T2 → T3), while higher internalizing symptoms predicted significant, moderate increases in bullying victimization at both time points. These findings supported both Hypothesis 1B and Hypothesis 3A for boys. Consequently, a reciprocal relationship between internalizing symptoms and bullying victimization was evident, supporting Hypothesis 5.

For girls, bullying victimization predicted significant, large increases in internalizing symptoms only at T2 → T3, and internalizing symptoms did not predict subsequent bullying victimization. Hypothesis 1B was partially supported for girls, while Hypothesis 3A was not supported.

### Internalizing Symptoms and Friendship and Social Support

The random intercepts for internalizing symptoms and friendship and social support showed significant negative correlations for both boys and girls (Fig. 3 and Table 6). This finding indicates that individuals who experienced higher stable levels of internalizing symptoms exhibited lower stable levels of friendship and social support. At the within-person level, significant negative concurrent correlations between internalizing symptoms and friendship and social support were observed for both boys and girls.

In terms of within-person cross-lagged effects (Table 5), higher internalizing symptoms at T1 predicted large decreases in friendship and social support at T2 for both boys and girls. This predictive relationship persisted from T2 to T3 only for boys, fully supporting Hypothesis 3B for boys while providing partial support for girls.

The influence of friendship and social support on subsequent internalizing symptoms was moderate and evident only for girls, from T2 to T3. Hypothesis 2B was partially supported for girls but rejected for boys. The combined partial support for both Hypotheses 3B and 2B in girls demonstrated a reciprocal relationship between internalizing symptoms and friendship and social support over time, supporting Hypothesis 5.

### Sensitivity Analyses

#### Multiple imputation and sample specification

A sensitivity analysis using multiple imputation provided an alternative approach to handling missing data. The analysis employed predictive mean matching (PMM) on wide format data, generating fifty imputed datasets. The analysis was conducted on each imputed dataset, followed by result pooling. Results demonstrated substantial consistency with the main FIML analysis findings. The statistical significance of key effects remained stable across both methods. Detailed results of using multiple imputation on samples with at least one and two waves of data are presented in the supplementary materials (Tables S13 and S14). The consistent pattern of statistical significance across both approaches, despite trivial changes in effect sizes, strengthens the robustness of the study’s conclusions.

#### Measurement model and other factors

Results obtained from the main analyses were also were largely insensitive to 1) using longitudinal residual and gender group metric invariance measurement model (Table S15); 2) using original indicators without parceling in the measurement model (Table S16); 3) using manifest variables (averaging the original indicators) in the measurement model (Table S17); 4) using an alternative RI-CLPM identification approach (Table S18); 5) not constraining congeneric paths to be equal (Table S19); 6) when adjusting the standard errors by including school as a clustering factor (Table S20); and, 7) when use the samples with at least two waves of data with FIML (Table S21).

## Discussion

Current understanding of the longitudinal relationships between different aspects of peer relationships and mental health problems in early- to mid-adolescence is limited. In particular, the role played by gender in these developmental cascades processes is unclear, little is known about within-person effects between bullying victimization and internalizing symptoms, and the theorized benefits of friendship and social support are largely untested. Addressing these important research gaps, this study tested a number of theory-driven hypotheses (e.g., interpersonal risk model, transactional model) regarding longitudinal relationships between bullying victimization, friendship and social support, and internalizing symptoms. Separating within-person effects from between-person effects, RI-CLPM analyses revealed distinct, gender-specific pathways (e.g., higher levels of internalizing symptoms led to increased rates of bullying victimization for boys only; higher levels of friendship and social support lead to reduced internalizing symptoms for girls only), meaning that support for the different underpinning models varied by gender.

### Developmental Cascades Between Peer Relationships and Mental Health Vary by Gender (Hypothesis 6)

Hypothesis 6 is addressed first, since this impacts how all other findings are reported. The multigroup RI-CLPM favored the model which did not set group equality constraints on the structural model regression coefficients and concurrent effects across the two groups (boys and girls) over the model in which those structural coefficients were constrained to be identical across these groups (Mulder & Hamaker, 2021), indicating that the within-person cross-lagged effects among the three variables of interest and autoregression relationship within each varied by gender. Hypothesis 6, and the gender socialization and intensification theory underpinning it was fully supported (Sravanti & Sagar Kommu, 2020). These findings align with the notion that gender is an important moderator of mental health, peer relationships, and the association between them in adolescence (Van Droogenbroeck et al., 2018). The results corroborate some existing RI-CLPM evidence that reported developmental cascade pathways between bullying victimization and internalizing symptoms varied across gender (although, as noted later, the exact nature of these gender differences was not in full alignment with the findings of the current study; Fredrick et al., 2022). More generally, these findings contribute to a developing evidence base concerning the role that gender plays in developmental cascades in childhood and adolescence (e.g., research on gender differences in cascade paths between internalizing symptoms, externalizing problems, and academic attainment in middle childhood; Panayiotou & Humphrey, 2018).

### Peer Relationships and Mental Health are Stable Within-Persons Over Time (Hypothesis 4)

Consistent with developmental cascades theory (Masten & Cicchetti, 2010) and prior research, there was considerable stability over time in friendship and social support (Siennick & Turanovic, 2023), bullying victimization (Fredrick et al., 2022), and internalizing symptoms (Fredrick et al., 2022). Hypothesis 4 was fully supported. Of particular note is that bullying victimization yielded the largest autoregressive effect sizes, with consequent implications for prevention and intervention (see *Implications* subsection). Autoregressive effects for friendship and social support strengthened across lags, which might be indicative of friendship groups stabilizing as young people progress through secondary school (Meter & Card, 2016).

### Bullying Victimization has Adverse Consequences for Later Peer Relationships and Mental Health (Hypothesis 1)

The hypothesis that bullying victimization would predict later internalizing symptoms (H1B) and its underpinning interpersonal risk model was supported (Coyle et al., 2021). These findings offer a number of insights that are indicative of a causal relationship (Stewart, 2020). First and foremost, the interpersonal risk model offers a *plausible* explanation (see *Peer Relationships, Internalizing Symptoms, and Gender in Adolescence: Theoretical Perspectives*) for why bullying victimization would cause increased internalizing symptoms. Second, as noted elsewhere, the use of RI-CLPM enabled the separation of within-person effects from between-person effects. Within-person estimates offer increased precision vis-à-vis situational change within individuals, in addition to establishing clear *temporal precedence*, while also accounting for reverse causality (i.e., that internalizing symptoms could predict later bullying victimization). Third, findings were *consistent* across gender models and time lags (T1 → T2 and T2 → T3 for boys, and T2 → T3 for girls), and also in alignment with prior research (e.g. Bartlett et al., 2023; Sentse et al., 2017). Finally, the *strength* of cross-lag path coefficients (which ranged from *β* = 0.09 to *β* = 0.14) were in the moderate-to-large range relative to the empirical distribution of cross-lagged effects in existing studies (i.e., between the 50th and 75th percentiles or higher; Orth et al., 2024). This is particularly noteworthy given the temporal stability of both bullying victimization and internalizing symptoms, and their significant between-person association (Adachi & Willoughby, 2015), in addition to the fact that the developmental period assessed in the current study overlaps with the peak age of onset for internalizing disorders (Solmi et al., 2022). Collectively, these findings implicate bullying victimization as a key causal factor in the increased vulnerability to the development of internalizing symptoms in early-to-mid adolescence.

The related prediction that bullying victimization would undermine later friendship and social support (H1A) was fully supported for girls but not for boys, with the consequent implications for both theory (i.e., further evidence of gender as an important moderator of mental health, peer relationships, and the developmental associations between them; Van Droogenbroeck et al., 2018) and practice (see *Implications*). These findings align with the interpersonal risk model, in which it is theorized that bullying victimization will have a negative impact on friendships (Coyle et al., 2021). One possible explanation for the fact that effects were only found for girls relates to the nature of victimization experienced. Girls are significantly more likely to be exposed to *relational* bullying than boys, while boys are significantly more likely to be exposed to *physical* bullying than girls (Thornton et al., 2024). It is plausible that this gender difference explains the findings reported here, as by its very nature, relational bullying ostracizes and isolates the victim. Further research which examines potential effects of various forms of bullying behavior (e.g., physical, relational, cyber) on different aspects of friendship and social support (e.g., feeling supported, getting along, and spending time with friends) and the extent to which these vary by gender would help to elucidate the exact processes at play in the interpersonal risk model. Given the more nuanced focus of such a study, it would need to take the form of item-level cross-lagged panel network analysis as opposed to latent variable modeling (e.g., Ren et al., 2023).

The strength of developmental cascade pathways between bullying victimization and friendship and social support for girls was moderate relative to the empirical distribution of cross-lagged effects in existing studies (i.e., at the 50th percentile; Orth et al., 2024). As above, this is particularly noteworthy given the relative temporal stability of these constructs and their significant between-person association (Adachi & Willoughby, 2015). Given the relatively limited robust evidence of such within-person effects prior to the current study (the only exception aligning with the findings of the current study; Siennick & Turanovic, 2023), replication should be a priority for future research.

### Positive Peer Relationships Confer Protection Against Later Bullying Victimization and Internalizing Symptoms (Hypothesis 2)

Contrary to expectations, the prediction that friendship and social support would inversely predict later bullying victimization (H2A) was not supported. These findings, which were consistent across gender models and time lags, cast doubt on the notion that friendship and peer support directly reduce later instances of bullying victimization, and appear to contradict one of the only other studies that has robustly examined the within-person relationship between these constructs, which found reciprocal negative relationships between friendship support and bullying victimization (Siennick & Turanovic, 2023). Given the sparse evidence available, further research using advanced statistical models (e.g., RI-CLPM) that can provide further clarification is essential. An alternative explanation is that rather than directly reducing later instances of bullying victimization, increased friendship and social support instead confer compensatory effects, increasing resilience and coping in the face of such adverse social experiences (Murray et al., 2021). There is tentative evidence to support this proposition, but only in girls, where friendship and social support reduced later internalizing symptoms from T2 → T3, providing partial support for H2B.

### Mental Health Difficulties Precede Relational Difficulties With Peers (Hypothesis 3)

The hypothesis that internalizing symptoms would negatively predict later friendship and social support (H3B) was fully supported for boys (T1 → T2, T2 → T3) and partially supported for girls (T1 → T2). Path co-efficient effect sizes were large relative to the empirical distribution of cross-lagged effects in existing studies (i.e. above the 75th percentile; Orth et al., 2024). These findings are consistent with the symptoms-driven model (Kochel et al., 2012). One explanation is that individuals experiencing elevated internalizing symptoms may be more likely to be socially withdrawn, leading to increased likelihood of peer rejection. In support of this, one study found that depression predicted later peer rejection in a similarly aged sample to that used here (Beeson et al., 2020).

The hypothesis that internalizing symptoms would predict later bullying victimization (H3A), an extreme example of peer rejection, was supported only for boys, for whom the associated path coefficients were indicative of a moderate effect (Orth et al., 2024). This suggests that gender may moderate the relationship between mental health difficulties and some forms of relational peer difficulties (such as bullying) but not others (such as lower levels of friendship and social support). Age and the particular type of mental health difficulty experienced may also be important. For example, one study found that depression and anxiety both predicted peer rejection between ages 7.5 and 12, and 15 and 18, but only anxiety predicted peer rejection between 12 and 15 (LoParo et al., 2023). Use of a broadband internalizing symptoms measure did not allow us to distinguish different forms of internalizing mental health difficulties, and this may have influenced the findings.

The above issues notwithstanding, the finding that internalizing symptoms led to increased bullying victimization among boys but not girls may reflect socialization processes pertaining to male gender roles (e.g., success, power and competition; restrictive emotionality; restrictive affectionate behavior; Peate, 2020) and the associated notions of ‘toxic masculinity’ and ‘man up’ culture (i.e., negative aspects of exaggerated masculine traits, to which boys and men feel a pressure to conform as a result of cultural or societal expectations; Young Minds, 2022). In this context, boys who experience elevated internalizing symptoms may be seen as weak and ideal targets for victimization. Future studies should examine these constructs to determine if this theorization is supported empirically.

### Peer Relationships and Mental Health Share a Reciprocal Relationship Over Time (Hypothesis 5)

Regarding reciprocal relationships between the three constructs under investigation (Hypothesis 5), we found that bullying victimization and internalizing symptoms were reciprocally related for boys within (e.g., bullying victimization at T1 predicted internalizing symptoms at T2, and vice versa) and across (e.g., internalizing symptoms at T1 predicted bullying victimization at T2, which in turn predicted internalizing symptoms at T3) both lags, but not for girls. This suggests that for boys there is a dynamic and bidirectional relationship between internalizing symptoms and bullying victimization consistent with the transactional model (Sameroff & Mackenzie, 2003). These findings contrast with CLPM studies which have found reciprocal relationships for both boys and girls between internalizing symptoms and bullying victimization (Boyes et al., 2014), and between depression (but not social anxiety) and peer victimization (Bartlett et al., 2023). Another study reported a reciprocal relationship between anxiety and bullying victimization for girls, but not boys, and found no evidence of reciprocal associations between anxiety and bullying victimization for boys or girls (Sentse et al., 2017). The studies all looked at between- rather than within-person effects (Bartlett et al., 2023; Boyes et al., 2014; Sentse et al., 2017). In contrast, research has found some support for a within-person reciprocal relationship between depression and peer victimization overall, though this was not significant when considering only boys. For girls, there was some evidence of a reciprocal relationship between depression and peer victimization, but this was over the course of three waves (that is, peer victimization at T1 predicted depression at T2, which in turn predicted peer victimization at T3, rather than reciprocal relationships within the same lag) (Fredrick et al., 2022). The difference between these findings and those reported in the current study could be due to different ages in the samples at baseline (12–13 vs 13–15 years), or that different aspects of internalizing symptoms are differentially associated with bullying victimization (a notion lent support by a recent cross-lag panel network analysis; Ren et al., 2023).

Offering further support for H5 and the transactional model, and in contrast to some earlier findings (e.g., Murray et al., 2021), analyses also yielded evidence of a reciprocal relationship between internalizing symptoms and friendship and social support across lags (i.e., internalizing symptoms at T1 predicted friendship and social support at T2, which in turn predicted internalizing symptoms at T3) in girls, but not boys. The fact that this relationship was not found within lags prompts consideration of a distinct cascade sequence for girls in which, consistent with the symptoms-driven model (Kochel et al., 2012) and evidence noted above (Beeson et al., 2020), the emergence of symptoms at T1 drives social withdrawal and isolation at T2, which in turn reinforces and elevates internalizing symptoms at T3. As already noted, these T3 symptoms are also driven by bullying victimization at T2, which is concurrently inversely associated with friendship and social support.

In sum, findings support the transactional model of reciprocal associations between peer relationships and internalizing difficulties, but these vary by gender (i.e., girls vs boys), the type of peer relationships under consideration (i.e., bullying victimization vs friendship and peer support), and the nature of the reciprocal association (i.e., within vs across lags). For boys, bullying victimization was found to be reciprocally associated with internalizing symptoms both within and across lags, whereas for girls, internalizing symptoms were reciprocally associated with friendship and social support across, but not within lags.

### Study Strengths and Limitations

The current study has a number of strengths which give confidence in the robustness of findings. It benefitted from a very large sample (nearly double that of the largest sample of c.14,000 in a recent meta-analysis of CLPM and RI-CLPM studies; Orth et al., 2024), longitudinal design, an advanced statistical modeling approach (RI-CLPM) that enabled us to separate between- and within-person effects, and the inclusion of covariates to increase precision of estimates. The analysis was pre-registered, tested theoretically-informed hypotheses, and included sensitivity analyses to determine the impact (or lack thereof) of a series of researcher-led analytic choices (e.g., sample inclusion criteria). The use of annual data points enabled a nuanced, focused assessment of cascade processes during a demonstrably sensitive period for both peer relationships and internalizing symptoms.

There were also a number of limitations. First, the study sample was not nationally representative. Greater Manchester notably has higher levels of socio-economic deprivation and greater ethnic diversity than is seen across England, meaning caution is required when considering the generalizability of findings (though it is noteworthy that the analyses controlled for ethnicity and free school meal eligibility). Second, the three focal variables were all captured via self-report. Common method variance may have influenced findings, but this should be largely absorbed by the between-person components in the RI-CLPM, since these capture stable differences between individuals. This limitation also needs to be balanced against the fact that self-report of constructs such as internalizing symptoms is arguably the optimal, most valid method (Black et al., 2023); in other words, a multi-informant approach to measurement is not necessarily preferable in this context. Third, specific types of internalizing problems (e.g., depression, anxiety) may relate to bullying victimization and friendship and social support in different ways. The use of a broadband internalizing symptoms measure meant that these potential differential relationships could not be assessed in the current study, whose secondary data analysis relied on the measures available in the #BeeWell study. Finally, findings appeared to indicate potential mediation effects (e.g., BV → IS → FS in boys) but these were not tested, because while this is statistically possible in RI-CLPM, there are theoretical concerns (e.g., clarifying options for defining a direct effect in the cross-lag model; evaluation of causal identification assumptions pertaining to exchangeability, consistency, and positivity) that make it difficult to interpret mediation estimates (Mulder & Hamaker, 2023). Provided such concerns can be adequately addressed, future research could address the question of mediation effects.

### Implications

The findings of the current study indicate that the prevention of bullying should be prioritized and would be particularly beneficial in terms of addressing the significant increase in internalizing symptoms that has been observed in the transition from early- to mid- adolescence (Scott et al., 2013; Thornton et al., 2024). As the most stable of the constructs assessed in the current study, it is a ‘sticky’ problem, requiring a comprehensive, whole-school response comprising work with peers, bullies and victims; involvement of parents and teachers; and implementation of classroom rules and curriculum materials (Gaffney et al., 2019). Importantly, meta-analytic evidence indicates that anti-bullying interventions can successfully reduce perpetration, victimization *and* mental health problems (Fraguas et al., 2021; Gaffney et al., 2019). The latter outcome is particularly important because it provides *experimental* evidence in support of the causal relationship between bullying victimization and internalizing symptoms (Stewart, 2020).

The clear gender differences identified in cascade pathways indicate the need for prevention and intervention efforts to take account of gender socialization and intensification processes in adolescence. The current evidence base offers both promise and challenge. In relation to boys, and in particular the finding that internalizing symptoms predicted later bullying victimization, earlier identification of the former through universal screening may be useful in order to put in place targeted support to prevent or reduce the effects of the latter, but better resource allocation from education, health, and government agencies is needed to make the widespread adoption of such approaches more feasible (Soneson et al., 2020). With regard to girls, the finding that bullying victimization led to reduced friendship and social support, alongside the reciprocal relationship between internalizing symptoms and friendship and social support, indicates the centrality of the latter construct in developmental cascade processes in early-to-mid adolescence. Social support deserves tentative consideration as a plausible tailored intervention target for girls (i.e., improving social support as a preventive strategy, but particularly for those who are victims of bullying); a recent review gives an overview of a promising evidence base, with interventions providing or mobilizing social support through a variety of means (e.g., modeling healthy relationships and social skills; offering safe spaces or opportunities for adolescents to practice said skills; encouraging help-seeking for social support; and/or changing perceptions of the benefits of social support), and over half of reviewed studies reporting improved outcomes (including some pertaining to mental health as well as social support) (Bauer et al., 2021).

## Conclusion

The role played by gender in developmental cascades processes between peer relationships and mental health problems in early- to mid-adolescence is unclear, and little is known about within-person effects between bullying victimization and internalizing symptoms, or the theorized benefits of friendship and social support Addressing these important research gaps, this study tested a number of theory-driven hypotheses (e.g., symptoms-driven model, interpersonal risk model) regarding longitudinal relationships between bullying victimization, friendship and social support, and internalizing symptoms. Separating within-person effects from between-person effects, RI-CLPM analyses revealed distinct, gender-specific pathways. For example, higher levels of internalizing symptoms led to increased rates of bullying victimization for boys only, whereas higher levels of friendship and social support lead to reduced internalizing symptoms for girls only. This meant that support for the different underpinning models varied by gender. The findings of the current study highlight the moderating role of gender in developmental cascade processes, and indicate that approaches to prevent or reduce the effects of bullying victimization should be prioritized, given the consistent evidence of its substantial role in increasing internalizing symptoms for both girls and boys, in addition to its deleterious impact on girls’ friendship and social support.

## Supplementary information

Supplementary Materials

## Footnotes

1 The linked administrative data used refers to ‘female’ and ‘male’, language which is recognized as being more strongly associated with biological sex than gender. ‘Girls’ and ‘boys’ are used here in alignment with recommended nomenclature for gender (APA, 2020), and given that Hypothesis 6 pertains to participants’ gender (i.e., social construction and intensification of attitudes, feelings and behaviors based on a person’s biological sex by family, schools, peer groups, and the media) as opposed to their biological sex.
