# Supplementary material for: Longitudinal Relationships Across Bullying Victimization, Friendship and Social Support, and Internalizing Symptoms in Early-to-Middle Adolescence: A Developmental Cascades Investigation
Source: J Youth Adolesc. 2025 Jan 18;54(6):1377–95. doi: 10.1007/s10964-024-02131-2 (PMC12137435; doi:10.1007/s10964-024-02131-2)
Supplement: Supplementary file 1 — Supplementary Materials [file 10964_2024_2131_MOESM1_ESM.docx]

**Longitudinal Relationships Across Bullying Victimization, Friendship and Social Support, and Internalizing Symptoms in Early-to-Middle Adolescence: A Developmental Cascades Investigation**

**:** **Supplementary Materials**

Supplementary materials include the following appendices: Appendix A) Detailed Analysis Strategy; B) Data Screening; C) Item Parceling; D) Measurement Invariance Results; E) Baseline RI-CLPM; F) Structure Variance by Gender; G) Sensitivity Analysis: Multiple Imputations; H) Sensitivity Analysis: Measurement Models and Others; I) Measurement Invariance Syntax, J) RI-CLPM Syntax; K) RI-CLPM Output; and Appendix L) Effects of Covariates on Random Intercepts of Bullying, Friendship and Social Support, and Internalizing Symptoms.

# Appendix A) Detailed Analysis Strategy

All analyses were performed in *R 4.3*. Measurement invariance testing and the RI-CLPM analysis were performed through structural equation modelling, using the *semTools* package (Jorgensen et al., 2022) and the *lavaan* package (Rosseel, 2012). Satisfactory model fit was indicated by Tucker–Lewis index (TLI) and comparative fit index (CFI) values above .95, root mean square error of approximation (RMSEA) values below .08, and standardized root mean squared residual (SRMR) values below .10 (Schermelleh-Engel et al., 2003). For nested model comparisons, a chi-square test was conducted. Additionally, due to the sensitivity of the chi-square test, ΔCFI and ΔRMSEA were used in measurement invariance test. When ΔCFI and ΔRMSEA were below 0.01 and 0.015, respectively, the measurement model with more constraints was preferred and selected (Chen, 2007; Cheung & Rensvold, 2002).

Ordinal indicator variables were treated as (quasi) continuous. While some authors suggest that if the number of response categories is small (e.g. fewer than 5 categories) or the frequency distributions exhibit substantial asymmetry, ordinal indicators should be treated as such (Rhemtulla et al., 2012), others argue that the use of the two competitive modelling strategies is equally defensible (Robitzsch, 2020), and our own analyses have indicated that they produce analogous results (Marquez, et al., 2023). Syntax for the measurement invariance analysis and the RI-CLPM analysis is presented below (Appendix I and J, respectively).

**Data Screening.** Data were screened for missing values, skewness and kurtosis (Kline, 2023). Little’s test was conducted to determine if data was missing completely at random (MCAR) (Little, 1988). If this test indicated that data were not MCAR, we planned to perform a binary logistic regression to examine whether data are missing at random (MAR; that is, conditional on other observed variables), with odds ratios pertaining to missingness reported for gender, ethnicity, FSM, and SEN. We used Full Information Maximum Likelihood (FIML) to account for missing data in the main analyses, with any variables found to predict missingness utilized as auxiliary variables.

For the normality analysis, we checked if the data were highly skewed. If absolute univariate skewness and kurtosis values were greater than 2.0 and 7.0 respectively (Finney & DiStefano, 2013), the Maximum Likelihood estimator with Robust standard errors (MLR estimator) was used.

**Measurement Invariance.** We examined the longitudinal measurement invariance of the latent constructs of bullying, friendship and social support, and Internalizing symptoms before constructing the RI-CLPM model. The gender measurement invariance of these constructs was also explored before testing for structural invariance by gender (see *Structural (In)Variance by Gender* below). For this step, we fit all the configural invariance, the metric invariance, and the scalar invariance models with confirmatory factor analysis. This represents a minor deviation from our pre-registration, in which we had planned to also use residual invariance. By not using residual invariance but scalar invariance, we allow for some wave-specific variance in each wave; the residual invariance test is too restrictive and does not provide "better" evidence of invariance. Nonetheless, in the interest of transparency, we provide the results with residual invariance below (Table S15).

Nested model comparisons were conducted to determine measurement invariance and select the measurement model for the following analysis.

**RI-CLPM.** The autoregressive and reciprocal associations between bullying, friendship, and Internalizing symptoms were examined over the three measurement waves using RI-CLPM (Figure 1 and Figure 2). The covariance between random intercepts captures between-person effects with RI-CLPM, and lagged regressions capture longitudinal within-person effects. Figures 2 shows the specification of the RI-CLPM used in the current study. The measurement part of the RI-CLPM uses the constraints from the preferred measurement invariance model. Overall, the RI-CLPM analysis was conducted following Hamaker et al.’s (2015) recommended procedure.

We tested if the within-person effects are stationary (time-invariant) to achieve a more parsimonious RI-CLPM (Orth et al., 2021). We first estimated a baseline model that allows within-person paths to vary over time. This baseline model was compared with more simple models in which the similarly sized congeneric (difference less than the pre-registered small effect size) within-person paths were constrained to be equal over time. We looked at the simple models with autoregression effects (e.g., set the effect of T1 bullying on T2 bullying and the effect of T2 bullying on T3 bullying to be equal), cross-lagged effects (e.g., set the effect of T1 bullying on T2 Internalizing symptoms and the effect of T2 bullying on T3 Internalizing symptoms to be equal) and concurrent effects (e.g., set the concurrent association between T2 bullying and T2 Internalizing symptoms equal to that between T3 bullying and T3 Internalizing symptoms) being constrained to be equal step by step. Models were evaluated against the aforementioned indices, and nested models were compared using the aforementioned methods and criteria, and results reported below (Table S10).

**Structural (In)Variance by Gender**. To determine whether the within-person associations among the three variables of interest and autoregression relationship within each variable vary by gender, we employed the multi-group RI-CLPM method. We compared a multiple group RI-CLPM in which we did not set group equality constraints on the structural model regression coefficients and concurrent effects across the two groups (boys and girls) with another model in which we constrained those structural coefficients to be identical across these groups (Mulder & Hamaker, 2021). The comparison was evaluated by the chi-square difference tests and the results are reported below (Table S11, S12). All of the analysis to investigate gender differences was based on the freely estimated multigroup model, as well as the parsimony obtained in the previous section. If these procedures provided evidence of structural variance by gender, Hypothesis 6 was considered to be supported, and we would thus conduct RI-CLPM separately for boys and girls.

**Interpretation of Between and Within Person Effects.** Following Hamaker (2023), in this paper we define the between-person component as a person’s stable and constant trait that will not change within the duration of a given study and define within-person component as those time-varying fluctuations within a person. Therefore, the between-person effect in RI-CLPM was interpreted as associations among those stable traits at the individual level and the within-person cross-lagged effects were interpreted as the effect of a temporary deviation of construct X from a person’s stable trait at time $t$ on the temporary deviation of construct Y from that person’s stable trait at time $t+1$.

Based on this interpretation, we use Cohen’s r thresholds to assess between-person effect sizes (Cohen, 1992). For within-person cross-lagged path co-efficient values, we use 0.03, 0.07, and 0.12 as empirical benchmarks for small (25^th^ percentile), medium (50^th^ percentile), and large (75^th^ percentile) effects, as suggested in a recent meta-analysis that mapped the empirical distribution of such effects in 174 psychological studies using RI-CLPM and CLPM (Orth et al., 2022). The caveat in using these benchmarks is that various factors such as between-person effects, covariates, lag length and measurement type can influence cross-lagged and autoregressive effects. Furthermore, Adachi and Willoughby (2015) suggest that the significance of small longitudinal effects depends on bivariate correlations and the stability of the outcome. Accordingly, the indiscriminate application of thresholds is not advised. We therefore interpret cross-lagged parameters dynamically within the broader model, taking into account the above factors.

Considering that small effects should be the norm in the social sciences (Götz et al., 2022) and that even seemingly small cross-lagged within-person effects may accumulate over time to produce meaningful consequences, cross-lagged within-person effects of 0.03 or more are considered noteworthy in this study, which is at about the 25^th^ percentile in the Orth et al. (2022) sample. Alpha levels were set at 0.05 as indicative of statistical significance when analyzing distinct pathways and associations among variables in the RI-CLPM. Although Alpha was to set to a more conservative level (0.01) in our pre-registration, two factors influenced our ultimate decision to use the traditional 0.05 benchmark. First, Rubin (2021) argues that Alpha adjustment is not appropriate in the context of conjunctive hypothesis testing, in which all relevant results must be significant in order to fully reject the null hypothesis (as is the case in the current study). Second, RI-CLPM yields standard errors that are 1.3 to 2.6 times larger compared to CLPM, which leads to a lower power to detect a substantial and relevant effect. This is partly because models that separate between- and within-person effects reduce bias, but at the cost of efficiency (Grosz et al., 2021; Orth et al., 2021; Usami et al., 2019).

# Appendix B) Data Screening

Missing data across item-level survey data varied from 5.1 % to 6.1% at T1, 4.4% to 5.3% at T2, and 5.1% to 6.0 % at T3(Table S1). Little's MCAR test for the missingness in each time point indicated that data were not MCAR (T1: χ² = 6701.58, df = 5188, p < .001; T2: χ² = 5785.55, df = 4485, p < .001; T3: χ² = 6494.29, df = 4587, p < .001; All times: χ² = 56191.61, df = 55446, p = 0.013) and further logistic regressions suggested that gender, special educational needs, free school meal and ethnicity were significant predictors of missingness (Table S1), supporting the data as MAR and the use of FIML. As some of the items in the bullying construct have high skewness and kurtosis (Table S2), the MLR estimator was used in the analysis.

**Table S1.**

*Missing Data, Skewness, and Kurtosis of Indicators at Each Time Point*

|  | Missingness | |  |  |
| --- | --- | --- | --- | --- |
| Variable | N | % | Skewness | Kurtosis |
| Bullying (Item 1) T1 | 1031 | 5.52 | 2.25 | 7.75 |
| Bullying (Item 2) T1 | 1078 | 5.77 | 1.53 | 4.44 |
| Bullying (Item 3) T1 | 1083 | 5.80 | 2.71 | 10.49 |
| Friendships and Social support (Item 1) T1 | 1038 | 5.56 | -0.72 | 2.90 |
| Friendships and Social support (Item 2) T1 | 1233 | 6.60 | -0.64 | 2.69 |
| Friendships and Social support (Item 3) T1 | 1142 | 6.12 | -0.94 | 2.99 |
| Friendships and Social support (Item 4) T1 | 1119 | 5.99 | -0.90 | 2.81 |
| Internalizing Symptoms (Item 1) T1 | 944 | 5.05 | 0.30 | 2.32 |
| Internalizing Symptoms (Item 2) T1 | 1007 | 5.39 | -0.05 | 2.72 |
| Internalizing Symptoms (Item 3) T1 | 1110 | 5.94 | 0.89 | 2.78 |
| Internalizing Symptoms (Item 4) T1 | 1047 | 5.61 | 0.68 | 2.30 |
| Internalizing Symptoms (Item 5) T1 | 997 | 5.34 | 0.51 | 2.12 |
| Internalizing Symptoms (Item 6) T1 | 1037 | 5.55 | 0.39 | 1.86 |
| Internalizing Symptoms (Item 7) T1 | 998 | 5.34 | 0.52 | 1.86 |
| Internalizing Symptoms (Item 8) T1 | 1019 | 5.46 | 0.35 | 1.93 |
| Internalizing Symptoms (Item 9) T1 | 1020 | 5.46 | 0.54 | 2.18 |
| Internalizing Symptoms (Item 10) T1 | 1066 | 5.71 | 0.90 | 2.79 |
| Bullying (Item 1) T2 | 724 | 4.43 | 2.18 | 7.18 |
| Bullying (Item 2) T2 | 780 | 4.77 | 1.51 | 4.26 |
| Bullying (Item 3) T2 | 771 | 4.72 | 2.81 | 10.89 |
| Friendships and Social support (Item 1) T2 | 720 | 4.41 | -0.67 | 2.90 |
| Friendships and Social support (Item 2) T2 | 866 | 5.30 | -0.63 | 2.74 |
| Friendships and Social support (Item 3) T2 | 804 | 4.92 | -0.82 | 2.84 |
| Friendships and Social support (Item 4) T2 | 810 | 4.96 | -0.77 | 2.62 |
| Internalizing Symptoms (Item 1) T2 | 723 | 4.43 | 0.39 | 2.33 |
| Internalizing Symptoms (Item 2) T2 | 781 | 4.78 | 0.05 | 2.62 |
| Internalizing Symptoms (Item 3) T2 | 849 | 5.20 | 0.92 | 2.80 |
| Internalizing Symptoms (Item 4) T2 | 818 | 5.01 | 0.76 | 2.31 |
| Internalizing Symptoms (Item 5) T2 | 766 | 4.69 | 0.50 | 2.04 |
| Internalizing Symptoms (Item 6) T2 | 798 | 4.88 | 0.39 | 1.81 |
| Internalizing Symptoms (Item 7) T2 | 757 | 4.63 | 0.53 | 1.84 |
| Internalizing Symptoms (Item 8) T2 | 771 | 4.72 | 0.42 | 1.92 |
| Internalizing Symptoms (Item 9) T2 | 784 | 4.80 | 0.58 | 2.19 |
| Internalizing Symptoms (Item 10) T2 | 805 | 4.93 | 0.97 | 2.88 |
| Bullying (Item 1) T3 | 840 | 5.34 | 2.53 | 8.87 |
| Bullying (Item 2) T3 | 871 | 5.53 | 1.78 | 5.27 |
| Bullying (Item 3) T3 | 864 | 5.49 | 2.97 | 11.52 |
| Friendships and Social support (Item 1) T3 | 848 | 5.39 | -0.72 | 3.08 |
| Friendships and Social support (Item 2) T3 | 942 | 5.99 | -0.67 | 2.91 |
| Friendships and Social support (Item 3) T3 | 931 | 5.92 | -0.82 | 2.94 |
| Friendships and Social support (Item 4) T3 | 926 | 5.88 | -0.78 | 2.71 |
| Internalizing Symptoms (Item 1) T3 | 808 | 5.13 | 0.51 | 2.36 |
| Internalizing Symptoms (Item 2) T3 | 857 | 5.45 | 0.17 | 2.42 |
| Internalizing Symptoms (Item 3) T3 | 909 | 5.78 | 1.12 | 3.20 |
| Internalizing Symptoms (Item 4) T3 | 875 | 5.56 | 0.87 | 2.48 |
| Internalizing Symptoms (Item 5) T3 | 856 | 5.44 | 0.58 | 2.15 |
| Internalizing Symptoms (Item 6) T3 | 884 | 5.62 | 0.46 | 1.91 |
| Internalizing Symptoms (Item 7) T3 | 839 | 5.33 | 0.55 | 1.89 |
| Internalizing Symptoms (Item 8) T3 | 858 | 5.45 | 0.51 | 1.98 |
| Internalizing Symptoms (Item 9) T3 | 880 | 5.59 | 0.61 | 2.26 |
| Internalizing Symptoms (Item 10) T3 | 887 | 5.64 | 1.10 | 3.16 |

*Note.* T = Time point.

**Table S2.**

*Odds Ratios Pertaining to Missingness for Covariates for Bullying Victimization, Friendships and Social Support and Internalizing Symptoms.*

| Variable | GENDER | ETHNICITY | SEN | FSMEVER |
| --- | --- | --- | --- | --- |
| Bullying Victimization (Item 1) T1 | 0.9 | 1.5 *** | 1.55 *** | 1.32 *** |
| Bullying Victimization (Item 2) T1 | 0.94 | 1.49 *** | 1.58 *** | 1.34 *** |
| Bullying Victimization (Item 3) T1 | 0.93 | 1.47 *** | 1.51 *** | 1.31 *** |
| Friendships and Social support (Item 1) T1 | 0.88 | 1.49 *** | 1.59 *** | 1.34 *** |
| Friendships and Social support (Item 2) T1 | 0.92 | 1.48 *** | 1.63 *** | 1.34 *** |
| Friendships and Social support (Item 3) T1 | 0.86 * | 1.53 *** | 1.58 *** | 1.4 *** |
| Friendships and Social support (Item 4) T1 | 0.88 * | 1.57 *** | 1.6 *** | 1.32 *** |
| Internalizing Symptoms (Item 1) T1 | 0.69 *** | 1.62 *** | 1.65 *** | 1.19 * |
| Internalizing Symptoms (Item 2) T1 | 0.71 *** | 1.56 *** | 1.76 *** | 1.2 * |
| Internalizing Symptoms (Item 3) T1 | 0.8 *** | 1.63 *** | 1.77 *** | 1.23 ** |
| Internalizing Symptoms (Item 4) T1 | 0.75 *** | 1.56 *** | 1.67 *** | 1.16 * |
| Internalizing Symptoms (Item 5) T1 | 0.74 *** | 1.59 *** | 1.66 *** | 1.19 * |
| Internalizing Symptoms (Item 6) T1 | 0.78 *** | 1.57 *** | 1.66 *** | 1.18 * |
| Internalizing Symptoms (Item 7) T1 | 0.76 *** | 1.6 *** | 1.74 *** | 1.21 ** |
| Internalizing Symptoms (Item 8) T1 | 0.74 *** | 1.6 *** | 1.67 *** | 1.19 * |
| Internalizing Symptoms (Item 9) T1 | 0.75 *** | 1.67 *** | 1.82 *** | 1.18 * |
| Internalizing Symptoms (Item 10) T1 | 0.73 *** | 1.62 *** | 1.67 *** | 1.19 * |
| Bullying Victimization (Item 1) T2 | 0.97 | 1.2 * | 1.36 ** | 1.37 *** |
| Bullying Victimization (Item 2) T2 | 0.93 | 1.19 * | 1.3 ** | 1.3 *** |
| Bullying Victimization (Item 3) T2 | 0.92 | 1.18 * | 1.29 ** | 1.34 *** |
| Friendships and Social support (Item 1) T2 | 0.9 | 1.17 * | 1.37 ** | 1.39 *** |
| Friendships and Social support (Item 2) T2 | 0.98 | 1.17 * | 1.44 *** | 1.45 *** |
| Friendships and Social support (Item 3) T2 | 0.9 | 1.25 ** | 1.37 *** | 1.36 *** |
| Friendships and Social support (Item 4) T2 | 0.88 | 1.22 ** | 1.31 ** | 1.31 *** |
| Internalizing Symptoms (Item 1) T2 | 0.91 | 1.38 *** | 1.47 *** | 1.39 *** |
| Internalizing Symptoms (Item 2) T2 | 0.97 | 1.44 *** | 1.55 *** | 1.33 *** |
| Internalizing Symptoms (Item 3) T2 | 1.08 | 1.43 *** | 1.56 *** | 1.32 *** |
| Internalizing Symptoms (Item 4) T2 | 0.99 | 1.43 *** | 1.51 *** | 1.27 ** |
| Internalizing Symptoms (Item 5) T2 | 0.96 | 1.46 *** | 1.55 *** | 1.37 *** |
| Internalizing Symptoms (Item 6) T2 | 0.95 | 1.42 *** | 1.5 *** | 1.3 *** |
| Internalizing Symptoms (Item 7) T2 | 0.93 | 1.51 *** | 1.59 *** | 1.34 *** |
| Internalizing Symptoms (Item 8) T2 | 0.97 | 1.46 *** | 1.51 *** | 1.35 *** |
| Internalizing Symptoms (Item 9) T2 | 0.94 | 1.44 *** | 1.51 *** | 1.35 *** |
| Internalizing Symptoms (Item 10) T2 | 0.94 | 1.46 *** | 1.54 *** | 1.37 *** |
| Bullying Victimization (Item 1) T3 | 0.82 ** | 1.28 *** | 1.47 *** | 1.26 ** |
| Bullying Victimization (Item 2) T3 | 0.87 | 1.3 *** | 1.45 *** | 1.24 ** |
| Bullying Victimization (Item 3) T3 | 0.87 * | 1.36 *** | 1.45 *** | 1.23 ** |
| Friendships and Social support (Item 1) T3 | 0.8 ** | 1.31 *** | 1.42 *** | 1.29 ** |
| Friendships and Social support (Item 2) T3 | 0.85 * | 1.41 *** | 1.48 *** | 1.26 ** |
| Friendships and Social support (Item 3) T3 | 0.79 *** | 1.33 *** | 1.43 *** | 1.24 ** |
| Friendships and Social support (Item 4) T3 | 0.8 ** | 1.35 *** | 1.48 *** | 1.26 ** |
| Internalizing Symptoms (Item 1) T3 | 0.91 | 1.43 *** | 1.31 ** | 1.32 *** |
| Internalizing Symptoms (Item 2) T3 | 0.94 | 1.47 *** | 1.35 ** | 1.33 *** |
| Internalizing Symptoms (Item 3) T3 | 0.97 | 1.44 *** | 1.43 *** | 1.32 *** |
| Internalizing Symptoms (Item 4) T3 | 0.89 | 1.52 *** | 1.37 *** | 1.3 *** |
| Internalizing Symptoms (Item 5) T3 | 0.88 | 1.47 *** | 1.37 *** | 1.29 *** |
| Internalizing Symptoms (Item 6) T3 | 0.92 | 1.45 *** | 1.43 *** | 1.25 ** |
| Internalizing Symptoms (Item 7) T3 | 0.86 * | 1.41 *** | 1.31 ** | 1.29 *** |
| Internalizing Symptoms (Item 8) T3 | 0.92 | 1.44 *** | 1.39 *** | 1.28 ** |
| Internalizing Symptoms (Item 9) T3 | 0.91 | 1.48 *** | 1.41 *** | 1.26 ** |
| Internalizing Symptoms (Item 10) T3 | 0.88 | 1.47 *** | 1.43 *** | 1.28 *** |

*Note.* T = Time point;

* p < .05, ** p < .01, *** p < .001

# Appendix C) Item Parceling.

As the confirmatory factor analysis suggested that the original pre-registered measurement models for friendship and social support, and Internalizing symptoms were not appropriate for further RI-CLPM modelling (CFI < .930). Exploratory factor analysis and parallel analysis were used to inform the parcels for the measurement we should use. For friendship and social support, the factor retention methods specifically recommended for short, highly correlated scales consistently supported a one-factor solution. These methods included Comparison Data analysis and Sequential Chi Square Model Tests (Auerswald & Moshagen, 2019). This unidimensional structure was further supported by the Empirical Kaiser criterion, Kaiser-Guttmann criterion, and parallel analysis using PCA-determined eigenvalues. However, a parallel analysis based on EFA-determined eigenvalues suggested two dimensions, suggesting that there might be some degree of correlated residuals. Thus, we included two friendship items into a parcel and two social support items in another to reduce the impact of correlated residuals (Little et al., 2022; Hall et al., 1999).

For internalizing symptoms, unidimensionality was confirmed through Exploratory Graph Analysis (EGA) and previously demonstrated using samples from the same survey (Black et al., 2024). While the scale was unidimensional, a parallel analysis (Table S3) and modification indices from a one-factor Confirmatory Factor Analysis (CFA) suggested correlated residuals among items. To address these correlated residuals while maintaining the unidimensional structure, we created parcels based on the item groupings identified in the parallel analysis.

**Table S3.**

*Exploratory Factor Analysis of Internalizing Symptom Indicators*

|  | Factor1 | Factor2 | Factor3 | Factor4 |  | Factor1 | Factor2 | Factor3 | Factor4 |  | Factor1 | Factor2 | Factor3 | Factor4 |
| --- | --- | --- | --- | --- | --- | --- | --- | --- | --- | --- | --- | --- | --- | --- |
|  | Time 1 | | | |  | Time 2 | | | |  | Time 3 | | | |
| Internalizing Symptoms Item 1 | 0.958 |  |  |  |  | 0.945 |  |  |  |  | 0.976 |  |  |  |
| Internalizing Symptoms Item 2 | 0.640 |  |  |  |  | 0.656 |  |  |  |  | 0.701 |  |  |  |
| Internalizing Symptoms Item 3 | 0.700 |  |  |  |  | 0.658 |  |  |  |  | 0.614 |  |  |  |
| Internalizing Symptoms Item 5 |  | 0.565 |  |  |  |  | 0.638 |  |  |  |  | 0.663 |  |  |
| Internalizing Symptoms Item 6 |  | 1.054 |  |  |  |  | 1.010 |  |  |  |  | 1.038 |  |  |
| Internalizing Symptoms Item 7 |  |  | 0.613 |  |  |  |  | 0.824 |  |  |  |  | 0.652 |  |
| Internalizing Symptoms Item 8 |  |  | 0.898 |  |  |  |  | 0.731 |  |  |  |  | 0.924 |  |
| Internalizing Symptoms Item 10 |  |  |  | 0.985 |  |  |  |  | 0.953 |  |  |  |  | 0.995 |
| Internalizing Symptoms Item 4 |  | 0.341 |  |  |  |  | 0.436 |  |  |  |  | 0.444 |  |  |
| Internalizing Symptoms Item 9 |  |  |  | 0.368 |  |  |  |  | 0.410 |  |  |  |  | 0.418 |

*Note.* A parallel analysis based on EFA-determined eigenvalues was employed.

# Appendix D) Measurement Invariance Results

**Table S4.**

*Longitudinal Measurement Invariance of Bullying Victimization, Friendships, Social Support, and Internalizing Symptoms Across Three Waves*

| Model tested | $\chi^{2}$ | $df$ | $p$ | ${\Delta\chi}^{2}$ | $\Delta df$ | $p$ | RMSEA | RMSEA 90% CI | CFI | $\Delta$CFI | TLI/ NNFI | $\Delta$TLI | SRMR | Pass? |
| --- | --- | --- | --- | --- | --- | --- | --- | --- | --- | --- | --- | --- | --- | --- |
| Null model | 147343.474 | 351 | <.001 |  |  |  |  |  |  |  |  |  |  |  |
|  |  |  |  | Measurement model | | | |  |  |  |  |  |  |  |
| Configural invariance | 3667.199 | 261 | <.001 |  |  |  | .032 | (.031, .033) | .980 |  | .973 |  | .024 | Yes |
| Metric invariance | 3854.922 | 273 | <.001 | 184.61 | 12 | <.001 | .032 | (.031, .033) | .979 | -0.001 | .973 | 0 | .025 | Yes |
| Scalar invariance | 4108.323 | 285 | <.001 | 271.23 | 12 | <.001 | .032 | (.031, .034) | .978 | -0.001 | .973 | 0 | .026 | Yes |
| Residual invariance | 4266.086 | 303 | <.001 | 190.77 | 18 | <.001 | .033 | (.032, .034) | .976 | -0.002 | .973 | 0 | .026 | Yes |
|  |  |  |  | Structure model | | | |  |  |  |  |  |  |  |
| RI-CLPM | 4115.821 | 288 | <.001 | 6.17 | 3 | 0.103 | .032 | (.031, .033) | .978 | 0 | .973 | 0 | .026 | Yes |

*Note.* $\chi^{2}$ = chi-square; df = degrees of freedom; RMSEA = root mean square error of approximation; CFI = Comparative Fit Index; TLI = Tucker-Lewis Index; SRMR = Standardized Root Mean Squared Residual. RI-CLPM fit indices are from the baseline RICLPM without covariates, and the model was constructed using the scalar invariance measurement model. RI-CLPM is compared with the scalar invariance CFA.

**Table S5.**

*Multi-Group Measurement Invariance of Bullying Victimization, Friendships, Social Support, and Internalizing Symptoms Across Gender at Time 1*

| Model tested | $\chi^{2}$ | $df$ | $p$ | ${\Delta\chi}^{2}$ | $\Delta df$ | $p$ | RMSEA | RMSEA 90% CI | CFI | $\Delta$CFI | TLI/ NNFI | $\Delta$TLI | SRMR | Pass? |
| --- | --- | --- | --- | --- | --- | --- | --- | --- | --- | --- | --- | --- | --- | --- |
| Null model | 39960.659 | 72 | <.001 |  |  |  |  |  |  |  |  |  |  |  |
|  |  |  |  | Measurement model | | | |  |  |  |  |  |  |  |
| Configural invariance | 990.447 | 48 | <.001 |  |  |  | .054 | (.051, .057) | .978 |  | .967 |  | .020 | Yes |
| Metric invariance | 1144.624 | 54 | <.001 | 150.55 | 6 | <.001 | .055 | (.052, .058) | .974 | -0.004 | .965 | -0.001 | .026 | Yes |
| Scalar invariance | 2159.836 | 60 | <.001 | 1219.2 | 6 | <.001 | .072 | (.069, .074) | .951 | -0.023 | .941 | -0.024 | .035 | No |
| Residual invariance | 2273.965 | 69 | <.001 | 175.23 | 9 | <.001 | .071 | (.068, .073) | .945 | -0.006 | .943 | 0.002 | .037 | No |

*Note.* $\chi^{2}$ = chi-square; df = degrees of freedom; RMSEA = root mean square error of approximation; CFI = Comparative Fit Index; TLI = Tucker-Lewis Index; SRMR = Standardized Root Mean Squared Residual.

**Table S6.**

*Multi-Group Measurement Invariance of Bullying Victimization, Friendships, Social Support, and Internalizing Symptoms Across Gender at Time 2*

| Model tested | $\chi^{2}$ | $df$ | $p$ | ${\Delta\chi}^{2}$ | $\Delta df$ | $p$ | RMSEA | RMSEA 90% CI | CFI | $\Delta$CFI | TLI/ NNFI | $\Delta$TLI | SRMR | Pass? |
| --- | --- | --- | --- | --- | --- | --- | --- | --- | --- | --- | --- | --- | --- | --- |
| Null model | 38344.747 | 72 | <.001 |  |  |  |  |  |  |  |  |  |  |  |
|  |  |  |  | Measurement model | | | |  |  |  |  |  |  |  |
| Configural invariance | 802.046 | 48 | <.001 |  |  |  | .051 | (.048, .054) | .982 |  | .973 |  | .018 | Yes |
| Metric invariance | 932.250 | 54 | <.001 | 125.87 | 6 | <.001 | .052 | (.049, .055) | .979 | -0.003 | .973 | -0.001 | .024 | Yes |
| Scalar invariance | 2192.201 | 60 | <.001 | 1523.7 | 6 | <.001 | .076 | (.074, .079) | .950 | -0.029 | .940 | -0.032 | .036 | No |
| Residual invariance | 2555.856 | 69 | <.001 | 353.27 | 9 | <.001 | .079 | (.077, .082) | .938 | -0.012 | .935 | -0.005 | .040 | No |

*Note.* $\chi^{2}$ = chi-square; df = degrees of freedom; RMSEA = root mean square error of approximation; CFI = Comparative Fit Index; TLI = Tucker-Lewis Index; SRMR = Standardized Root Mean Squared Residual.

**Table S7.**

*Multi-Group Measurement Invariance of Bullying Victimization, Friendships, Social Support, and Internalizing Symptoms Across Gender at Time 3*

| Model tested | $\chi^{2}$ | $df$ | $p$ | ${\Delta\chi}^{2}$ | $\Delta df$ | $p$ | RMSEA | RMSEA 90% CI | CFI | $\Delta$CFI | TLI/ NNFI | $\Delta$TLI | SRMR | Pass? |
| --- | --- | --- | --- | --- | --- | --- | --- | --- | --- | --- | --- | --- | --- | --- |
| Null model | 37623.488 | 72 | <.001 |  |  |  |  |  |  |  |  |  |  |  |
|  |  |  |  | Measurement model | | | |  |  |  |  |  |  |  |
| Configural invariance | 704.682 | 48 | <.001 |  |  |  | .050 | (.047, .053) | .984 |  | .976 |  | .017 | Yes |
| Metric invariance | 856.530 | 54 | <.001 | 142.6 | 6 | <.001 | .053 | (.049, .056) | .981 | -0.004 | .974 | -0.002 | .024 | Yes |
| Scalar invariance | 2492.569 | 60 | <.001 | 2171.3 | 6 | <.001 | .085 | (.082, .088) | .943 | -0.038 | .931 | -0.043 | .038 | No |
| Residual invariance | 2784.099 | 69 | <.001 | 321.21 | 9 | <.001 | .088 | (.085, .091) | .931 | -0.012 | .928 | -0.004 | .043 | No |

*Note.* $\chi^{2}$ = chi-square; df = degrees of freedom RMSEA = root mean square error of approximation; CFI = Comparative Fit Index; TLI = Tucker-Lewis Index; SRMR = Standardized Root Mean Squared Residual.

**Table S8.**

*Omnibus Measurement Invariance of Bullying Victimization, Friendships, Social Support, and Internalizing Symptoms Across Groups and Time Points*

| Model tested | $\chi^{2}$ | $df$ | $p$ | ${\Delta\chi}^{2}$ | $\Delta df$ | $p$ | RMSEA | RMSEA 90% CI | CFI | $\Delta$CFI | TLI/ NNFI | $\Delta$TLI | SRMR | Pass? |
| --- | --- | --- | --- | --- | --- | --- | --- | --- | --- | --- | --- | --- | --- | --- |
| Null model | 147181.868 | 702 | <.001 |  |  |  |  |  |  |  |  |  |  |  |
|  |  |  |  | Measurement model | | | |  |  |  |  |  |  |  |
| Configural invariance | 3140.514 | 522 | <.001 |  |  |  | .028 | (.027, .029) | .984 |  | .978 |  | .022 |  |
| Metric invariance | 3802.555 | 552 | <.001 | 576.48 | 30 | < .001 | .030 | (.029, .032) | .980 | -0.004 | .974 | -0.004 | .029 | Yes |
| Longitudinal Scalar and group metric invariance | 4143.630 | 576 | <.001 | 380.06 | 24 | < .001 | .031 | (.030, .032) | .978 | -0.002 | .973 | -0.001 | .029 | Yes |
| Longitudinal Residual and group metric invariance | 4509.013 | 612 | <.001 | 330.38 | 36 | < .001 | .032 | (.031, .033) | .975 | -0.003 | .971 | -0.002 | .030 | Yes |
|  |  |  |  | Structure model | | | |  |  |  |  |  |  |  |
| MG RICLPM | 4154.873 | 582 | <.001 | 9.8591 | 6 | 0.131 | .031 | (.030, .032) | .978 | 0 | .973 | 0 | .029 | Yes |

*Note.* $\chi^{2}$ = chi-square; df = degrees of freedom RMSEA = root mean square error of approximation; CFI = Comparative Fit Index; TLI = Tucker-Lewis Index; SRMR = Standardized Root Mean Squared Residual. The gender group measurement invariance suggested that the assumption of scalar invariance across gender groups was rejected, so longitudinal scalar and group metric measurement invariance was tested here. RI-CLPM fit indices are from the baseline RICLPM without covariates, and the model was constructed using the longitudinal scalar and group metric invariance measurement model and compared with the longitudinal scalar and group metric invariance CFA.

# Appendix E) Baseline RI-CLPM

**Table S9.**

*Latent Variance–Covariance Matrix and Latent Means*

| Construct | BVT1 | BVT2 | BVT3 | FST1 | FST2 | FST3 | IST1 | IST2 | IST3 |
| --- | --- | --- | --- | --- | --- | --- | --- | --- | --- |
| BVT1 | 1.00 |  |  |  |  |  |  |  |  |
| BVT2 | 0.58 | 1.00 |  |  |  |  |  |  |  |
| BVT3 | 0.40 | 0.53 | 1.00 |  |  |  |  |  |  |
| FST1 | -0.47 | -0.33 | -0.23 | 1.00 |  |  |  |  |  |
| FST2 | -0.32 | -0.48 | -0.30 | 0.51 | 1.00 |  |  |  |  |
| FST3 | -0.27 | -0.33 | -0.46 | 0.40 | 0.52 | 1.00 |  |  |  |
| IST1 | 0.55 | 0.38 | 0.27 | -0.59 | -0.43 | -0.34 | 1.00 |  |  |
| IST2 | 0.40 | 0.53 | 0.34 | -0.40 | -0.57 | -0.39 | 0.65 | 1.00 |  |
| IST3 | 0.31 | 0.37 | 0.51 | -0.30 | -0.38 | -0.53 | 0.52 | 0.59 | 1.00 |
| Means | 1.38 | 1.40 | 1.35 | 3.85 | 3.78 | 3.80 | 0.66 | 0.66 | 0.63 |

*Note.* Results are based on the effects-coded method of identification with the scalar invariant across time. BV = Bullying Victimization. FS = Friendship and social support. IS = Internalizing symptoms. T1, 2, 3 = Time 1, 2, 3.

**Table S10.**

*Results of the Baseline Partial Stationary RI-CLPM*

|  |  | Paths | | | | |
| --- | --- | --- | --- | --- | --- | --- |
| Path | Time | *b* | *p* | *CI* | *beta* | *Hypotheses* |
|  |  | Cross-lagged Pathways | | | | |
| BV → FS | T1 → T2 | -0.03 | .4 | [-0.09, 0.04] | -0.03 | H1A partially supported |
| BV → FS | T2 → T3 | -0.06* | .04 | [-0.11, -0.00] | -0.06 | H1A partially supported |
| BV → IS | T1 → T2 | 0.11*** | < .001 | [0.06, 0.16] | 0.1 | H1B supported |
| BV → IS | T2 → T3 | 0.11*** | < .001 | [0.06, 0.16] | 0.14 | H1B supported |
| FS → BV | T1 → T2 | -0.02 | .4 | [-0.07, 0.03] | -0.02 | H2A rejected |
| FS → BV | T2 → T3 | -0.02 | .4 | [-0.07, 0.03] | -0.02 | H2A rejected |
| FS → IS | T1 → T2 | -0.06* | .01 | [-0.11, -0.01] | -0.06 | H2B supported |
| FS → IS | T2 → T3 | -0.06* | .01 | [-0.11, -0.01] | -0.06 | H2B supported |
| IS → BV | T1 → T2 | 0.05 | .07 | [-0.00, 0.10] | 0.05 | H3A rejected |
| IS → BV | T2 → T3 | 0.05 | .07 | [-0.00, 0.10] | 0.05 | H3A rejected |
| IS → FS | T1 → T2 | -0.17*** | < .001 | [-0.24, -0.09] | -0.16 | H3B supported |
| IS → FS | T2 → T3 | -0.10*** | < .001 | [-0.16, -0.04] | -0.1 | H3B supported |
|  |  | Autoregressive Effects | | | | |
| BV → BV | T1 → T2 | 0.36*** | < .001 | [0.27, 0.45] | 0.33 | H4A supported |
| BV → BV | T2 → T3 | 0.28*** | < .001 | [0.21, 0.36] | 0.29 | H4A supported |
| FS → FS | T1 → T2 | 0.16*** | < .001 | [0.08, 0.24] | 0.15 | H4B supported |
| FS → FS | T2 → T3 | 0.21*** | < .001 | [0.14, 0.28] | 0.21 | H4B supported |
| IS → IS | T1 → T2 | 0.26*** | < .001 | [0.19, 0.33] | 0.24 | H4C supported |
| IS → IS | T2 → T3 | 0.16*** | < .001 | [0.09, 0.22] | 0.16 | H4C supported |

*Notes.* $\chi^{2}$ = 7357.008, df = 390, RMSEA = 0.035 (.035, . 036), CFI =0 964, TLI = 0.957, SRMR = 0.031. N = 26458.

*p < .05, **p <.01, ***p <.001

# Appendix F) Structure Variance by Gender

In Table S11, we provided more tests regarding gender differences, extending the analysis in the main text. We found that fixing cross-lagged effects to be equal worsened model fits in four out of eight settings. In these four settings, concurrent associations (CA) were fixed to be equal across genders, indicating a pattern: when CA was fixed to be equal, cross-lagged (CL) effects could not be fixed to be equal. Further testing (Table S12) revealed that CA could not be fixed to be equal across genders in any of the settings. These results suggested that when a single-group RICLPM was used with CA constrained to be equal, CL paths could not be fixed to be equal.

Model.Base corresponds to Model 1 in the main text, while Model.CA.AR.CL corresponds to Model 2. Model.PS represents the multigroup model suggested by the partial stationary single-group CLPM (see Table S10), applying the same temporal constraints for both genders. A comparison of Model.CA.AR.CL against Model.Base (${\Delta\chi}^{2}$ = 51.426, $\Delta df$= 27, p = 0.003) and Model.PS.CA.AR.CL against Model.PS (${\Delta\chi}^{2}$ = 45.984, $\Delta df$= 21, p = 0.001) suggested within-parts structure variance by gender. Therefore, we should not assume structural invariance by gender.

**Table S11.**

*Comparison of Models Constraining Cross-Lagged Effects Across Genders with Models Without Cross-Lagged Constraints in Different Settings*

| Models | $\chi^{2}$ | $df$ | p | CFI | TLI | RMSEA | SRMR | Compared with | ${\Delta\chi}^{2}$ | $\Delta df$ | $p$ |
| --- | --- | --- | --- | --- | --- | --- | --- | --- | --- | --- | --- |
| Model.Base | 5036.80 | 726 | 0 | 0.975 | 0.970 | 0.030 | 0.030 |  |  |  |  |
| Model.CL | 5048.01 | 738 | 0 | 0.975 | 0.970 | 0.030 | 0.031 | Model.Base | 17.295 | 12 | 0.14 |
| Model.PS | 5028.30 | 738 | 0 | 0.975 | 0.970 | 0.030 | 0.030 |  |  |  |  |
| Model.PS.CL | 5035.22 | 746 | 0 | 0.975 | 0.971 | 0.029 | 0.031 | Model.PS | 11.934 | 8 | 0.15 |
| Model.PS.CA | 5051.49 | 745 | 0 | 0.974 | 0.970 | 0.029 | 0.031 |  |  |  |  |
| Model.PS.CA.CL | 5062.46 | 753 | 0 | 0.974 | 0.971 | 0.029 | 0.031 | Model.PS.CA | 18.27 | 8 | 0.02* |
| Model.PS.CA.AR | 5040.44 | 751 | 0 | 0.975 | 0.971 | 0.029 | 0.031 |  |  |  |  |
| Model.PS.CA.AR.CL | 5052.18 | 759 | 0 | 0.974 | 0.971 | 0.029 | 0.031 | Model.PS.CA.AR | 18.537 | 8 | 0.02* |
| Model.PS.AR | 5022.44 | 744 | 0 | 0.975 | 0.971 | 0.029 | 0.031 |  |  |  |  |
| Model.PS.AR.CL | 5029.07 | 752 | 0 | 0.975 | 0.971 | 0.029 | 0.031 | Model.PS.AR | 11.418 | 8 | 0.18 |
| Model.CA | 5051.63 | 735 | 0 | 0.975 | 0.970 | 0.030 | 0.031 |  |  |  |  |
| Model.CA.CL | 5068.97 | 747 | 0 | 0.974 | 0.970 | 0.030 | 0.031 | Model.CA | 26.985 | 12 | 0.008** |
| Model.CA.AR | 5043.95 | 741 | 0 | 0.975 | 0.970 | 0.030 | 0.031 |  |  |  |  |
| Model.CA.AR.CL | 5058.33 | 753 | 0 | 0.974 | 0.971 | 0.029 | 0.031 | Model.CA.AR | 18.537 | 8 | 0.02* |
| Model.AR | 5034.12 | 732 | 0 | 0.975 | 0.970 | 0.030 | 0.031 |  |  |  |  |
| Model.AR.CL | 5041.52 | 744 | 0 | 0.975 | 0.970 | 0.029 | 0.031 | Model.AR | 14.983 | 12 | 0.24 |

*Note.* Model.Base represents the baseline multigroup RI-CLPM, where all paths between within-components are freely estimated. CL = fixed cross-lagged effects to be equal across genders; AR = fixed autoregressive effects to be equal across genders; CA = fixed concurrent associations to be equal across genders. Model.CA indicates the concurrent associations (CA) being fixed. PS = constrained congeneric paths with similar effect sizes are set to be equal based on the partial stationarity suggested in the single-group RI-CLPM.

*p < .05, **p <.01, ***p <.001

**Table S12.**

*Comparison of Models Constraining Concurrent Associations to Be Equal Across Genders with Models Not Constraining Concurrent Associations in Different Settings*

| Models | Compared with | ${\Delta\chi}^{2}$ | $\Delta df$ | $p$ |
| --- | --- | --- | --- | --- |
| Model.CA.AR | Model.AR | 20.383 | 9 | .02* |
| Model.CA.AR.CL | Model.AR.CL | 28.383 | 9 | < .001*** |
| Model.CA | Model.Base | 23.38 | 9 | 0.005** |
| Model.CA.CL | Model.CL | 31.035 | 9 | < .001*** |
| Model.PS.CA | Model.PS | 26.953 | 7 | < .001*** |
| Model.PS.CA.AR | Model.PS.AR | 23.196 | 7 | 0.002** |
| Model.PS.CA.AR.CL | Model.PS.AR.CL | 28.713 | 7 | < .001*** |
| Model.PS.CA.CL | Model.PS.CL | 31.608 | 7 | < .001*** |

*Note.* Model.Base indicates the baseline multigroup RI-CLPM, where all paths between within-components are freely estimated. CL = fixed cross-lagged effects to be equal across genders; AR = fixed autoregressive effects to be equal across genders; CA = fixed concurrent associations to be equal across genders. PS = constrained congeneric paths with similar effect sizes to be equal, based on the partial stationarity suggested in the single-group RI-CLPM.

*p < .05, **p <.01, ***p <.001

# Appendix G) Sensitivity Analysis: Multiple Imputation

**Table S13.**

*Results of the Multigroup RI-CLPM Using Multiple Imputation for Participants with at Least Two Survey Waves*

|  |  | *Girl* | | |  | *Boy* | | |
| --- | --- | --- | --- | --- | --- | --- | --- | --- |
| Path | Time | *b* | *p* | *Hypotheses* |  | *b* | *p* | *Hypotheses* |
|  |  | Cross-lagged Pathways | | | | | | |
| BV → FS | T1 → T2 | -0.08* | 0.03 | H1A supported |  | 0.02 | 0.54 | H1A rejected |
| BV → FS | T2 → T3 | -0.08* | 0.03 | H1A supported |  | -0.04 | 0.26 | H1A rejected |
| BV → IS | T1 → T2 | 0.04 | 0.34 | H1B partially supported |  | 0.17*** | < .001 | H1B supported |
| BV → IS | T2 → T3 | 0.12** | 0.005 | H1B partially supported |  | 0.09** | 0.01 | H1B supported |
| FS → BV | T1 → T2 | -0.05 | 0.12 | H2A rejected |  | 0 | 0.91 | H2A rejected |
| FS → BV | T2 → T3 | -0.05 | 0.12 | H2A rejected |  | 0 | 0.91 | H2A rejected |
| FS → IS | T1 → T2 | -0.06 | 0.16 | H2B partially supported |  | -0.03 | 0.26 | H2B rejected |
| FS → IS | T2 → T3 | -0.10* | 0.01 | H2B partially supported |  | -0.03 | 0.26 | H2B rejected |
| IS → BV | T1 → T2 | -0.01 | 0.77 | H3A rejected |  | 0.09** | 0.005 | H3A supported |
| IS → BV | T2 → T3 | -0.01 | 0.77 | H3A rejected |  | 0.09** | 0.005 | H3A supported |
| IS → FS | T1 → T2 | -0.14** | 0.004 | H3B supported |  | -0.15*** | < .001 | H3B supported |
| IS → FS | T2 → T3 | -0.08* | 0.04 | H3B supported |  | -0.11*** | < .001 | H3B supported |
|  |  | Autoregressive Effects | | | | | | |
| BV → BV | T1 → T2 | 0.38*** | < .001 | H4A supported |  | 0.31*** | < .001 | H4A supported |
| BV → BV | T2 → T3 | 0.38*** | < .001 | H4A supported |  | 0.24*** | < .001 | H4A supported |
| FS → FS | T1 → T2 | 0.17** | 0.003 | H4B supported |  | 0.13* | 0.012 | H4B supported |
| FS → FS | T2 → T3 | 0.22*** | < .001 | H4B supported |  | 0.20*** | < .001 | H4B supported |
| IS → IS | T1 → T2 | 0.33*** | < .001 | H4C supported |  | 0.18*** | < .001 | H4C supported |
| IS → IS | T2 → T3 | 0.17*** | < .001 | H4C supported |  | 0.18*** | < .001 | H4C supported |

*Notes.* *p < .05, **p <.01, ***p <.001

**Table S14.**

*Results of the Multigroup RI-CLPM Using Multiple Imputation for Participants with at Least One Survey Waves*

|  |  | *Girl* | | |  | *Boy* | | |
| --- | --- | --- | --- | --- | --- | --- | --- | --- |
| Path | Time | *b* | *p* | *Hypotheses* |  | *b* | *p* | *Hypotheses* |
|  |  | Cross-lagged Pathways | | | | | | |
| BV → FS | T1 → T2 | -0.08* | 0.03 | H1A supported |  | 0.01 | 0.81 | H1A rejected |
| BV → FS | T2 → T3 | -0.08* | 0.03 | H1A supported |  | -0.02 | 0.50 | H1A rejected |
| BV → IS | T1 → T2 | 0.05 | 0.25 | H1B partially supported |  | 0.17*** | < .001 | H1B supported |
| BV → IS | T2 → T3 | 0.13*** | < .001 | H1B partially supported |  | 0.07* | 0.047 | H1B supported |
| FS → BV | T1 → T2 | -0.05 | 0.18 | H2A rejected |  | 0 | 0.88 | H2A rejected |
| FS → BV | T2 → T3 | -0.05 | 0.18 | H2A rejected |  | 0 | 0.88 | H2A rejected |
| FS → IS | T1 → T2 | -0.06 | 0.12 | H2B partially supported |  | -0.03 | 0.25 | H2B rejected |
| FS → IS | T2 → T3 | -0.10* | 0.02 | H2B partially supported |  | -0.03 | 0.25 | H2B rejected |
| IS → BV | T1 → T2 | -0.01 | 0.87 | H3A rejected |  | 0.10** | 0.002 | H3A supported |
| IS → BV | T2 → T3 | -0.01 | 0.87 | H3A rejected |  | 0.10** | 0.002 | H3A supported |
| IS → FS | T1 → T2 | -0.15** | 0.001 | H3B partially supported |  | -0.17*** | < .001 | H3B supported |
| IS → FS | T2 → T3 | -0.08* | 0.04 | H3B partially supported |  | -0.14*** | < .001 | H3B supported |
|  |  | Autoregressive Effects | | | | | | |
| BV → BV | T1 → T2 | 0.39*** | < .001 | H4A supported |  | 0.31*** | < .001 | H4A supported |
| BV → BV | T2 → T3 | 0.39*** | < .001 | H4A supported |  | 0.23*** | < .001 | H4A supported |
| FS → FS | T1 → T2 | 0.17*** | < .001 | H4B supported |  | 0.13* | 0.015 | H4B supported |
| FS → FS | T2 → T3 | 0.22*** | < .001 | H4B supported |  | 0.19*** | < .001 | H4B supported |
| IS → IS | T1 → T2 | 0.33*** | < .001 | H4C supported |  | 0.20*** | < .001 | H4C supported |
| IS → IS | T2 → T3 | 0.17*** | < .001 | H4C supported |  | 0.20*** | < .001 | H4C supported |

*Notes.* *p < .05, **p <.01, ***p <.001

# Appendix H) Sensitivity Analysis: Measurement Model and Other

**Table S15.**

*Results of the Multigroup RI-CLPM Using Longitudinal Residual and Group Metric Invariance Measurement Model*

|  |  | Girls | | | | |  | Boys | | | | |
| --- | --- | --- | --- | --- | --- | --- | --- | --- | --- | --- | --- | --- |
| Path | Time | *b* | *p* | *CI* | *beta* | *Hypotheses* |  | *b* | *p* | *CI* | *beta* | *Hypotheses* |
|  |  | Cross-lagged Pathways | | | | | | | | | | |
| BV → FS | T1 → T2 | -0.09* | 0.02 | [-0.16, -0.01] | -0.08 | H1A supported |  | 0.05 | 0.19 | [-0.03, 0.14] | 0.05 | H1A rejected |
| BV → FS | T2 → T3 | -0.09* | 0.02 | [-0.16, -0.01] | -0.09 | H1A supported |  | -0.03 | 0.39 | [-0.11, 0.04] | -0.03 | H1A rejected |
| BV → IS | T1 → T2 | 0.05 | 0.23 | [-0.03, 0.14] | 0.05 | H1B partially supported |  | 0.10** | 0.003 | [0.03, 0.17] | 0.1 | H1B supported |
| BV → IS | T2 → T3 | 0.14*** | < .001 | [0.06, 0.22] | 0.14 | H1B partially supported |  | 0.10** | 0.003 | [0.03, 0.17] | 0.1 | H1B supported |
| FS → BV | T1 → T2 | -0.06 | 0.14 | [-0.13, 0.02] | -0.05 | H2A rejected |  | 0 | 0.95 | [-0.06, 0.06] | 0 | H2A rejected |
| FS → BV | T2 → T3 | -0.06 | 0.14 | [-0.13, 0.02] | -0.05 | H2A rejected |  | 0 | 0.95 | [-0.06, 0.06] | 0 | H2A rejected |
| FS → IS | T1 → T2 | -0.06 | 0.12 | [-0.14, 0.02] | -0.06 | H2B partially supported |  | -0.04 | 0.17 | [-0.11, 0.02] | -0.04 | H2B rejected |
| FS → IS | T2 → T3 | -0.10* | 0.01 | [-0.18, -0.02] | -0.1 | H2B partially supported |  | -0.04 | 0.17 | [-0.11, 0.02] | -0.04 | H2B rejected |
| IS → BV | T1 → T2 | 0 | 0.91 | [-0.08, 0.07] | 0 | H3A rejected |  | 0.09* | 0.02 | [0.02, 0.16] | 0.08 | H3A supported |
| IS → BV | T2 → T3 | 0 | 0.91 | [-0.08, 0.07] | 0 | H3A rejected |  | 0.09* | 0.02 | [0.02, 0.16] | 0.09 | H3A supported |
| IS → FS | T1 → T2 | -0.16** | 0.002 | [-0.26, -0.06] | -0.15 | H3B partially supported |  | -0.16*** | < .001 | [-0.26, -0.07] | -0.16 | H3B supported |
| IS → FS | T2 → T3 | -0.08 | 0.06 | [-0.17, 0.00] | -0.09 | H3B partially supported |  | -0.13** | 0.002 | [-0.21, -0.05] | -0.13 | H3B supported |
|  |  | Autoregressive Effects | | | | | | | | | | |
| BV → BV | T1 → T2 | 0.39*** | < .001 | [0.27, 0.50] | 0.36 | H4A supported |  | 0.25*** | < .001 | [0.17, 0.33] | 0.24 | H4A supported |
| BV → BV | T2 → T3 | 0.39*** | < .001 | [0.27, 0.50] | 0.38 | H4A supported |  | 0.25*** | < .001 | [0.17, 0.33] | 0.25 | H4A supported |
| FS → FS | T1 → T2 | 0.17** | 0.002 | [0.06, 0.28] | 0.16 | H4B supported |  | 0.16** | 0.003 | [0.05, 0.26] | 0.15 | H4B supported |
| FS → FS | T2 → T3 | 0.22*** | < .001 | [0.12, 0.32] | 0.22 | H4B supported |  | 0.20*** | < .001 | [0.10, 0.29] | 0.19 | H4B supported |
| IS → IS | T1 → T2 | 0.32*** | < .001 | [0.21, 0.43] | 0.3 | H4C supported |  | 0.23*** | < .001 | [0.13, 0.33] | 0.22 | H4C supported |
| IS → IS | T2 → T3 | 0.15** | 0.001 | [0.06, 0.25] | 0.16 | H4C supported |  | 0.15*** | < .001 | [0.06, 0.23] | 0.15 | H4C supported |

*Notes.* The same constraints were used as for the model reported in the main text. $\chi^{2}$ = 5388.969, df = 776, RMSEA =0.030 (.029, .031), CFI =0.972, TLI = 0.969, SRMR = 0.032. N = 26458.

*p < .05, **p <.01, ***p <.001

**Table S16.**

*Results of the Multigroup RI-CLPM Using Original Indicators with Adding Covarying Paths*

|  |  | Girls | | | | |  | Boys | | | | |
| --- | --- | --- | --- | --- | --- | --- | --- | --- | --- | --- | --- | --- |
| Path | Time | *b* | *p* | *CI* | *beta* | *Hypotheses* |  | *b* | *p* | *CI* | *beta* | *Hypotheses* |
|  |  | Cross-lagged Pathways | | | | | | | | | | |
| BV → FS | T1 → T2 | -0.08* | 0.03 | [-0.16, -0.01] | -0.08 | H1A supported |  | 0.07 | 0.1 | [-0.01, 0.16] | 0.07 | H1A rejected |
| BV → FS | T2 → T3 | -0.08* | 0.03 | [-0.16, -0.01] | -0.08 | H1A supported |  | -0.03 | 0.42 | [-0.10, 0.04] | -0.03 | H1A rejected |
| BV → IS | T1 → T2 | 0.03 | 0.46 | [-0.06, 0.12] | 0.03 | H1B partially supported |  | 0.08* | 0.02 | [0.02, 0.15] | 0.08 | H1B supported |
| BV → IS | T2 → T3 | 0.13** | 0.002 | [0.04, 0.21] | 0.13 | H1B partially supported |  | 0.08* | 0.02 | [0.02, 0.15] | 0.08 | H1B supported |
| FS → BV | T1 → T2 | -0.06 | 0.14 | [-0.13, 0.02] | -0.05 | H2A rejected |  | 0 | 0.93 | [-0.07, 0.06] | 0 | H2A rejected |
| FS → BV | T2 → T3 | -0.06 | 0.14 | [-0.13, 0.02] | -0.06 | H2A rejected |  | 0 | 0.93 | [-0.07, 0.06] | 0 | H2A rejected |
| FS → IS | T1 → T2 | -0.05 | 0.2 | [-0.14, 0.03] | -0.05 | H2B partially supported |  | -0.03 | 0.39 | [-0.09, 0.04] | -0.03 | H2B rejected |
| FS → IS | T2 → T3 | -0.10* | 0.01 | [-0.18, -0.02] | -0.1 | H2B partially supported |  | -0.03 | 0.39 | [-0.09, 0.04] | -0.03 | H2B rejected |
| IS → BV | T1 → T2 | -0.01 | 0.82 | [-0.09, 0.07] | -0.01 | H3A rejected |  | 0.09* | 0.02 | [0.01, 0.16] | 0.08 | H3A supported |
| IS → BV | T2 → T3 | -0.01 | 0.82 | [-0.09, 0.07] | -0.01 | H3A rejected |  | 0.09* | 0.02 | [0.01, 0.16] | 0.09 | H3A supported |
| IS → FS | T1 → T2 | -0.16** | 0.002 | [-0.27, -0.06] | -0.15 | H3B partially supported |  | -0.19*** | < .001 | [-0.30, -0.09] | -0.19 | H3B supported |
| IS → FS | T2 → T3 | -0.08 | 0.09 | [-0.17, 0.01] | -0.08 | H3B partially supported |  | -0.12** | 0.003 | [-0.20, -0.04] | -0.12 | H3B supported |
|  |  | Autoregressive Effects | | | | | | | | | | |
| BV → BV | T1 → T2 | 0.37*** | < .001 | [0.25, 0.49] | 0.34 | H4A supported |  | 0.24*** | < .001 | [0.15, 0.32] | 0.23 | H4A supported |
| BV → BV | T2 → T3 | 0.37*** | < .001 | [0.25, 0.49] | 0.37 | H4A supported |  | 0.24*** | < .001 | [0.15, 0.32] | 0.24 | H4A supported |
| FS → FS | T1 → T2 | 0.16** | 0.004 | [0.05, 0.27] | 0.15 | H4B supported |  | 0.13* | 0.01 | [0.03, 0.24] | 0.13 | H4B supported |
| FS → FS | T2 → T3 | 0.23*** | < .001 | [0.14, 0.33] | 0.23 | H4B supported |  | 0.19*** | < .001 | [0.10, 0.28] | 0.19 | H4B supported |
| IS → IS | T1 → T2 | 0.35*** | < .001 | [0.23, 0.46] | 0.32 | H4C supported |  | 0.27*** | < .001 | [0.17, 0.37] | 0.26 | H4C supported |
| IS → IS | T2 → T3 | 0.18*** | < .001 | [0.08, 0.27] | 0.18 | H4C supported |  | 0.17*** | < .001 | [0.09, 0.26] | 0.18 | H4C supported |

*Notes.* The same constraints were applied as in the model reported in the main text. The measurement model was fitted with the original indicators, incorporating the covariance between indicators within a dimension proposed by the EFA. $\chi^{2}$ =14696.755, df =2648, RMSEA =0.027(.027, .028), CFI =0.962, TLI = 0.959, SRMR = 0.037. N = 26458.

*p < .05, **p <.01, ***p <.001

**Table S17**

*Results of the Multigroup RI-CLPM Using Manifest Indicators*

|  |  | Girls | | | | |  | Boys | | | | |
| --- | --- | --- | --- | --- | --- | --- | --- | --- | --- | --- | --- | --- |
| Path | Time | *b* | *p* | *CI* | *beta* | *Hypotheses* |  | *b* | *p* | *CI* | *beta* | *Hypotheses* |
|  |  | Cross-lagged Pathways | | | | | | | | | | |
| BV → FS | T1 → T2 | -0.07** | 0.005 | [-0.11, -0.02] | -0.06 | H1A supported |  | 0 | 0.89 | [-0.05, 0.06] | 0 | H1A partially supported |
| BV → FS | T2 → T3 | -0.07** | 0.005 | [-0.11, -0.02] | -0.07 | H1A supported |  | -0.07* | 0.01 | [-0.12, -0.01] | -0.07 | H1A partially supported |
| BV → IS | T1 → T2 | 0.04 | 0.12 | [-0.01, 0.09] | 0.04 | H1B partially supported |  | 0.09*** | < .001 | [0.04, 0.14] | 0.09 | H1B supported |
| BV → IS | T2 → T3 | 0.10*** | < .001 | [0.05, 0.15] | 0.1 | H1B partially supported |  | 0.09*** | < .001 | [0.04, 0.14] | 0.09 | H1B supported |
| FS → BV | T1 → T2 | -0.05* | 0.03 | [-0.09, -0.00] | -0.05 | H2A supported |  | -0.03 | 0.23 | [-0.07, 0.02] | -0.03 | H2A rejected |
| FS → BV | T2 → T3 | -0.05* | 0.03 | [-0.09, -0.00] | -0.05 | H2A supported |  | -0.03 | 0.23 | [-0.07, 0.02] | -0.03 | H2A rejected |
| FS → IS | T1 → T2 | -0.06* | 0.02 | [-0.11, -0.01] | -0.06 | H2B supported |  | -0.03 | 0.16 | [-0.08, 0.01] | -0.03 | H2B rejected |
| FS → IS | T2 → T3 | -0.06* | 0.01 | [-0.11, -0.01] | -0.06 | H2B supported |  | -0.03 | 0.16 | [-0.08, 0.01] | -0.03 | H2B rejected |
| IS → BV | T1 → T2 | 0.03 | 0.23 | [-0.02, 0.07] | 0.03 | H3A rejected |  | 0.08** | 0.003 | [0.03, 0.13] | 0.07 | H3A supported |
| IS → BV | T2 → T3 | 0.03 | 0.23 | [-0.02, 0.07] | 0.03 | H3A rejected |  | 0.08** | 0.003 | [0.03, 0.13] | 0.08 | H3A supported |
| IS → FS | T1 → T2 | -0.13*** | < .001 | [-0.19, -0.07] | -0.12 | H3B supported |  | -0.11*** | < .001 | [-0.17, -0.05] | -0.11 | H3B supported |
| IS → FS | T2 → T3 | -0.06* | 0.03 | [-0.12, -0.01] | -0.06 | H3B supported |  | -0.09** | 0.001 | [-0.15, -0.04] | -0.09 | H3B supported |
|  |  | Autoregressive Effects | | | | | | | | | | |
| BV → BV | T1 → T2 | 0.26*** | < .001 | [0.19, 0.33] | 0.25 | H4A supported |  | 0.21*** | < .001 | [0.15, 0.27] | 0.2 | H4A supported |
| BV → BV | T2 → T3 | 0.26*** | < .001 | [0.19, 0.33] | 0.26 | H4A supported |  | 0.21*** | < .001 | [0.15, 0.27] | 0.21 | H4A supported |
| FS → FS | T1 → T2 | 0.14*** | < .001 | [0.08, 0.20] | 0.14 | H4B supported |  | 0.12*** | < .001 | [0.05, 0.19] | 0.12 | H4B supported |
| FS → FS | T2 → T3 | 0.19*** | < .001 | [0.13, 0.25] | 0.19 | H4B supported |  | 0.16*** | < .001 | [0.10, 0.22] | 0.16 | H4B supported |
| IS → IS | T1 → T2 | 0.27*** | < .001 | [0.20, 0.34] | 0.25 | H4C supported |  | 0.20*** | < .001 | [0.13, 0.27] | 0.19 | H4C supported |
| IS → IS | T2 → T3 | 0.16*** | < .001 | [0.10, 0.23] | 0.17 | H4C supported |  | 0.14*** | < .001 | [0.08, 0.21] | 0.14 | H4C supported |

*Notes*. The same constraints were applied as in the model reported in the main text. The model was fitted with using the manifest measures, by averaging the indicators for each construct. $\chi^{2}$ = 85.834, df =56, RMSEA = 0.009(.003, .014), CFI = 0.999, TLI = 0.998, SRMR = 0.009. N = 26458.

*p < .05, **p <.01, ***p <.001

**Table S18**

*Results of the Multigroup RI-CLPM Using an Alternative Identification Approach*

|  |  | Girls | | | | |  | Boys | | | | |
| --- | --- | --- | --- | --- | --- | --- | --- | --- | --- | --- | --- | --- |
| Path | Time | *b* | *p* | *CI* | *beta* | *Hypotheses* |  | *b* | *p* | *CI* | *beta* | *Hypotheses* |
|  |  | Cross-lagged Pathways | | | | | | | | | | |
| BV → FS | T1 → T2 | -0.13* | 0.03 | [-0.26, -0.01] | -0.08 | H1A supported |  | 0.06 | 0.38 | [-0.08, 0.20] | 0.04 | H1A rejected |
| BV → FS | T2 → T3 | -0.13* | 0.03 | [-0.26, -0.01] | -0.09 | H1A supported |  | -0.05 | 0.42 | [-0.16, 0.06] | -0.03 | H1A rejected |
| BV → IS | T1 → T2 | 0.04 | 0.29 | [-0.03, 0.11] | 0.05 | H1B partially supported |  | 0.09** | 0.001 | [0.04, 0.15] | 0.12 | H1B supported |
| BV → IS | T2 → T3 | 0.13*** | < .001 | [0.06, 0.20] | 0.16 | H1B partially supported |  | 0.06* | 0.03 | [0.01, 0.12] | 0.09 | H1B supported |
| FS → BV | T1 → T2 | -0.03 | 0.21 | [-0.08, 0.02] | -0.05 | H2A rejected |  | -0.01 | 0.83 | [-0.05, 0.04] | -0.01 | H2A rejected |
| FS → BV | T2 → T3 | -0.03 | 0.21 | [-0.08, 0.02] | -0.05 | H2A rejected |  | -0.01 | 0.83 | [-0.05, 0.04] | -0.01 | H2A rejected |
| FS → IS | T1 → T2 | -0.03 | 0.09 | [-0.07, 0.01] | -0.07 | H2B partially supported |  | -0.02 | 0.17 | [-0.05, 0.01] | -0.04 | H2B rejected |
| FS → IS | T2 → T3 | -0.05* | 0.01 | [-0.09, -0.01] | -0.1 | H2B partially supported |  | -0.02 | 0.17 | [-0.05, 0.01] | -0.04 | H2B rejected |
| IS → BV | T1 → T2 | 0.08 | 0.21 | [-0.04, 0.20] | 0.06 | H3A rejected |  | 0.16* | 0.01 | [0.04, 0.28] | 0.09 | H3A supported |
| IS → BV | T2 → T3 | -0.03 | 0.53 | [-0.14, 0.07] | -0.03 | H3A rejected |  | 0.16* | 0.01 | [0.04, 0.28] | 0.1 | H3A supported |
| IS → FS | T1 → T2 | -0.35*** | < .001 | [-0.56, -0.15] | -0.17 | H3B partially supported |  | -0.45*** | < .001 | [-0.68, -0.22] | -0.18 | H3B supported |
| IS → FS | T2 → T3 | -0.15 | 0.09 | [-0.33, 0.02] | -0.08 | H3B partially supported |  | -0.30** | 0.001 | [-0.48, -0.12] | -0.13 | H3B supported |
|  |  | Autoregressive Effects | | | | | | | | | | |
| BV → BV | T1 → T2 | 0.36*** | < .001 | [0.24, 0.48] | 0.34 | H4A supported |  | 0.32*** | < .001 | [0.21, 0.43] | 0.27 | H4A supported |
| BV → BV | T2 → T3 | 0.36*** | < .001 | [0.24, 0.48] | 0.37 | H4A supported |  | 0.25*** | < .001 | [0.15, 0.35] | 0.24 | H4A supported |
| FS → FS | T1 → T2 | 0.17** | 0.004 | [0.05, 0.28] | 0.16 | H4B supported |  | 0.16** | 0.007 | [0.04, 0.28] | 0.15 | H4B supported |
| FS → FS | T2 → T3 | 0.23*** | < .001 | [0.13, 0.33] | 0.23 | H4B supported |  | 0.21*** | < .001 | [0.12, 0.31] | 0.21 | H4B supported |
| IS → IS | T1 → T2 | 0.32*** | < .001 | [0.22, 0.42] | 0.31 | H4C supported |  | 0.26*** | < .001 | [0.15, 0.36] | 0.22 | H4C supported |
| IS → IS | T2 → T3 | 0.15** | 0.005 | [0.04, 0.25] | 0.14 | H4C supported |  | 0.19*** | < .001 | [0.09, 0.29] | 0.18 | H4C supported |

*Notes.* The same constraints were applied as in the model reported in the main text. $\chi^{2}$ = 5029.052, df = 735, RMSEA = 0.030(.029, .031), CFI = 0.975, TLI = 0.970, SRMR = 0.031. N = 26458.

*p < .05, **p <.01, ***p <.001

**Table S19**

*Results of the Nonstationary Multigroup RI-CLPM*

|  |  | Girls | | | | |  | Boys | | | | |
| --- | --- | --- | --- | --- | --- | --- | --- | --- | --- | --- | --- | --- |
| Path | Time | *b* | *p* | *CI* | *beta* | *Hypotheses* |  | *b* | *p* | *CI* | *beta* | *Hypotheses* |
|  |  | Cross-lagged Pathways | | | | | | | | | | |
| BV → FS | T1 → T2 | -0.1 | 0.06 | [-0.20, 0.01] | -0.09 | H1A partially supported |  | 0.04 | 0.38 | [-0.05, 0.13] | 0.04 | H1A rejected |
| BV → FS | T2 → T3 | -0.10* | 0.02 | [-0.18, -0.01] | -0.1 | H1A partially supported |  | -0.03 | 0.42 | [-0.11, 0.04] | -0.03 | H1A rejected |
| BV → IS | T1 → T2 | 0.07 | 0.18 | [-0.03, 0.16] | 0.06 | H1B partially supported |  | 0.13** | 0.003 | [0.05, 0.22] | 0.12 | H1B supported |
| BV → IS | T2 → T3 | 0.15*** | < .001 | [0.07, 0.24] | 0.16 | H1B partially supported |  | 0.08* | 0.04 | [0.00, 0.16] | 0.08 | H1B supported |
| FS → BV | T1 → T2 | -0.07 | 0.15 | [-0.17, 0.03] | -0.06 | H2A rejected |  | -0.02 | 0.66 | [-0.10, 0.06] | -0.02 | H2A rejected |
| FS → BV | T2 → T3 | -0.05 | 0.33 | [-0.14, 0.05] | -0.05 | H2A rejected |  | 0 | 0.94 | [-0.07, 0.08] | 0 | H2A rejected |
| FS → IS | T1 → T2 | -0.08 | 0.07 | [-0.16, 0.01] | -0.07 | H2B partially supported |  | -0.06 | 0.16 | [-0.14, 0.02] | -0.06 | H2B rejected |
| FS → IS | T2 → T3 | -0.10* | 0.01 | [-0.18, -0.02] | -0.1 | H2B partially supported |  | -0.03 | 0.39 | [-0.11, 0.04] | -0.03 | H2B rejected |
| IS → BV | T1 → T2 | 0.01 | 0.88 | [-0.10, 0.12] | 0.01 | H3A rejected |  | 0.08 | 0.12 | [-0.02, 0.17] | 0.07 | H3A partially supported |
| IS → BV | T2 → T3 | -0.02 | 0.7 | [-0.11, 0.07] | -0.02 | H3A rejected |  | 0.10* | 0.02 | [0.01, 0.18] | 0.1 | H3A partially supported |
| IS → FS | T1 → T2 | -0.17** | 0.003 | [-0.28, -0.05] | -0.15 | H3B partially supported |  | -0.18** | 0.001 | [-0.28, -0.07] | -0.17 | H3B supported |
| IS → FS | T2 → T3 | -0.07 | 0.12 | [-0.16, 0.02] | -0.07 | H3B partially supported |  | -0.13** | 0.001 | [-0.21, -0.05] | -0.13 | H3B supported |
|  |  | Autoregressive Effects | | | | | | | | | | |
| BV → BV | T1 → T2 | 0.44*** | < .001 | [0.27, 0.61] | 0.4 | H4A supported |  | 0.30*** | < .001 | [0.18, 0.41] | 0.28 | H4A supported |
| BV → BV | T2 → T3 | 0.37*** | < .001 | [0.23, 0.51] | 0.38 | H4A supported |  | 0.24*** | < .001 | [0.14, 0.33] | 0.24 | H4A supported |
| FS → FS | T1 → T2 | 0.18** | 0.003 | [0.06, 0.29] | 0.17 | H4B supported |  | 0.16** | 0.006 | [0.04, 0.27] | 0.15 | H4B supported |
| FS → FS | T2 → T3 | 0.23*** | < .001 | [0.13, 0.34] | 0.23 | H4B supported |  | 0.20*** | < .001 | [0.11, 0.30] | 0.2 | H4B supported |
| IS → IS | T1 → T2 | 0.32*** | < .001 | [0.21, 0.43] | 0.3 | H4C supported |  | 0.22*** | < .001 | [0.11, 0.33] | 0.21 | H4C supported |
| IS → IS | T2 → T3 | 0.14** | 0.005 | [0.04, 0.24] | 0.14 | H4C supported |  | 0.17*** | < .001 | [0.08, 0.26] | 0.17 | H4C supported |

*Notes.* $\chi^{2}$ = 5036.796, df = 726, RMSEA = 0.030(.029, .031), CFI = 0.975, TLI = 0.970, SRMR = 0.030. N = 26458

*p < .05, **p <.01, ***p <.001

**Table S20**

*Results with SEs adjusted by clustering school IDs.*

|  |  | Girls | | | | |  | Boys | | | | |
| --- | --- | --- | --- | --- | --- | --- | --- | --- | --- | --- | --- | --- |
| Path | Time | *b* | *p* | *CI* | *beta* | *Hypotheses* |  | *b* | *p* | *CI* | *beta* | *Hypotheses* |
|  |  | Cross-lagged Pathways | | | | | | | | | | |
| BV → FS | T1 → T2 | -0.09* | 0.05 | [-0.19, -0.00] | -0.09 | H1A supported |  | 0.04 | 0.39 | [-0.05, 0.14] | 0.04 | H1A rejected |
| BV → FS | T2 → T3 | -0.09* | 0.05 | [-0.19, -0.00] | -0.1 | H1A supported |  | -0.02 | 0.57 | [-0.11, 0.06] | -0.03 | H1A rejected |
| BV → IS | T1 → T2 | 0.06 | 0.2 | [-0.03, 0.16] | 0.06 | H1B partially supported |  | 0.13** | 0.005 | [0.04, 0.22] | 0.12 | H1B supported |
| BV → IS | T2 → T3 | 0.15** | 0.002 | [0.06, 0.24] | 0.15 | H1B partially supported |  | 0.08* | 0.04 | [0.00, 0.15] | 0.08 | H1B supported |
| FS → BV | T1 → T2 | -0.08 | 0.1 | [-0.18, 0.02] | -0.07 | H2A rejected |  | 0 | 0.97 | [-0.08, 0.07] | 0 | H2A rejected |
| FS → BV | T2 → T3 | -0.04 | 0.4 | [-0.13, 0.05] | -0.04 | H2A rejected |  | 0 | 0.97 | [-0.08, 0.07] | 0 | H2A rejected |
| FS → IS | T1 → T2 | -0.09* | 0.01 | [-0.16, -0.02] | -0.08 | H2B supported |  | -0.04 | 0.19 | [-0.09, 0.02] | -0.03 | H2B rejected |
| FS → IS | T2 → T3 | -0.09* | 0.01 | [-0.16, -0.02] | -0.09 | H2B supported |  | -0.04 | 0.19 | [-0.09, 0.02] | -0.04 | H2B rejected |
| IS → BV | T1 → T2 | -0.01 | 0.86 | [-0.08, 0.07] | -0.01 | H3A rejected |  | 0.09* | 0.04 | [0.00, 0.17] | 0.08 | H3A supported |
| IS → BV | T2 → T3 | -0.01 | 0.86 | [-0.08, 0.07] | -0.01 | H3A rejected |  | 0.09* | 0.04 | [0.00, 0.17] | 0.09 | H3A supported |
| IS → FS | T1 → T2 | -0.16** | 0.003 | [-0.26, -0.05] | -0.15 | H3B partially supported |  | -0.18** | 0.002 | [-0.29, -0.07] | -0.17 | H3B supported |
| IS → FS | T2 → T3 | -0.08 | 0.1 | [-0.16, 0.01] | -0.08 | H3B partially supported |  | -0.12* | 0.01 | [-0.22, -0.03] | -0.12 | H3B supported |
|  |  | Autoregressive Effects | | | | | | | | | | |
| BV → BV | T1 → T2 | 0.44*** | < .001 | [0.29, 0.59] | 0.4 | H4A supported |  | 0.29*** | < .001 | [0.15, 0.42] | 0.27 | H4A supported |
| BV → BV | T2 → T3 | 0.36*** | < .001 | [0.22, 0.50] | 0.37 | H4A supported |  | 0.23*** | < .001 | [0.14, 0.32] | 0.23 | H4A supported |
| FS → FS | T1 → T2 | 0.19** | 0.001 | [0.07, 0.30] | 0.18 | H4B supported |  | 0.15* | 0.01 | [0.04, 0.27] | 0.15 | H4B supported |
| FS → FS | T2 → T3 | 0.23*** | < .001 | [0.14, 0.32] | 0.23 | H4B supported |  | 0.21*** | < .001 | [0.10, 0.31] | 0.21 | H4B supported |
| IS → IS | T1 → T2 | 0.31*** | < .001 | [0.20, 0.42] | 0.29 | H4C supported |  | 0.23*** | < .001 | [0.13, 0.34] | 0.22 | H4C supported |
| IS → IS | T2 → T3 | 0.15** | 0.003 | [0.05, 0.25] | 0.15 | H4C supported |  | 0.16*** | < .001 | [0.08, 0.25] | 0.17 | H4C supported |

*Notes.* The constraints were obtained using the same procedure as for the model reported in the main text.

*p < .05, **p <.01, ***p <.001

**Table S21.**

*Results of the Multigroup RI-CLPM for Participants in At Least Two Survey Waves*

|  |  | Girls | | | | |  | Boys | | | | |
| --- | --- | --- | --- | --- | --- | --- | --- | --- | --- | --- | --- | --- |
| Path | Time | *b* | *p* | *CI* | *beta* | *Hypotheses* |  | *b* | *p* | *CI* | *beta* | *Hypotheses* |
|  |  | Cross-lagged Pathways | | | | | | | | | | |
| BV → FS | T1 → T2 | -0.08* | 0.03 | [-0.16, -0.01] | -0.08 | H1A supported |  | 0.03 | 0.45 | [-0.05, 0.12] | 0.03 | H1A rejected |
| BV → FS | T2 → T3 | -0.08* | 0.03 | [-0.16, -0.01] | -0.08 | H1A supported |  | -0.02 | 0.57 | [-0.09, 0.05] | -0.02 | H1A rejected |
| BV → IS | T1 → T2 | 0.05 | 0.3 | [-0.04, 0.14] | 0.04 | H1B partially supported |  | 0.14** | 0.002 | [0.05, 0.23] | 0.13 | H1B partially supported |
| BV → IS | T2 → T3 | 0.14*** | < .001 | [0.06, 0.23] | 0.15 | H1B partially supported |  | 0.06 | 0.09 | [-0.01, 0.13] | 0.06 | H1B partially supported |
| FS → BV | T1 → T2 | -0.05 | 0.18 | [-0.13, 0.02] | -0.05 | H2A rejected |  | 0 | 0.91 | [-0.07, 0.06] | 0 | H2A rejected |
| FS → BV | T2 → T3 | -0.05 | 0.18 | [-0.13, 0.02] | -0.05 | H2A rejected |  | 0 | 0.91 | [-0.07, 0.06] | 0 | H2A rejected |
| FS → IS | T1 → T2 | -0.07 | 0.1 | [-0.15, 0.01] | -0.06 | H2B partially supported |  | -0.03 | 0.32 | [-0.09, 0.03] | -0.03 | H2B rejected |
| FS → IS | T2 → T3 | -0.10** | 0.009 | [-0.18, -0.03] | -0.1 | H2B partially supported |  | -0.03 | 0.32 | [-0.09, 0.03] | -0.03 | H2B rejected |
| IS → BV | T1 → T2 | 0.04 | 0.44 | [-0.06, 0.13] | 0.03 | H3A rejected |  | 0.06 | 0.19 | [-0.03, 0.14] | 0.05 | H3A partially supported |
| IS → BV | T2 → T3 | -0.02 | 0.57 | [-0.11, 0.06] | -0.02 | H3A rejected |  | 0.10* | 0.01 | [0.02, 0.18] | 0.1 | H3A partially supported |
| IS → FS | T1 → T2 | -0.17** | 0.001 | [-0.27, -0.06] | -0.16 | H3B partially supported |  | -0.13*** | < .001 | [-0.20, -0.06] | -0.13 | H3B supported |
| IS → FS | T2 → T3 | -0.08 | 0.07 | [-0.17, 0.01] | -0.08 | H3B partially supported |  | -0.13*** | < .001 | [-0.20, -0.06] | -0.13 | H3B supported |
|  |  | Autoregressive Effects | | | | | | | | | | |
| BV → BV | T1 → T2 | 0.36*** | < .001 | [0.24, 0.49] | 0.34 | H4A supported |  | 0.29*** | < .001 | [0.18, 0.40] | 0.28 | H4A supported |
| BV → BV | T2 → T3 | 0.36*** | < .001 | [0.24, 0.49] | 0.36 | H4A supported |  | 0.23*** | < .001 | [0.13, 0.32] | 0.23 | H4A supported |
| FS → FS | T1 → T2 | 0.17** | 0.003 | [0.06, 0.28] | 0.16 | H4B supported |  | 0.15** | 0.004 | [0.05, 0.25] | 0.15 | H4B supported |
| FS → FS | T2 → T3 | 0.23*** | < .001 | [0.13, 0.33] | 0.23 | H4B supported |  | 0.21*** | < .001 | [0.12, 0.30] | 0.2 | H4B supported |
| IS → IS | T1 → T2 | 0.33*** | < .001 | [0.22, 0.44] | 0.31 | H4C supported |  | 0.19*** | < .001 | [0.11, 0.26] | 0.18 | H4C supported |
| IS → IS | T2 → T3 | 0.15** | 0.002 | [0.06, 0.25] | 0.16 | H4C supported |  | 0.19*** | < .001 | [0.11, 0.26] | 0.19 | H4C supported |

*Notes.* The constraints were set using the same procedure as for the model reported in the main text. $\chi^{2}$ = 4310.194, df =737, RMSEA =0.030(.029, .031), CFI =0.974, TLI = 0.970, SRMR = 0.031. N = 17978.

*p < .05, **p <.01, ***p <.001

# Appendix I) Measurement Invariance Syntax

*library*('semTools')
*library*('lavaan')

mod.indicator <-'
# BV Latent
T1LBV =~ T1BVP1 + T1BVP2 + T1BVP3
T2LBV =~ T2BVP1 + T2BVP2 + T2BVP3
T3LBV =~ T3BVP1 + T3BVP2 + T3BVP3

# FS Latent
T1LFS =~ T1FSP1 + T1FSP2
T2LFS =~ T2FSP1 + T2FSP2
T3LFS =~ T3FSP1 + T3FSP2

# IS Latent
T1LIS =~ T1ISP1 + T1ISP2 + T1ISP3 + T1ISP4
T2LIS =~ T2ISP1 + T2ISP2 + T2ISP3 + T2ISP4
T3LIS =~ T3ISP1 + T3ISP2 + T3ISP3 + T3ISP4
'

longFacNames <- *list*(BV = *c*("T1LBV","T2LBV","T3LBV"),
 FS = *c*("T1LFS","T2LFS","T3LFS"),
 IS = *c*("T1LIS","T2LIS","T3LIS"))

time.config.syntax <- *measEq.syntax*(configural.model = mod.indicator,
 data = data,
 ID.fac = "effects.code",
 longFacNames = longFacNames)

time.metric.syntax <- *measEq.syntax*(configural.model = mod.indicator,
 data = data,
 ID.fac = "effects.code",
 longFacNames = longFacNames,
 long.equal = *c*("loadings"))

time.scalar.syntax <- *measEq.syntax*(configural.model = mod.indicator,
 data = data,
 ID.fac = "effects.code",
 longFacNames = longFacNames,
 long.equal = *c*("loadings","intercepts"))

time.resid.syntax <- *measEq.syntax*(configural.model = mod.indicator,
 data = data,
 ID.fac = "effects.code",
 longFacNames = longFacNames,
 long.equal = *c*("loadings","intercepts","residuals"))

auxvars <- *c*("GENDER","ETHNICITY","SEN","FSMEVER")

time.config.fit.aux <- *auxiliary*(*as.character*(time.config.syntax), fun = "cfa", aux = auxvars, data=data, estimator="MLR", missing="FIML")
time.metric.fit.aux <- *auxiliary*(*as.character*(time.metric.syntax), fun = "cfa", aux = auxvars, data=data, estimator="MLR", missing="FIML")
time.scalar.fit.aux <- *auxiliary*(*as.character*(time.scalar.syntax), fun = "cfa", aux = auxvars, data=data, estimator="MLR", missing="FIML")
time.resid.fit.aux <- *auxiliary*(*as.character*(time.resid.syntax), fun = "cfa", aux = auxvars, data=data, estimator="MLR", missing="FIML")

time.metric.aux.compare <- semTools::*compareFit*(time.config.fit.aux, time.metric.fit.aux, nested = TRUE)
time.scalar.aux.compare <- semTools::*compareFit*(time.metric.fit.aux, time.scalar.fit.aux, nested = TRUE)
time.resid.aux.compare <- semTools::*compareFit*(time.scalar.fit.aux, time.resid.fit.aux, nested = TRUE)

# Appendix J) RI-CLPM Syntax

*library*('semTools')
*library*('lavaan')

MG.Model.RICLPM <- “

## LOADINGS:

T1LBV =~ c(NA, NA)*T1BVP1 + c(lambda.1_1, lambda.1_1)*T1BVP1

T1LBV =~ c(NA, NA)*T1BVP2 + c(lambda.2_1, lambda.2_1)*T1BVP2

T1LBV =~ c(NA, NA)*T1BVP3 + c(lambda.3_1, lambda.3_1)*T1BVP3

T2LBV =~ c(NA, NA)*T2BVP1 + c(lambda.1_1, lambda.1_1)*T2BVP1

T2LBV =~ c(NA, NA)*T2BVP2 + c(lambda.2_1, lambda.2_1)*T2BVP2

T2LBV =~ c(NA, NA)*T2BVP3 + c(lambda.3_1, lambda.3_1)*T2BVP3

T3LBV =~ c(NA, NA)*T3BVP1 + c(lambda.1_1, lambda.1_1)*T3BVP1

T3LBV =~ c(NA, NA)*T3BVP2 + c(lambda.2_1, lambda.2_1)*T3BVP2

T3LBV =~ c(NA, NA)*T3BVP3 + c(lambda.3_1, lambda.3_1)*T3BVP3

T1LFS =~ c(NA, NA)*T1FSP1 + c(lambda.10_4, lambda.10_4)*T1FSP1

T1LFS =~ c(NA, NA)*T1FSP2 + c(lambda.11_4, lambda.11_4)*T1FSP2

T2LFS =~ c(NA, NA)*T2FSP1 + c(lambda.10_4, lambda.10_4)*T2FSP1

T2LFS =~ c(NA, NA)*T2FSP2 + c(lambda.11_4, lambda.11_4)*T2FSP2

T3LFS =~ c(NA, NA)*T3FSP1 + c(lambda.10_4, lambda.10_4)*T3FSP1

T3LFS =~ c(NA, NA)*T3FSP2 + c(lambda.11_4, lambda.11_4)*T3FSP2

T1LIS =~ c(NA, NA)*T1ISP1 + c(lambda.16_7, lambda.16_7)*T1ISP1

T1LIS =~ c(NA, NA)*T1ISP2 + c(lambda.17_7, lambda.17_7)*T1ISP2

T1LIS =~ c(NA, NA)*T1ISP3 + c(lambda.18_7, lambda.18_7)*T1ISP3

T1LIS =~ c(NA, NA)*T1ISP4 + c(lambda.19_7, lambda.19_7)*T1ISP4

T2LIS =~ c(NA, NA)*T2ISP1 + c(lambda.16_7, lambda.16_7)*T2ISP1

T2LIS =~ c(NA, NA)*T2ISP2 + c(lambda.17_7, lambda.17_7)*T2ISP2

T2LIS =~ c(NA, NA)*T2ISP3 + c(lambda.18_7, lambda.18_7)*T2ISP3

T2LIS =~ c(NA, NA)*T2ISP4 + c(lambda.19_7, lambda.19_7)*T2ISP4

T3LIS =~ c(NA, NA)*T3ISP1 + c(lambda.16_7, lambda.16_7)*T3ISP1

T3LIS =~ c(NA, NA)*T3ISP2 + c(lambda.17_7, lambda.17_7)*T3ISP2

T3LIS =~ c(NA, NA)*T3ISP3 + c(lambda.18_7, lambda.18_7)*T3ISP3

T3LIS =~ c(NA, NA)*T3ISP4 + c(lambda.19_7, lambda.19_7)*T3ISP4

## INTERCEPTS:

T1BVP1 ~ c(NA, NA)*1 + c(nu.1.g1, nu.1.g2)*1

T1BVP2 ~ c(NA, NA)*1 + c(nu.2.g1, nu.2.g2)*1

T1BVP3 ~ c(NA, NA)*1 + c(nu.3.g1, nu.3.g2)*1

T2BVP1 ~ c(NA, NA)*1 + c(nu.1.g1, nu.1.g2)*1

T2BVP2 ~ c(NA, NA)*1 + c(nu.2.g1, nu.2.g2)*1

T2BVP3 ~ c(NA, NA)*1 + c(nu.3.g1, nu.3.g2)*1

T3BVP1 ~ c(NA, NA)*1 + c(nu.1.g1, nu.1.g2)*1

T3BVP2 ~ c(NA, NA)*1 + c(nu.2.g1, nu.2.g2)*1

T3BVP3 ~ c(NA, NA)*1 + c(nu.3.g1, nu.3.g2)*1

T1FSP1 ~ c(NA, NA)*1 + c(nu.10.g1, nu.10.g2)*1

T1FSP2 ~ c(NA, NA)*1 + c(nu.11.g1, nu.11.g2)*1

T2FSP1 ~ c(NA, NA)*1 + c(nu.10.g1, nu.10.g2)*1

T2FSP2 ~ c(NA, NA)*1 + c(nu.11.g1, nu.11.g2)*1

T3FSP1 ~ c(NA, NA)*1 + c(nu.10.g1, nu.10.g2)*1

T3FSP2 ~ c(NA, NA)*1 + c(nu.11.g1, nu.11.g2)*1

T1ISP1 ~ c(NA, NA)*1 + c(nu.16.g1, nu.16.g2)*1

T1ISP2 ~ c(NA, NA)*1 + c(nu.17.g1, nu.17.g2)*1

T1ISP3 ~ c(NA, NA)*1 + c(nu.18.g1, nu.18.g2)*1

T1ISP4 ~ c(NA, NA)*1 + c(nu.19.g1, nu.19.g2)*1

T2ISP1 ~ c(NA, NA)*1 + c(nu.16.g1, nu.16.g2)*1

T2ISP2 ~ c(NA, NA)*1 + c(nu.17.g1, nu.17.g2)*1

T2ISP3 ~ c(NA, NA)*1 + c(nu.18.g1, nu.18.g2)*1

T2ISP4 ~ c(NA, NA)*1 + c(nu.19.g1, nu.19.g2)*1

T3ISP1 ~ c(NA, NA)*1 + c(nu.16.g1, nu.16.g2)*1

T3ISP2 ~ c(NA, NA)*1 + c(nu.17.g1, nu.17.g2)*1

T3ISP3 ~ c(NA, NA)*1 + c(nu.18.g1, nu.18.g2)*1

T3ISP4 ~ c(NA, NA)*1 + c(nu.19.g1, nu.19.g2)*1

## UNIQUE-FACTOR VARIANCES:

T1BVP1 ~~ c(NA, NA)*T1BVP1 + c(theta.1_1.g1, theta.1_1.g2)*T1BVP1

T1BVP2 ~~ c(NA, NA)*T1BVP2 + c(theta.2_2.g1, theta.2_2.g2)*T1BVP2

T1BVP3 ~~ c(NA, NA)*T1BVP3 + c(theta.3_3.g1, theta.3_3.g2)*T1BVP3

T2BVP1 ~~ c(NA, NA)*T2BVP1 + c(theta.4_4.g1, theta.4_4.g2)*T2BVP1

T2BVP2 ~~ c(NA, NA)*T2BVP2 + c(theta.5_5.g1, theta.5_5.g2)*T2BVP2

T2BVP3 ~~ c(NA, NA)*T2BVP3 + c(theta.6_6.g1, theta.6_6.g2)*T2BVP3

T3BVP1 ~~ c(NA, NA)*T3BVP1 + c(theta.7_7.g1, theta.7_7.g2)*T3BVP1

T3BVP2 ~~ c(NA, NA)*T3BVP2 + c(theta.8_8.g1, theta.8_8.g2)*T3BVP2

T3BVP3 ~~ c(NA, NA)*T3BVP3 + c(theta.9_9.g1, theta.9_9.g2)*T3BVP3

T1FSP1 ~~ c(NA, NA)*T1FSP1 + c(theta.10_10.g1, theta.10_10.g2)*T1FSP1

T1FSP2 ~~ c(NA, NA)*T1FSP2 + c(theta.11_11.g1, theta.11_11.g2)*T1FSP2

T2FSP1 ~~ c(NA, NA)*T2FSP1 + c(theta.12_12.g1, theta.12_12.g2)*T2FSP1

T2FSP2 ~~ c(NA, NA)*T2FSP2 + c(theta.13_13.g1, theta.13_13.g2)*T2FSP2

T3FSP1 ~~ c(NA, NA)*T3FSP1 + c(theta.14_14.g1, theta.14_14.g2)*T3FSP1

T3FSP2 ~~ c(NA, NA)*T3FSP2 + c(theta.15_15.g1, theta.15_15.g2)*T3FSP2

T1ISP1 ~~ c(NA, NA)*T1ISP1 + c(theta.16_16.g1, theta.16_16.g2)*T1ISP1

T1ISP2 ~~ c(NA, NA)*T1ISP2 + c(theta.17_17.g1, theta.17_17.g2)*T1ISP2

T1ISP3 ~~ c(NA, NA)*T1ISP3 + c(theta.18_18.g1, theta.18_18.g2)*T1ISP3

T1ISP4 ~~ c(NA, NA)*T1ISP4 + c(theta.19_19.g1, theta.19_19.g2)*T1ISP4

T2ISP1 ~~ c(NA, NA)*T2ISP1 + c(theta.20_20.g1, theta.20_20.g2)*T2ISP1

T2ISP2 ~~ c(NA, NA)*T2ISP2 + c(theta.21_21.g1, theta.21_21.g2)*T2ISP2

T2ISP3 ~~ c(NA, NA)*T2ISP3 + c(theta.22_22.g1, theta.22_22.g2)*T2ISP3

T2ISP4 ~~ c(NA, NA)*T2ISP4 + c(theta.23_23.g1, theta.23_23.g2)*T2ISP4

T3ISP1 ~~ c(NA, NA)*T3ISP1 + c(theta.24_24.g1, theta.24_24.g2)*T3ISP1

T3ISP2 ~~ c(NA, NA)*T3ISP2 + c(theta.25_25.g1, theta.25_25.g2)*T3ISP2

T3ISP3 ~~ c(NA, NA)*T3ISP3 + c(theta.26_26.g1, theta.26_26.g2)*T3ISP3

T3ISP4 ~~ c(NA, NA)*T3ISP4 + c(theta.27_27.g1, theta.27_27.g2)*T3ISP4

## UNIQUE-FACTOR COVARIANCES:

T1BVP1 ~~ c(NA, NA)*T2BVP1 + c(theta.4_1.g1, theta.4_1.g2)*T2BVP1

T1BVP1 ~~ c(NA, NA)*T3BVP1 + c(theta.7_1.g1, theta.7_1.g2)*T3BVP1

T1BVP2 ~~ c(NA, NA)*T2BVP2 + c(theta.5_2.g1, theta.5_2.g2)*T2BVP2

T1BVP2 ~~ c(NA, NA)*T3BVP2 + c(theta.8_2.g1, theta.8_2.g2)*T3BVP2

T1BVP3 ~~ c(NA, NA)*T2BVP3 + c(theta.6_3.g1, theta.6_3.g2)*T2BVP3

T1BVP3 ~~ c(NA, NA)*T3BVP3 + c(theta.9_3.g1, theta.9_3.g2)*T3BVP3

T2BVP1 ~~ c(NA, NA)*T3BVP1 + c(theta.7_4.g1, theta.7_4.g2)*T3BVP1

T2BVP2 ~~ c(NA, NA)*T3BVP2 + c(theta.8_5.g1, theta.8_5.g2)*T3BVP2

T2BVP3 ~~ c(NA, NA)*T3BVP3 + c(theta.9_6.g1, theta.9_6.g2)*T3BVP3

T1FSP1 ~~ c(NA, NA)*T2FSP1 + c(theta.12_10.g1, theta.12_10.g2)*T2FSP1

T1FSP1 ~~ c(NA, NA)*T3FSP1 + c(theta.14_10.g1, theta.14_10.g2)*T3FSP1

T1FSP2 ~~ c(NA, NA)*T2FSP2 + c(theta.13_11.g1, theta.13_11.g2)*T2FSP2

T1FSP2 ~~ c(NA, NA)*T3FSP2 + c(theta.15_11.g1, theta.15_11.g2)*T3FSP2

T2FSP1 ~~ c(NA, NA)*T3FSP1 + c(theta.14_12.g1, theta.14_12.g2)*T3FSP1

T2FSP2 ~~ c(NA, NA)*T3FSP2 + c(theta.15_13.g1, theta.15_13.g2)*T3FSP2

T1ISP1 ~~ c(NA, NA)*T2ISP1 + c(theta.20_16.g1, theta.20_16.g2)*T2ISP1

T1ISP1 ~~ c(NA, NA)*T3ISP1 + c(theta.24_16.g1, theta.24_16.g2)*T3ISP1

T1ISP2 ~~ c(NA, NA)*T2ISP2 + c(theta.21_17.g1, theta.21_17.g2)*T2ISP2

T1ISP2 ~~ c(NA, NA)*T3ISP2 + c(theta.25_17.g1, theta.25_17.g2)*T3ISP2

T1ISP3 ~~ c(NA, NA)*T2ISP3 + c(theta.22_18.g1, theta.22_18.g2)*T2ISP3

T1ISP3 ~~ c(NA, NA)*T3ISP3 + c(theta.26_18.g1, theta.26_18.g2)*T3ISP3

T1ISP4 ~~ c(NA, NA)*T2ISP4 + c(theta.23_19.g1, theta.23_19.g2)*T2ISP4

T1ISP4 ~~ c(NA, NA)*T3ISP4 + c(theta.27_19.g1, theta.27_19.g2)*T3ISP4

T2ISP1 ~~ c(NA, NA)*T3ISP1 + c(theta.24_20.g1, theta.24_20.g2)*T3ISP1

T2ISP2 ~~ c(NA, NA)*T3ISP2 + c(theta.25_21.g1, theta.25_21.g2)*T3ISP2

T2ISP3 ~~ c(NA, NA)*T3ISP3 + c(theta.26_22.g1, theta.26_22.g2)*T3ISP3

T2ISP4 ~~ c(NA, NA)*T3ISP4 + c(theta.27_23.g1, theta.27_23.g2)*T3ISP4

## LATENT MEANS/INTERCEPTS:

T1LBV ~ c(NA, NA)*1 + c(alpha.1.g1, alpha.1.g2)*1

T2LBV ~ c(NA, NA)*1 + c(alpha.2.g1, alpha.2.g2)*1

T3LBV ~ c(NA, NA)*1 + c(alpha.3.g1, alpha.3.g2)*1

T1LFS ~ c(NA, NA)*1 + c(alpha.4.g1, alpha.4.g2)*1

T2LFS ~ c(NA, NA)*1 + c(alpha.5.g1, alpha.5.g2)*1

T3LFS ~ c(NA, NA)*1 + c(alpha.6.g1, alpha.6.g2)*1

T1LIS ~ c(NA, NA)*1 + c(alpha.7.g1, alpha.7.g2)*1

T2LIS ~ c(NA, NA)*1 + c(alpha.8.g1, alpha.8.g2)*1

T3LIS ~ c(NA, NA)*1 + c(alpha.9.g1, alpha.9.g2)*1

## MODEL CONSTRAINTS:

lambda.1_1 == 3 - lambda.2_1 - lambda.3_1

nu.1.g1 == 0 - nu.2.g1 - nu.3.g1

nu.1.g2 == 0 - nu.2.g2 - nu.3.g2

nu.1.g1 == 0 - nu.2.g1 - nu.3.g1

nu.1.g2 == 0 - nu.2.g2 - nu.3.g2

nu.1.g1 == 0 - nu.2.g1 - nu.3.g1

nu.1.g2 == 0 - nu.2.g2 - nu.3.g2

lambda.10_4 == 2 - lambda.11_4

nu.10.g1 == 0 - nu.11.g1

nu.10.g2 == 0 - nu.11.g2

nu.10.g1 == 0 - nu.11.g1

nu.10.g2 == 0 - nu.11.g2

nu.10.g1 == 0 - nu.11.g1

nu.10.g2 == 0 - nu.11.g2

lambda.16_7 == 4 - lambda.17_7 - lambda.18_7 - lambda.19_7

nu.16.g1 == 0 - nu.17.g1 - nu.18.g1 - nu.19.g1

nu.16.g2 == 0 - nu.17.g2 - nu.18.g2 - nu.19.g2

nu.16.g1 == 0 - nu.17.g1 - nu.18.g1 - nu.19.g1

nu.16.g2 == 0 - nu.17.g2 - nu.18.g2 - nu.19.g2

nu.16.g1 == 0 - nu.17.g1 - nu.18.g1 - nu.19.g1

nu.16.g2 == 0 - nu.17.g2 - nu.18.g2 - nu.19.g2

#VARIANCES of latent variables

T1LBV ~~ c(0, 0)*T1LBV

T2LBV ~~ c(0, 0)*T2LBV

T3LBV ~~ c(0, 0)*T3LBV

T1LFS ~~ c(0, 0)*T1LFS

T2LFS ~~ c(0, 0)*T2LFS

T3LFS ~~ c(0, 0)*T3LFS

T1LIS ~~ c(0, 0)*T1LIS

T2LIS ~~ c(0, 0)*T2LIS

T3LIS ~~ c(0, 0)*T3LIS

################

# BETWEEN PART #

################

# define random intercepts

RIBV =~ c(beta.1_19.g1, beta.1_19.g2)*T1LBV + start(0.1, 0.1)*T1LBV

RIBV =~ c(beta.1_19.g1, beta.1_19.g2)*T2LBV + start(0.1, 0.1)*T2LBV

RIBV =~ c(beta.1_19.g1, beta.1_19.g2)*T3LBV + start(0.1, 0.1)*T3LBV

RIFS =~ c(beta.4_20.g1, beta.4_20.g2)*T1LFS + start(0.1, 0.1)*T1LFS

RIFS =~ c(beta.4_20.g1, beta.4_20.g2)*T2LFS + start(0.1, 0.1)*T2LFS

RIFS =~ c(beta.4_20.g1, beta.4_20.g2)*T3LFS + start(0.1, 0.1)*T3LFS

RIIS =~ c(beta.7_21.g1, beta.7_21.g2)*T1LIS + start(0.1, 0.1)*T1LIS

RIIS =~ c(beta.7_21.g1, beta.7_21.g2)*T2LIS + start(0.1, 0.1)*T2LIS

RIIS =~ c(beta.7_21.g1, beta.7_21.g2)*T3LIS + start(0.1, 0.1)*T3LIS

RIBV ~~ c(1, 1)*RIBV

RIFS ~~ c(1, 1)*RIFS

RIIS ~~ c(1, 1)*RIIS

RIBV ~~ c(psi.19_20.g1, psi.19_20.g2)*RIFS

RIBV ~~ c(psi.19_21.g1, psi.19_21.g2)*RIIS

RIFS ~~ c(psi.20_21.g1, psi.20_21.g2)*RIIS

###############

# WITHIN PART #

###############

# Create within-components

T1WBV =~ c(NA, NA)*T1LBV + c(beta.1_10.g1, beta.1_10.g2)*T1LBV + start(0.1, 0.1)*T1LBV

T2WBV =~ c(NA, NA)*T2LBV + c(beta.2_13.g1, beta.2_13.g2)*T2LBV + start(0.1, 0.1)*T2LBV

T3WBV =~ c(NA, NA)*T3LBV + c(beta.3_16.g1, beta.3_16.g2)*T3LBV + start(0.1, 0.1)*T3LBV

T1WFS =~ c(NA, NA)*T1LFS + c(beta.4_11.g1, beta.4_11.g2)*T1LFS + start(0.1, 0.1)*T1LFS

T2WFS =~ c(NA, NA)*T2LFS + c(beta.5_14.g1, beta.5_14.g2)*T2LFS + start(0.1, 0.1)*T2LFS

T3WFS =~ c(NA, NA)*T3LFS + c(beta.6_17.g1, beta.6_17.g2)*T3LFS + start(0.1, 0.1)*T3LFS

T1WIS =~ c(NA, NA)*T1LIS + c(beta.7_12.g1, beta.7_12.g2)*T1LIS + start(0.1, 0.1)*T1LIS

T2WIS =~ c(NA, NA)*T2LIS + c(beta.8_15.g1, beta.8_15.g2)*T2LIS + start(0.1, 0.1)*T2LIS

T3WIS =~ c(NA, NA)*T3LIS + c(beta.9_18.g1, beta.9_18.g2)*T3LIS + start(0.1, 0.1)*T3LIS

T1WBV ~~ c(1, 1)*T1WBV

T2WBV ~~ c(1, 1)*T2WBV

T3WBV ~~ c(1, 1)*T3WBV

T1WFS ~~ c(1, 1)*T1WFS

T2WFS ~~ c(1, 1)*T2WFS

T3WFS ~~ c(1, 1)*T3WFS

T1WIS ~~ c(1, 1)*T1WIS

T2WIS ~~ c(1, 1)*T2WIS

T3WIS ~~ c(1, 1)*T3WIS

T1WBV ~~ c(NA, NA)*T1WFS + c(psi.10_11.g1, psi.10_11.g2)*T1WFS

T1WBV ~~ c(NA, NA)*T1WIS + c(psi.10_12.g1, psi.10_12.g2)*T1WIS

T1WFS ~~ c(NA, NA)*T1WIS + c(psi.11_12.g1, psi.11_12.g2)*T1WIS

T2WBV ~~ c(NA, NA)*T2WFS + c(psi.13_14.g1, psi.13_14.g2)*T2WFS

T2WBV ~~ c(NA, NA)*T2WIS + c(psi.13_15.g1, psi.13_15.g2)*T2WIS

T2WFS ~~ c(NA, NA)*T2WIS + c(psi.14_15.g1, psi.14_15.g2)*T2WIS

T3WBV ~~ c(NA, NA)*T3WFS + c(psi.16_17.g1, psi.16_17.g2)*T3WFS

T3WBV ~~ c(NA, NA)*T3WIS + c(psi.16_18.g1, psi.16_18.g2)*T3WIS

T3WFS ~~ c(NA, NA)*T3WIS + c(psi.17_18.g1, psi.17_18.g2)*T3WIS

###########

# CL + AR #

###########

T2WBV ~ c(beta.13_10.g1, beta.13_10.g2)*T1WBV + c(beta.13_11.g1, beta.13_11.g2)*T1WFS + c(beta.13_12.g1, beta.13_12.g2)*T1WIS

T2WFS ~ c(beta.14_10.g1, beta.14_10.g2)*T1WBV + c(beta.14_11.g1, beta.14_11.g2)*T1WFS + c(beta.14_12.g1, beta.14_12.g2)*T1WIS

T2WIS ~ c(beta.15_10.g1, beta.15_10.g2)*T1WBV + c(beta.15_11.g1, beta.15_11.g2)*T1WFS + c(beta.15_12.g1, beta.15_12.g2)*T1WIS

T3WBV ~ c(beta.16_13.g1, beta.16_13.g2)*T2WBV + c(beta.16_14.g1, beta.16_14.g2)*T2WFS + c(beta.16_15.g1, beta.16_15.g2)*T2WIS

T3WFS ~ c(beta.17_13.g1, beta.17_13.g2)*T2WBV + c(beta.17_14.g1, beta.17_14.g2)*T2WFS + c(beta.17_15.g1, beta.17_15.g2)*T2WIS

T3WIS ~ c(beta.18_13.g1, beta.18_13.g2)*T2WBV + c(beta.18_14.g1, beta.18_14.g2)*T2WFS + c(beta.18_15.g1, beta.18_15.g2)*T2WIS

##########################

# ADDITIONAL CONSTRAINTS #

##########################

RIBV + RIFS + RIIS ~~ c(0, 0)*T1WBV + c(0, 0)*T1WFS + c(0, 0)*T1WIS

RIBV ~ c(gamma.19_53.g1, gamma.19_53.g2)*ETHNICITY + c(gamma.19_54.g1, gamma.19_54.g2)*SEN + c(gamma.19_55.g1, gamma.19_55.g2)*FSMEVER

RIFS ~ c(gamma.20_53.g1, gamma.20_53.g2)*ETHNICITY + c(gamma.20_54.g1, gamma.20_54.g2)*SEN + c(gamma.20_55.g1, gamma.20_55.g2)*FSMEVER

RIIS ~ c(gamma.21_53.g1, gamma.21_53.g2)*ETHNICITY + c(gamma.21_54.g1, gamma.21_54.g2)*SEN + c(gamma.21_55.g1, gamma.21_55.g2)*FSMEVER

beta.13_10.g1 == beta.16_13.g1

beta.13_11.g1 == beta.16_14.g1

beta.13_12.g1 == beta.16_15.g1

beta.14_10.g1 == beta.17_13.g1

psi.13_14.g1 == psi.16_17.g1

psi.13_15.g1 == psi.16_18.g1

beta.13_11.g2 == beta.16_14.g2

beta.13_12.g2 == beta.16_15.g2

beta.15_10.g2 == beta.18_13.g2

beta.15_11.g2 == beta.18_14.g2

psi.13_14.g2 == psi.16_17.g2

psi.13_15.g2 == psi.16_18.g2

psi.14_15.g2 == psi.17_18.g2

beta.13_10.g2 == beta.16_13.g2”

MG.Model.RICLPM.fit <- *lavaan*(MG.Model.RICLPM,
 data = data,
 missing = 'FIML', estimator = 'MLR',group = "GENDER",
 meanstructure = T,
 int.ov.free = T, se = "robust")

# Appendix K) RI-CLPM Output

lavaan 0.6-18 ended normally after 228 iterations

Estimator ML

Optimization method NLMINB

Number of model parameters 348

Number of equality constraints 128

Row rank of the constraints matrix 116

Number of observations per group:

1 13395

0 13063

Number of missing patterns per group:

1 436

0 471

Model Test User Model:

Standard Scaled

Test Statistic 6029.308 5016.063

Degrees of freedom 740 740

P-value (Chi-square) 0.000 0.000

Scaling correction factor 1.202

Yuan-Bentler correction (Mplus variant)

Test statistic for each group:

1 3075.942 2559.020

0 2953.366 2457.043

Model Test Baseline Model:

Test statistic 187581.714 147736.588

Degrees of freedom 864 864

P-value 0.000 0.000

Scaling correction factor 1.270

User Model versus Baseline Model:

Comparative Fit Index (CFI) 0.972 0.971

Tucker-Lewis Index (TLI) 0.967 0.966

Robust Comparative Fit Index (CFI) 0.975

Robust Tucker-Lewis Index (TLI) 0.970

Loglikelihood and Information Criteria:

Loglikelihood user model (H0) -367733.361 -367733.361

Scaling correction factor 1.027

for the MLR correction

Loglikelihood unrestricted model (H1) -364718.707 -364718.707

Scaling correction factor 1.283

for the MLR correction

Akaike (AIC) 735930.722 735930.722

Bayesian (BIC) 737829.250 737829.250

Sample-size adjusted Bayesian (SABIC) 737091.959 737091.959

Root Mean Square Error of Approximation:

RMSEA 0.023 0.021

90 Percent confidence interval - lower 0.023 0.020

90 Percent confidence interval - upper 0.024 0.021

P-value H_0: RMSEA <= 0.050 1.000 1.000

P-value H_0: RMSEA >= 0.080 0.000 0.000

Robust RMSEA 0.029

90 Percent confidence interval - lower 0.029

90 Percent confidence interval - upper 0.030

P-value H_0: Robust RMSEA <= 0.050 1.000

P-value H_0: Robust RMSEA >= 0.080 0.000

Standardized Root Mean Square Residual:

SRMR 0.031 0.031

Parameter Estimates:

Standard errors Sandwich

Information bread Observed

Observed information based on Hessian

Group 1 [1]:

Latent Variables:

Estimate Std.Err z-value P(>|z|) ci.lower ci.upper Std.lv Std.all

T1LBV =~

T1BVP (l.1_) 1.010 0.006 156.935 0.000 0.997 1.023 0.511 0.740

T1BVP (l.2_) 1.306 0.008 155.431 0.000 1.289 1.322 0.660 0.769

T1BVP (l.3_) 0.684 0.008 86.101 0.000 0.669 0.700 0.346 0.565

T2LBV =~

T2BVP (l.1_) 1.010 0.006 156.935 0.000 0.997 1.023 0.538 0.745

T2BVP (l.2_) 1.306 0.008 155.431 0.000 1.289 1.322 0.695 0.782

T2BVP (l.3_) 0.684 0.008 86.101 0.000 0.669 0.700 0.364 0.597

T3LBV =~

T3BVP (l.1_) 1.010 0.006 156.935 0.000 0.997 1.023 0.523 0.760

T3BVP (l.2_) 1.306 0.008 155.431 0.000 1.289 1.322 0.676 0.813

T3BVP (l.3_) 0.684 0.008 86.101 0.000 0.669 0.700 0.354 0.608

T1LFS =~

T1FSP (l.10) 1.035 0.005 199.751 0.000 1.025 1.045 0.814 0.852

T1FSP (l.11) 0.965 0.005 186.280 0.000 0.955 0.975 0.759 0.700

T2LFS =~

T2FSP (l.10) 1.035 0.005 199.751 0.000 1.025 1.045 0.833 0.883

T2FSP (l.11) 0.965 0.005 186.280 0.000 0.955 0.975 0.777 0.703

T3LFS =~

T3FSP (l.10) 1.035 0.005 199.751 0.000 1.025 1.045 0.818 0.883

T3FSP (l.11) 0.965 0.005 186.280 0.000 0.955 0.975 0.762 0.724

T1LIS =~

T1ISP (l.16) 1.008 0.005 217.013 0.000 0.998 1.017 0.416 0.852

T1ISP (l.17) 1.135 0.004 267.592 0.000 1.127 1.143 0.469 0.786

T1ISP (l.18) 0.936 0.006 169.360 0.000 0.925 0.947 0.387 0.574

T1ISP (l.19) 0.921 0.005 195.228 0.000 0.912 0.931 0.381 0.659

T2LIS =~

T2ISP (l.16) 1.008 0.005 217.013 0.000 0.998 1.017 0.425 0.847

T2ISP (l.17) 1.135 0.004 267.592 0.000 1.127 1.143 0.479 0.782

T2ISP (l.18) 0.936 0.006 169.360 0.000 0.925 0.947 0.395 0.571

T2ISP (l.19) 0.921 0.005 195.228 0.000 0.912 0.931 0.388 0.668

T3LIS =~

T3ISP (l.16) 1.008 0.005 217.013 0.000 0.998 1.017 0.432 0.853

T3ISP (l.17) 1.135 0.004 267.592 0.000 1.127 1.143 0.486 0.802

T3ISP (l.18) 0.936 0.006 169.360 0.000 0.925 0.947 0.401 0.578

T3ISP (l.19) 0.921 0.005 195.228 0.000 0.912 0.931 0.395 0.691

RIBV =~

T1LBV (b.1_19) 0.310 0.018 17.563 0.000 0.275 0.344 0.632 0.632

T2LBV (b.1_19) 0.310 0.018 17.563 0.000 0.275 0.344 0.601 0.601

T3LBV (b.1_19) 0.310 0.018 17.563 0.000 0.275 0.344 0.617 0.617

RIFS =~

T1LFS (b.4_2) 0.435 0.025 17.528 0.000 0.386 0.483 0.567 0.567

T2LFS (b.4_2) 0.435 0.025 17.528 0.000 0.386 0.483 0.554 0.554

T3LFS (b.4_2) 0.435 0.025 17.528 0.000 0.386 0.483 0.565 0.565

RIIS =~

T1LIS (b.7_2) 0.268 0.009 28.632 0.000 0.250 0.286 0.662 0.662

T2LIS (b.7_2) 0.268 0.009 28.632 0.000 0.250 0.286 0.649 0.649

T3LIS (b.7_2) 0.268 0.009 28.632 0.000 0.250 0.286 0.638 0.638

T1WBV =~

T1LBV (b.1_10) 0.392 0.015 25.554 0.000 0.362 0.422 0.775 0.775

T2WBV =~

T2LBV (b.2_) 0.396 0.011 37.515 0.000 0.376 0.417 0.800 0.800

T3WBV =~

T3LBV (b.3_) 0.376 0.011 34.742 0.000 0.355 0.397 0.787 0.787

T1WFS =~

T1LFS (b.4_1) 0.648 0.018 36.304 0.000 0.613 0.683 0.824 0.824

T2WFS =~

T2LFS (b.5_) 0.635 0.013 47.833 0.000 0.609 0.661 0.833 0.833

T3WFS =~

T3LFS (b.6_) 0.616 0.012 52.071 0.000 0.593 0.640 0.825 0.825

T1WIS =~

T1LIS (b.7_1) 0.310 0.008 36.985 0.000 0.293 0.326 0.749 0.749

T2WIS =~

T2LIS (b.8_) 0.300 0.006 54.409 0.000 0.289 0.310 0.761 0.761

T3WIS =~

T3LIS (b.9_) 0.313 0.006 55.649 0.000 0.302 0.324 0.770 0.770

Regressions:

Estimate Std.Err z-value P(>|z|) ci.lower ci.upper Std.lv Std.all

T2WBV ~

T1WB (b.13_10) 0.369 0.058 6.322 0.000 0.255 0.484 0.344 0.344

T1WF (b.13_11) -0.051 0.038 -1.360 0.174 -0.125 0.023 -0.048 -0.048

T1WI (b.13_12) -0.004 0.039 -0.099 0.921 -0.080 0.072 -0.004 -0.004

T2WFS ~

T1WB (b.14_10) -0.079 0.038 -2.098 0.036 -0.152 -0.005 -0.075 -0.075

T1WF (b.14_11) 0.171 0.058 2.959 0.003 0.058 0.284 0.162 0.162

T1WI (b.14_12) -0.158 0.052 -3.027 0.002 -0.261 -0.056 -0.150 -0.150

T2WIS ~

T1WB (b.15_10) 0.046 0.045 1.026 0.305 -0.042 0.133 0.043 0.043

T1WF (b.15_11) -0.070 0.042 -1.668 0.095 -0.151 0.012 -0.065 -0.065

T1WI (b.15_12) 0.312 0.056 5.543 0.000 0.202 0.422 0.291 0.291

T3WBV ~

T2WB (b.16_13) 0.369 0.058 6.322 0.000 0.255 0.484 0.366 0.366

T2WF (b.16_14) -0.051 0.038 -1.360 0.174 -0.125 0.023 -0.050 -0.050

T2WI (b.16_15) -0.004 0.039 -0.099 0.921 -0.080 0.072 -0.004 -0.004

T3WFS ~

T2WB (b.17_13) -0.079 0.038 -2.098 0.036 -0.152 -0.005 -0.080 -0.080

T2WF (b.17_14) 0.234 0.051 4.595 0.000 0.134 0.334 0.234 0.234

T2WI (b.17_15) -0.075 0.044 -1.709 0.087 -0.161 0.011 -0.076 -0.076

T3WIS ~

T2WB (b.18_13) 0.138 0.041 3.375 0.001 0.058 0.217 0.140 0.140

T2WF (b.18_14) -0.099 0.039 -2.554 0.011 -0.175 -0.023 -0.100 -0.100

T2WI (b.18_15) 0.148 0.049 3.031 0.002 0.052 0.244 0.150 0.150

RIBV ~

ETHN (g.19_53) -0.394 0.036 -11.057 0.000 -0.464 -0.324 -0.381 -0.182

SEN (g.19_54) 0.404 0.057 7.049 0.000 0.291 0.516 0.391 0.127

FSME (g.19_55) 0.229 0.036 6.331 0.000 0.158 0.300 0.222 0.101

RIFS ~

ETHN (g.20_53) 0.146 0.033 4.399 0.000 0.081 0.211 0.142 0.068

SEN (g.20_54) -0.484 0.060 -8.006 0.000 -0.603 -0.366 -0.472 -0.153

FSME (g.20_55) -0.306 0.039 -7.759 0.000 -0.383 -0.229 -0.298 -0.135

RIIS ~

ETHN (g.21_53) -0.393 0.031 -12.610 0.000 -0.455 -0.332 -0.386 -0.184

SEN (g.21_54) 0.179 0.043 4.128 0.000 0.094 0.264 0.176 0.057

FSME (g.21_55) 0.087 0.031 2.843 0.004 0.027 0.147 0.085 0.039

Covariances:

Estimate Std.Err z-value P(>|z|) ci.lower ci.upper Std.lv Std.all

.T1BVP1 ~~

.T2BV (t.4_) 0.023 0.006 3.503 0.000 0.010 0.035 0.023 0.102

.T3BV (t.7_1) 0.026 0.006 4.536 0.000 0.015 0.037 0.026 0.124

.T1BVP2 ~~

.T2BV (t.5_) 0.055 0.009 6.480 0.000 0.039 0.072 0.055 0.182

.T3BV (t.8_2) 0.029 0.008 3.513 0.000 0.013 0.045 0.029 0.108

.T1BVP3 ~~

.T2BV (t.6_) 0.046 0.006 7.560 0.000 0.034 0.058 0.046 0.186

.T3BV (t.9_3) 0.027 0.006 4.797 0.000 0.016 0.038 0.027 0.116

.T2BVP1 ~~

.T3BV (t.7_4) 0.031 0.006 4.745 0.000 0.018 0.043 0.031 0.142

.T2BVP2 ~~

.T3BV (t.8_5) 0.052 0.009 6.027 0.000 0.035 0.069 0.052 0.195

.T2BVP3 ~~

.T3BV (t.9_6) 0.042 0.006 6.541 0.000 0.029 0.054 0.042 0.184

.T1FSP1 ~~

.T2FS (t.12) 0.044 0.009 5.008 0.000 0.027 0.061 0.044 0.198

.T3FS (t.14_10) 0.047 0.009 5.320 0.000 0.030 0.064 0.047 0.216

.T1FSP2 ~~

.T2FS (t.13) 0.125 0.012 9.985 0.000 0.100 0.149 0.125 0.204

.T3FS (t.15_11) 0.079 0.012 6.736 0.000 0.056 0.102 0.079 0.140

.T2FSP1 ~~

.T3FS (t.14_12) 0.050 0.009 5.722 0.000 0.033 0.067 0.050 0.261

.T2FSP2 ~~

.T3FS (t.15_13) 0.126 0.012 10.578 0.000 0.102 0.149 0.126 0.220

.T1ISP1 ~~

.T2IS (t.20) 0.008 0.002 4.583 0.000 0.004 0.011 0.008 0.111

.T3IS (t.24_1) 0.005 0.002 3.428 0.001 0.002 0.009 0.005 0.080

.T1ISP2 ~~

.T2IS (t.21) 0.046 0.003 16.850 0.000 0.041 0.051 0.046 0.327

.T3IS (t.25_1) 0.037 0.003 14.282 0.000 0.032 0.042 0.037 0.275

.T1ISP3 ~~

.T2IS (t.22) 0.121 0.005 25.365 0.000 0.112 0.130 0.121 0.386

.T3IS (t.26_1) 0.102 0.005 21.610 0.000 0.093 0.111 0.102 0.327

.T1ISP4 ~~

.T2IS (t.23) 0.075 0.003 25.378 0.000 0.069 0.081 0.075 0.400

.T3IS (t.27_1) 0.057 0.003 19.391 0.000 0.051 0.062 0.057 0.315

.T2ISP1 ~~

.T3IS (t.24_2) 0.011 0.002 6.102 0.000 0.007 0.014 0.011 0.153

.T2ISP2 ~~

.T3IS (t.25_2) 0.050 0.003 17.799 0.000 0.044 0.055 0.050 0.359

.T2ISP3 ~~

.T3IS (t.26_2) 0.134 0.005 26.906 0.000 0.125 0.144 0.134 0.418

.T2ISP4 ~~

.T3IS (t.27_2) 0.064 0.003 21.283 0.000 0.058 0.070 0.064 0.359

.RIBV ~~

.RIFS (p.19_20) -0.555 0.056 -9.905 0.000 -0.664 -0.445 -0.555 -0.555

.RIIS (p.19_21) 0.598 0.042 14.296 0.000 0.516 0.680 0.598 0.598

.RIFS ~~

.RIIS (p.20) -0.727 0.036 -20.019 0.000 -0.799 -0.656 -0.727 -0.727

T1WBV ~~

T1WF (p.10_11) -0.414 0.035 -11.664 0.000 -0.483 -0.344 -0.414 -0.414

T1WI (p.10_12) 0.524 0.032 16.149 0.000 0.460 0.588 0.524 0.524

T1WFS ~~

T1WI (p.11) -0.579 0.026 -22.613 0.000 -0.629 -0.529 -0.579 -0.579

.T2WBV ~~

.T2WF (p.13_14) -0.346 0.021 -16.692 0.000 -0.387 -0.306 -0.346 -0.346

.T2WI (p.13_15) 0.405 0.020 20.420 0.000 0.366 0.444 0.405 0.405

.T2WFS ~~

.T2WI (p.14) -0.487 0.023 -20.932 0.000 -0.532 -0.441 -0.487 -0.487

.T3WBV ~~

.T3WF (p.16_17) -0.346 0.021 -16.692 0.000 -0.387 -0.306 -0.346 -0.346

.T3WI (p.16_18) 0.405 0.020 20.420 0.000 0.366 0.444 0.405 0.405

.T3WFS ~~

.T3WI (p.17) -0.428 0.022 -19.346 0.000 -0.472 -0.385 -0.428 -0.428

.RIBV ~~

T1WB 0.000 0.000 0.000 0.000 0.000

T1WF 0.000 0.000 0.000 0.000 0.000

T1WI 0.000 0.000 0.000 0.000 0.000

.RIFS ~~

T1WB 0.000 0.000 0.000 0.000 0.000

T1WF 0.000 0.000 0.000 0.000 0.000

T1WI 0.000 0.000 0.000 0.000 0.000

.RIIS ~~

T1WB 0.000 0.000 0.000 0.000 0.000

T1WF 0.000 0.000 0.000 0.000 0.000

T1WI 0.000 0.000 0.000 0.000 0.000

Intercepts:

Estimate Std.Err z-value P(>|z|) ci.lower ci.upper Std.lv Std.all

.T1BVP1 (n.1.) -0.087 0.008 -10.448 0.000 -0.104 -0.071 -0.087 -0.126

.T1BVP2 (n.2.) -0.229 0.011 -20.144 0.000 -0.251 -0.206 -0.229 -0.266

.T1BVP3 (n.3.) 0.316 0.010 30.494 0.000 0.296 0.336 0.316 0.516

.T2BVP1 (n.1.) -0.087 0.008 -10.448 0.000 -0.104 -0.071 -0.087 -0.121

.T2BVP2 (n.2.) -0.229 0.011 -20.144 0.000 -0.251 -0.206 -0.229 -0.257

.T2BVP3 (n.3.) 0.316 0.010 30.494 0.000 0.296 0.336 0.316 0.518

.T3BVP1 (n.1.) -0.087 0.008 -10.448 0.000 -0.104 -0.071 -0.087 -0.127

.T3BVP2 (n.2.) -0.229 0.011 -20.144 0.000 -0.251 -0.206 -0.229 -0.275

.T3BVP3 (n.3.) 0.316 0.010 30.494 0.000 0.296 0.336 0.316 0.542

.T1FSP1 (n.10) -0.241 0.021 -11.590 0.000 -0.282 -0.200 -0.241 -0.252

.T1FSP2 (n.11) 0.241 0.021 11.590 0.000 0.200 0.282 0.241 0.222

.T2FSP1 (n.10) -0.241 0.021 -11.590 0.000 -0.282 -0.200 -0.241 -0.256

.T2FSP2 (n.11) 0.241 0.021 11.590 0.000 0.200 0.282 0.241 0.218

.T3FSP1 (n.10) -0.241 0.021 -11.590 0.000 -0.282 -0.200 -0.241 -0.261

.T3FSP2 (n.11) 0.241 0.021 11.590 0.000 0.200 0.282 0.241 0.229

.T1ISP1 (n.16) -0.080 0.004 -19.790 0.000 -0.088 -0.072 -0.080 -0.163

.T1ISP2 (n.17) 0.020 0.004 4.987 0.000 0.012 0.028 0.020 0.034

.T1ISP3 (n.18) 0.099 0.005 18.181 0.000 0.088 0.109 0.099 0.146

.T1ISP4 (n.19) -0.039 0.004 -8.993 0.000 -0.048 -0.031 -0.039 -0.068

.T2ISP1 (n.16) -0.080 0.004 -19.790 0.000 -0.088 -0.072 -0.080 -0.159

.T2ISP2 (n.17) 0.020 0.004 4.987 0.000 0.012 0.028 0.020 0.033

.T2ISP3 (n.18) 0.099 0.005 18.181 0.000 0.088 0.109 0.099 0.143

.T2ISP4 (n.19) -0.039 0.004 -8.993 0.000 -0.048 -0.031 -0.039 -0.067

.T3ISP1 (n.16) -0.080 0.004 -19.790 0.000 -0.088 -0.072 -0.080 -0.158

.T3ISP2 (n.17) 0.020 0.004 4.987 0.000 0.012 0.028 0.020 0.034

.T3ISP3 (n.18) 0.099 0.005 18.181 0.000 0.088 0.109 0.099 0.142

.T3ISP4 (n.19) -0.039 0.004 -8.993 0.000 -0.048 -0.031 -0.039 -0.069

.T1LBV (a.1.) 1.426 0.007 190.621 0.000 1.412 1.441 2.820 2.820

.T2LBV (a.2.) 1.436 0.008 181.118 0.000 1.420 1.451 2.697 2.697

.T3LBV (a.3.) 1.372 0.008 171.459 0.000 1.356 1.388 2.649 2.649

.T1LFS (a.4.) 3.886 0.011 348.140 0.000 3.864 3.907 4.938 4.938

.T2LFS (a.5.) 3.810 0.012 330.282 0.000 3.787 3.833 4.731 4.731

.T3LFS (a.6.) 3.868 0.012 330.888 0.000 3.845 3.891 4.897 4.897

.T1LIS (a.7.) 0.826 0.006 144.586 0.000 0.814 0.837 1.999 1.999

.T2LIS (a.8.) 0.846 0.006 141.901 0.000 0.835 0.858 2.008 2.008

.T3LIS (a.9.) 0.801 0.006 129.826 0.000 0.789 0.813 1.870 1.870

Variances:

Estimate Std.Err z-value P(>|z|) ci.lower ci.upper Std.lv Std.all

.T1BVP1 (t.1_) 0.215 0.007 29.457 0.000 0.201 0.229 0.215 0.452

.T1BVP2 (t.2_) 0.302 0.010 29.197 0.000 0.281 0.322 0.302 0.409

.T1BVP3 (t.3_) 0.255 0.008 32.897 0.000 0.240 0.271 0.255 0.681

.T2BVP1 (t.4_) 0.232 0.008 27.792 0.000 0.215 0.248 0.232 0.445

.T2BVP2 (t.5_) 0.307 0.012 26.648 0.000 0.284 0.329 0.307 0.388

.T2BVP3 (t.6_) 0.240 0.008 31.378 0.000 0.225 0.255 0.240 0.644

.T3BVP1 (t.7_) 0.200 0.009 22.849 0.000 0.183 0.217 0.200 0.422

.T3BVP2 (t.8_) 0.235 0.011 22.096 0.000 0.214 0.256 0.235 0.339

.T3BVP3 (t.9_) 0.214 0.008 26.202 0.000 0.198 0.230 0.214 0.630

.T1FSP1 (t.10) 0.250 0.011 23.278 0.000 0.229 0.271 0.250 0.274

.T1FSP2 (t.11) 0.601 0.013 47.343 0.000 0.576 0.626 0.601 0.510

.T2FSP1 (t.12) 0.196 0.011 18.198 0.000 0.175 0.217 0.196 0.220

.T2FSP2 (t.13) 0.619 0.014 45.849 0.000 0.593 0.645 0.619 0.506

.T3FSP1 (t.14) 0.188 0.010 18.169 0.000 0.168 0.209 0.188 0.220

.T3FSP2 (t.15) 0.527 0.013 41.519 0.000 0.502 0.552 0.527 0.476

.T1ISP1 (t.16) 0.065 0.002 37.869 0.000 0.062 0.069 0.065 0.274

.T1ISP2 (t.17) 0.136 0.003 48.063 0.000 0.130 0.141 0.136 0.382

.T1ISP3 (t.18) 0.305 0.004 68.152 0.000 0.296 0.313 0.305 0.671

.T1ISP4 (t.19) 0.189 0.003 61.812 0.000 0.183 0.195 0.189 0.566

.T2ISP1 (t.20) 0.071 0.002 34.300 0.000 0.067 0.075 0.071 0.283

.T2ISP2 (t.21) 0.145 0.003 45.006 0.000 0.139 0.151 0.145 0.388

.T2ISP3 (t.22) 0.323 0.005 63.684 0.000 0.313 0.333 0.323 0.674

.T2ISP4 (t.23) 0.187 0.003 55.034 0.000 0.180 0.194 0.187 0.553

.T3ISP1 (t.24) 0.070 0.002 32.078 0.000 0.065 0.074 0.070 0.272

.T3ISP2 (t.25) 0.132 0.003 42.513 0.000 0.126 0.138 0.132 0.358

.T3ISP3 (t.26) 0.321 0.005 59.334 0.000 0.310 0.331 0.321 0.666

.T3ISP4 (t.27) 0.170 0.003 52.921 0.000 0.164 0.176 0.170 0.522

.T1LBV 0.000 0.000 0.000 0.000 0.000

.T2LBV 0.000 0.000 0.000 0.000 0.000

.T3LBV 0.000 0.000 0.000 0.000 0.000

.T1LFS 0.000 0.000 0.000 0.000 0.000

.T2LFS 0.000 0.000 0.000 0.000 0.000

.T3LFS 0.000 0.000 0.000 0.000 0.000

.T1LIS 0.000 0.000 0.000 0.000 0.000

.T2LIS 0.000 0.000 0.000 0.000 0.000

.T3LIS 0.000 0.000 0.000 0.000 0.000

.RIBV 1.000 1.000 1.000 0.938 0.938

.RIFS 1.000 1.000 1.000 0.949 0.949

.RIIS 1.000 1.000 1.000 0.961 0.961

T1WBV 1.000 1.000 1.000 1.000 1.000

.T2WBV 1.000 1.000 1.000 0.867 0.867

.T3WBV 1.000 1.000 1.000 0.851 0.851

T1WFS 1.000 1.000 1.000 1.000 1.000

.T2WFS 1.000 1.000 1.000 0.896 0.896

.T3WFS 1.000 1.000 1.000 0.894 0.894

T1WIS 1.000 1.000 1.000 1.000 1.000

.T2WIS 1.000 1.000 1.000 0.872 0.872

.T3WIS 1.000 1.000 1.000 0.902 0.902

Group 2 [0]:

Latent Variables:

Estimate Std.Err z-value P(>|z|) ci.lower ci.upper Std.lv Std.all

T1LBV =~

T1BVP (l.1_) 1.010 0.006 156.935 0.000 0.997 1.023 0.522 0.751

T1BVP (l.2_) 1.306 0.008 155.431 0.000 1.289 1.322 0.674 0.817

T1BVP (l.3_) 0.684 0.008 86.101 0.000 0.669 0.700 0.353 0.580

T2LBV =~

T2BVP (l.1_) 1.010 0.006 156.935 0.000 0.997 1.023 0.578 0.767

T2BVP (l.2_) 1.306 0.008 155.431 0.000 1.289 1.322 0.748 0.864

T2BVP (l.3_) 0.684 0.008 86.101 0.000 0.669 0.700 0.392 0.608

T3LBV =~

T3BVP (l.1_) 1.010 0.006 156.935 0.000 0.997 1.023 0.608 0.797

T3BVP (l.2_) 1.306 0.008 155.431 0.000 1.289 1.322 0.785 0.890

T3BVP (l.3_) 0.684 0.008 86.101 0.000 0.669 0.700 0.412 0.608

T1LFS =~

T1FSP (l.10) 1.035 0.005 199.751 0.000 1.025 1.045 0.818 0.882

T1FSP (l.11) 0.965 0.005 186.280 0.000 0.955 0.975 0.762 0.718

T2LFS =~

T2FSP (l.10) 1.035 0.005 199.751 0.000 1.025 1.045 0.863 0.907

T2FSP (l.11) 0.965 0.005 186.280 0.000 0.955 0.975 0.805 0.737

T3LFS =~

T3FSP (l.10) 1.035 0.005 199.751 0.000 1.025 1.045 0.891 0.910

T3FSP (l.11) 0.965 0.005 186.280 0.000 0.955 0.975 0.831 0.748

T1LIS =~

T1ISP (l.16) 1.008 0.005 217.013 0.000 0.998 1.017 0.366 0.793

T1ISP (l.17) 1.135 0.004 267.592 0.000 1.127 1.143 0.412 0.794

T1ISP (l.18) 0.936 0.006 169.360 0.000 0.925 0.947 0.340 0.543

T1ISP (l.19) 0.921 0.005 195.228 0.000 0.912 0.931 0.334 0.658

T2LIS =~

T2ISP (l.16) 1.008 0.005 217.013 0.000 0.998 1.017 0.395 0.812

T2ISP (l.17) 1.135 0.004 267.592 0.000 1.127 1.143 0.445 0.844

T2ISP (l.18) 0.936 0.006 169.360 0.000 0.925 0.947 0.367 0.565

T2ISP (l.19) 0.921 0.005 195.228 0.000 0.912 0.931 0.361 0.705

T3LIS =~

T3ISP (l.16) 1.008 0.005 217.013 0.000 0.998 1.017 0.425 0.828

T3ISP (l.17) 1.135 0.004 267.592 0.000 1.127 1.143 0.479 0.879

T3ISP (l.18) 0.936 0.006 169.360 0.000 0.925 0.947 0.395 0.600

T3ISP (l.19) 0.921 0.005 195.228 0.000 0.912 0.931 0.389 0.748

RIBV =~

T1LBV (b.1_19) 0.303 0.017 18.080 0.000 0.270 0.336 0.600 0.600

T2LBV (b.1_19) 0.303 0.017 18.080 0.000 0.270 0.336 0.541 0.541

T3LBV (b.1_19) 0.303 0.017 18.080 0.000 0.270 0.336 0.515 0.515

RIFS =~

T1LFS (b.4_2) 0.475 0.021 22.373 0.000 0.433 0.517 0.623 0.623

T2LFS (b.4_2) 0.475 0.021 22.373 0.000 0.433 0.517 0.590 0.590

T3LFS (b.4_2) 0.475 0.021 22.373 0.000 0.433 0.517 0.572 0.572

RIIS =~

T1LIS (b.7_2) 0.234 0.009 25.323 0.000 0.215 0.252 0.662 0.662

T2LIS (b.7_2) 0.234 0.009 25.323 0.000 0.215 0.252 0.612 0.612

T3LIS (b.7_2) 0.234 0.009 25.323 0.000 0.215 0.252 0.569 0.569

T1WBV =~

T1LBV (b.1_10) 0.413 0.014 28.764 0.000 0.385 0.441 0.800 0.800

T2WBV =~

T2LBV (b.2_) 0.463 0.011 42.050 0.000 0.441 0.484 0.841 0.841

T3WBV =~

T3LBV (b.3_) 0.493 0.012 42.128 0.000 0.470 0.516 0.857 0.857

T1WFS =~

T1LFS (b.4_1) 0.618 0.018 34.340 0.000 0.582 0.653 0.782 0.782

T2WFS =~

T2LFS (b.5_) 0.652 0.014 45.861 0.000 0.624 0.680 0.807 0.807

T3WFS =~

T3LFS (b.6_) 0.675 0.013 52.739 0.000 0.650 0.700 0.820 0.820

T1WIS =~

T1LIS (b.7_1) 0.272 0.008 32.493 0.000 0.256 0.288 0.750 0.750

T2WIS =~

T2LIS (b.8_) 0.296 0.007 45.377 0.000 0.283 0.309 0.791 0.791

T3WIS =~

T3LIS (b.9_) 0.337 0.007 48.459 0.000 0.323 0.351 0.822 0.822

Regressions:

Estimate Std.Err z-value P(>|z|) ci.lower ci.upper Std.lv Std.all

T2WBV ~

T1WB (b.13_10) 0.242 0.043 5.597 0.000 0.157 0.327 0.233 0.233

T1WF (b.13_11) 0.000 0.032 0.005 0.996 -0.062 0.063 0.000 0.000

T1WI (b.13_12) 0.084 0.037 2.299 0.021 0.012 0.156 0.081 0.081

T2WFS ~

T1WB (b.14_10) 0.065 0.043 1.533 0.125 -0.018 0.149 0.063 0.063

T1WF (b.14_11) 0.150 0.055 2.717 0.007 0.042 0.258 0.145 0.145

T1WI (b.14_12) -0.175 0.052 -3.370 0.001 -0.277 -0.073 -0.169 -0.169

T2WIS ~

T1WB (b.15_10) 0.091 0.034 2.681 0.007 0.024 0.157 0.087 0.087

T1WF (b.15_11) -0.035 0.032 -1.109 0.267 -0.098 0.027 -0.034 -0.034

T1WI (b.15_12) 0.237 0.050 4.703 0.000 0.138 0.336 0.227 0.227

T3WBV ~

T2WB (b.16_13) 0.242 0.043 5.597 0.000 0.157 0.327 0.241 0.241

T2WF (b.16_14) 0.000 0.032 0.005 0.996 -0.062 0.063 0.000 0.000

T2WI (b.16_15) 0.084 0.037 2.299 0.021 0.012 0.156 0.084 0.084

T3WFS ~

T2WB (b.17_13) -0.028 0.037 -0.752 0.452 -0.102 0.045 -0.028 -0.028

T2WF (b.17_14) 0.207 0.047 4.355 0.000 0.114 0.300 0.204 0.204

T2WI (b.17_15) -0.120 0.040 -2.978 0.003 -0.199 -0.041 -0.120 -0.120

T3WIS ~

T2WB (b.18_13) 0.091 0.034 2.681 0.007 0.024 0.157 0.092 0.092

T2WF (b.18_14) -0.035 0.032 -1.109 0.267 -0.098 0.027 -0.036 -0.036

T2WI (b.18_15) 0.155 0.043 3.634 0.000 0.072 0.239 0.158 0.158

RIBV ~

ETHN (g.19_53) -0.211 0.033 -6.361 0.000 -0.276 -0.146 -0.207 -0.098

SEN (g.19_54) 0.426 0.049 8.705 0.000 0.330 0.522 0.417 0.169

FSME (g.19_55) 0.095 0.037 2.588 0.010 0.023 0.166 0.093 0.042

RIFS ~

ETHN (g.20_53) 0.269 0.033 8.092 0.000 0.203 0.334 0.259 0.123

SEN (g.20_54) -0.438 0.043 -10.169 0.000 -0.523 -0.354 -0.423 -0.172

FSME (g.20_55) -0.316 0.037 -8.583 0.000 -0.388 -0.244 -0.305 -0.137

RIIS ~

ETHN (g.21_53) -0.342 0.031 -10.884 0.000 -0.403 -0.280 -0.332 -0.158

SEN (g.21_54) 0.385 0.040 9.611 0.000 0.306 0.463 0.374 0.152

FSME (g.21_55) 0.120 0.033 3.635 0.000 0.056 0.185 0.117 0.053

Covariances:

Estimate Std.Err z-value P(>|z|) ci.lower ci.upper Std.lv Std.all

.T1BVP1 ~~

.T2BV (t.4_) 0.024 0.006 4.007 0.000 0.012 0.036 0.024 0.108

.T3BV (t.7_1) 0.008 0.005 1.552 0.121 -0.002 0.018 0.008 0.038

.T1BVP2 ~~

.T2BV (t.5_) 0.024 0.007 3.376 0.001 0.010 0.038 0.024 0.115

.T3BV (t.8_2) 0.020 0.007 2.871 0.004 0.006 0.034 0.020 0.106

.T1BVP3 ~~

.T2BV (t.6_) 0.045 0.007 6.799 0.000 0.032 0.058 0.045 0.176

.T3BV (t.9_3) 0.023 0.006 3.892 0.000 0.011 0.034 0.023 0.086

.T2BVP1 ~~

.T3BV (t.7_4) 0.031 0.006 5.091 0.000 0.019 0.043 0.031 0.140

.T2BVP2 ~~

.T3BV (t.8_5) 0.022 0.008 2.853 0.004 0.007 0.037 0.022 0.124

.T2BVP3 ~~

.T3BV (t.9_6) 0.049 0.008 6.409 0.000 0.034 0.064 0.049 0.177

.T1FSP1 ~~

.T2FS (t.12) 0.014 0.008 1.709 0.088 -0.002 0.031 0.014 0.082

.T3FS (t.14_10) 0.026 0.009 2.942 0.003 0.009 0.043 0.026 0.145

.T1FSP2 ~~

.T2FS (t.13) 0.128 0.011 11.434 0.000 0.106 0.150 0.128 0.234

.T3FS (t.15_11) 0.081 0.011 7.100 0.000 0.059 0.103 0.081 0.148

.T2FSP1 ~~

.T3FS (t.14_12) 0.009 0.009 0.957 0.339 -0.009 0.028 0.009 0.056

.T2FSP2 ~~

.T3FS (t.15_13) 0.135 0.012 11.142 0.000 0.111 0.159 0.135 0.248

.T1ISP1 ~~

.T2IS (t.20) 0.013 0.002 7.484 0.000 0.009 0.016 0.013 0.158

.T3IS (t.24_1) 0.010 0.002 5.748 0.000 0.007 0.014 0.010 0.128

.T1ISP2 ~~

.T2IS (t.21) 0.024 0.002 10.857 0.000 0.019 0.028 0.024 0.265

.T3IS (t.25_1) 0.018 0.002 8.577 0.000 0.014 0.022 0.018 0.220

.T1ISP3 ~~

.T2IS (t.22) 0.112 0.004 24.960 0.000 0.103 0.121 0.112 0.397

.T3IS (t.26_1) 0.088 0.005 18.732 0.000 0.079 0.097 0.088 0.318

.T1ISP4 ~~

.T2IS (t.23) 0.045 0.002 18.355 0.000 0.040 0.050 0.045 0.322

.T3IS (t.27_1) 0.033 0.002 14.030 0.000 0.029 0.038 0.033 0.252

.T2ISP1 ~~

.T3IS (t.24_2) 0.015 0.002 7.923 0.000 0.011 0.019 0.015 0.181

.T2ISP2 ~~

.T3IS (t.25_2) 0.020 0.002 9.849 0.000 0.016 0.024 0.020 0.273

.T2ISP3 ~~

.T3IS (t.26_2) 0.116 0.005 24.193 0.000 0.106 0.125 0.116 0.410

.T2ISP4 ~~

.T3IS (t.27_2) 0.035 0.002 14.979 0.000 0.030 0.039 0.035 0.276

.RIBV ~~

.RIFS (p.19_20) -0.673 0.054 -12.450 0.000 -0.779 -0.567 -0.673 -0.673

.RIIS (p.19_21) 0.704 0.044 16.061 0.000 0.618 0.790 0.704 0.704

.RIFS ~~

.RIIS (p.20) -0.742 0.036 -20.537 0.000 -0.813 -0.671 -0.742 -0.742

T1WBV ~~

T1WF (p.10_11) -0.345 0.037 -9.230 0.000 -0.419 -0.272 -0.345 -0.345

T1WI (p.10_12) 0.452 0.035 12.829 0.000 0.383 0.521 0.452 0.452

T1WFS ~~

T1WI (p.11) -0.481 0.031 -15.409 0.000 -0.542 -0.420 -0.481 -0.481

.T2WBV ~~

.T2WF (p.13_14) -0.398 0.020 -20.074 0.000 -0.437 -0.359 -0.398 -0.398

.T2WI (p.13_15) 0.466 0.020 22.986 0.000 0.426 0.506 0.466 0.466

.T2WFS ~~

.T2WI (p.14) -0.425 0.019 -22.189 0.000 -0.463 -0.388 -0.425 -0.425

.T3WBV ~~

.T3WF (p.16_17) -0.398 0.020 -20.074 0.000 -0.437 -0.359 -0.398 -0.398

.T3WI (p.16_18) 0.466 0.020 22.986 0.000 0.426 0.506 0.466 0.466

.T3WFS ~~

.T3WI (p.17) -0.425 0.019 -22.189 0.000 -0.463 -0.388 -0.425 -0.425

.RIBV ~~

T1WB 0.000 0.000 0.000 0.000 0.000

T1WF 0.000 0.000 0.000 0.000 0.000

T1WI 0.000 0.000 0.000 0.000 0.000

.RIFS ~~

T1WB 0.000 0.000 0.000 0.000 0.000

T1WF 0.000 0.000 0.000 0.000 0.000

T1WI 0.000 0.000 0.000 0.000 0.000

.RIIS ~~

T1WB 0.000 0.000 0.000 0.000 0.000

T1WF 0.000 0.000 0.000 0.000 0.000

T1WI 0.000 0.000 0.000 0.000 0.000

Intercepts:

Estimate Std.Err z-value P(>|z|) ci.lower ci.upper Std.lv Std.all

.T1BVP1 (n.1.) -0.006 0.008 -0.738 0.461 -0.021 0.010 -0.006 -0.008

.T1BVP2 (n.2.) -0.298 0.010 -28.628 0.000 -0.318 -0.278 -0.298 -0.361

.T1BVP3 (n.3.) 0.304 0.010 31.991 0.000 0.285 0.323 0.304 0.498

.T2BVP1 (n.1.) -0.006 0.008 -0.738 0.461 -0.021 0.010 -0.006 -0.008

.T2BVP2 (n.2.) -0.298 0.010 -28.628 0.000 -0.318 -0.278 -0.298 -0.344

.T2BVP3 (n.3.) 0.304 0.010 31.991 0.000 0.285 0.323 0.304 0.471

.T3BVP1 (n.1.) -0.006 0.008 -0.738 0.461 -0.021 0.010 -0.006 -0.008

.T3BVP2 (n.2.) -0.298 0.010 -28.628 0.000 -0.318 -0.278 -0.298 -0.338

.T3BVP3 (n.3.) 0.304 0.010 31.991 0.000 0.285 0.323 0.304 0.449

.T1FSP1 (n.10) -0.119 0.021 -5.762 0.000 -0.159 -0.078 -0.119 -0.128

.T1FSP2 (n.11) 0.119 0.021 5.762 0.000 0.078 0.159 0.119 0.112

.T2FSP1 (n.10) -0.119 0.021 -5.762 0.000 -0.159 -0.078 -0.119 -0.125

.T2FSP2 (n.11) 0.119 0.021 5.762 0.000 0.078 0.159 0.119 0.109

.T3FSP1 (n.10) -0.119 0.021 -5.762 0.000 -0.159 -0.078 -0.119 -0.121

.T3FSP2 (n.11) 0.119 0.021 5.762 0.000 0.078 0.159 0.119 0.107

.T1ISP1 (n.16) 0.008 0.003 2.676 0.007 0.002 0.013 0.008 0.016

.T1ISP2 (n.17) -0.133 0.003 -51.400 0.000 -0.138 -0.128 -0.133 -0.257

.T1ISP3 (n.18) 0.166 0.004 41.211 0.000 0.158 0.174 0.166 0.265

.T1ISP4 (n.19) -0.040 0.003 -14.013 0.000 -0.046 -0.035 -0.040 -0.079

.T2ISP1 (n.16) 0.008 0.003 2.676 0.007 0.002 0.013 0.008 0.015

.T2ISP2 (n.17) -0.133 0.003 -51.400 0.000 -0.138 -0.128 -0.133 -0.253

.T2ISP3 (n.18) 0.166 0.004 41.211 0.000 0.158 0.174 0.166 0.255

.T2ISP4 (n.19) -0.040 0.003 -14.013 0.000 -0.046 -0.035 -0.040 -0.078

.T3ISP1 (n.16) 0.008 0.003 2.676 0.007 0.002 0.013 0.008 0.015

.T3ISP2 (n.17) -0.133 0.003 -51.400 0.000 -0.138 -0.128 -0.133 -0.244

.T3ISP3 (n.18) 0.166 0.004 41.211 0.000 0.158 0.174 0.166 0.252

.T3ISP4 (n.19) -0.040 0.003 -14.013 0.000 -0.046 -0.035 -0.040 -0.077

.T1LBV (a.1.) 1.337 0.008 175.768 0.000 1.322 1.352 2.589 2.589

.T2LBV (a.2.) 1.358 0.008 164.850 0.000 1.342 1.374 2.371 2.371

.T3LBV (a.3.) 1.334 0.009 153.932 0.000 1.317 1.351 2.219 2.219

.T1LFS (a.4.) 3.895 0.011 345.627 0.000 3.873 3.918 4.931 4.931

.T2LFS (a.5.) 3.840 0.012 324.202 0.000 3.817 3.863 4.604 4.604

.T3LFS (a.6.) 3.806 0.013 300.576 0.000 3.782 3.831 4.422 4.422

.T1LIS (a.7.) 0.530 0.005 101.132 0.000 0.520 0.540 1.460 1.460

.T2LIS (a.8.) 0.499 0.006 90.254 0.000 0.488 0.510 1.273 1.273

.T3LIS (a.9.) 0.481 0.006 80.070 0.000 0.469 0.493 1.140 1.140

Variances:

Estimate Std.Err z-value P(>|z|) ci.lower ci.upper Std.lv Std.all

.T1BVP1 (t.1_) 0.211 0.008 27.324 0.000 0.195 0.226 0.211 0.436

.T1BVP2 (t.2_) 0.227 0.010 22.088 0.000 0.207 0.247 0.227 0.333

.T1BVP3 (t.3_) 0.247 0.009 26.534 0.000 0.229 0.265 0.247 0.664

.T2BVP1 (t.4_) 0.234 0.009 26.066 0.000 0.217 0.252 0.234 0.412

.T2BVP2 (t.5_) 0.190 0.010 18.422 0.000 0.170 0.210 0.190 0.254

.T2BVP3 (t.6_) 0.262 0.010 26.770 0.000 0.243 0.281 0.262 0.630

.T3BVP1 (t.7_) 0.211 0.009 22.318 0.000 0.193 0.230 0.211 0.364

.T3BVP2 (t.8_) 0.161 0.010 15.639 0.000 0.141 0.182 0.161 0.207

.T3BVP3 (t.9_) 0.289 0.012 24.975 0.000 0.266 0.311 0.289 0.630

.T1FSP1 (t.10) 0.190 0.010 18.929 0.000 0.170 0.210 0.190 0.221

.T1FSP2 (t.11) 0.548 0.013 43.597 0.000 0.523 0.573 0.548 0.485

.T2FSP1 (t.12) 0.160 0.011 14.768 0.000 0.139 0.181 0.160 0.177

.T2FSP2 (t.13) 0.543 0.014 39.263 0.000 0.516 0.571 0.543 0.456

.T3FSP1 (t.14) 0.164 0.012 14.220 0.000 0.142 0.187 0.164 0.171

.T3FSP2 (t.15) 0.545 0.015 36.874 0.000 0.516 0.574 0.545 0.441

.T1ISP1 (t.16) 0.079 0.002 40.752 0.000 0.075 0.083 0.079 0.371

.T1ISP2 (t.17) 0.099 0.003 38.005 0.000 0.094 0.104 0.099 0.369

.T1ISP3 (t.18) 0.276 0.005 60.541 0.000 0.267 0.285 0.276 0.705

.T1ISP4 (t.19) 0.147 0.003 53.353 0.000 0.141 0.152 0.147 0.567

.T2ISP1 (t.20) 0.081 0.002 35.667 0.000 0.076 0.085 0.081 0.340

.T2ISP2 (t.21) 0.080 0.003 31.947 0.000 0.075 0.085 0.080 0.287

.T2ISP3 (t.22) 0.288 0.005 54.928 0.000 0.277 0.298 0.288 0.681

.T2ISP4 (t.23) 0.132 0.003 48.212 0.000 0.127 0.138 0.132 0.504

.T3ISP1 (t.24) 0.083 0.003 32.388 0.000 0.078 0.088 0.083 0.315

.T3ISP2 (t.25) 0.067 0.003 26.085 0.000 0.062 0.072 0.067 0.227

.T3ISP3 (t.26) 0.277 0.005 50.496 0.000 0.266 0.288 0.277 0.640

.T3ISP4 (t.27) 0.119 0.003 42.497 0.000 0.114 0.125 0.119 0.441

.T1LBV 0.000 0.000 0.000 0.000 0.000

.T2LBV 0.000 0.000 0.000 0.000 0.000

.T3LBV 0.000 0.000 0.000 0.000 0.000

.T1LFS 0.000 0.000 0.000 0.000 0.000

.T2LFS 0.000 0.000 0.000 0.000 0.000

.T3LFS 0.000 0.000 0.000 0.000 0.000

.T1LIS 0.000 0.000 0.000 0.000 0.000

.T2LIS 0.000 0.000 0.000 0.000 0.000

.T3LIS 0.000 0.000 0.000 0.000 0.000

.RIBV 1.000 1.000 1.000 0.957 0.957

.RIFS 1.000 1.000 1.000 0.930 0.930

.RIIS 1.000 1.000 1.000 0.946 0.946

T1WBV 1.000 1.000 1.000 1.000 1.000

.T2WBV 1.000 1.000 1.000 0.922 0.922

.T3WBV 1.000 1.000 1.000 0.915 0.915

T1WFS 1.000 1.000 1.000 1.000 1.000

.T2WFS 1.000 1.000 1.000 0.939 0.939

.T3WFS 1.000 1.000 1.000 0.913 0.913

T1WIS 1.000 1.000 1.000 1.000 1.000

.T2WIS 1.000 1.000 1.000 0.913 0.913

.T3WIS 1.000 1.000 1.000 0.943 0.943

Constraints:

|Slack|

lambda.1_1 - (3-lambda.2_1-lambda.3_1) 0.000

nu.1.g1 - (0-nu.2.g1-nu.3.g1) 0.000

nu.1.g2 - (0-nu.2.g2-nu.3.g2) 0.000

nu.1.g1 - (0-nu.2.g1-nu.3.g1) 0.000

nu.1.g2 - (0-nu.2.g2-nu.3.g2) 0.000

nu.1.g1 - (0-nu.2.g1-nu.3.g1) 0.000

nu.1.g2 - (0-nu.2.g2-nu.3.g2) 0.000

lambda.10_4 - (2-lambda.11_4) 0.000

nu.10.g1 - (0-nu.11.g1) 0.000

nu.10.g2 - (0-nu.11.g2) 0.000

nu.10.g1 - (0-nu.11.g1) 0.000

nu.10.g2 - (0-nu.11.g2) 0.000

nu.10.g1 - (0-nu.11.g1) 0.000

nu.10.g2 - (0-nu.11.g2) 0.000

lmbd.16_7-(4-lmbd.17_7-lmbd.18_7-lm.19_7) 0.000

nu.16.g1 - (0-nu.17.g1-nu.18.g1-nu.19.g1) 0.000

nu.16.g2 - (0-nu.17.g2-nu.18.g2-nu.19.g2) 0.000

nu.16.g1 - (0-nu.17.g1-nu.18.g1-nu.19.g1) 0.000

nu.16.g2 - (0-nu.17.g2-nu.18.g2-nu.19.g2) 0.000

nu.16.g1 - (0-nu.17.g1-nu.18.g1-nu.19.g1) 0.000

nu.16.g2 - (0-nu.17.g2-nu.18.g2-nu.19.g2) 0.000

beta.13_10.g1 - (beta.16_13.g1) 0.000

beta.13_11.g1 - (beta.16_14.g1) 0.000

beta.13_12.g1 - (beta.16_15.g1) 0.000

beta.14_10.g1 - (beta.17_13.g1) 0.000

psi.13_14.g1 - (psi.16_17.g1) 0.000

psi.13_15.g1 - (psi.16_18.g1) 0.000

beta.13_11.g2 - (beta.16_14.g2) 0.000

beta.13_12.g2 - (beta.16_15.g2) 0.000

# Appendix L) Effects of Covariates on Random Intercepts of Bullying, Friendship and Social Support, and Internalizing Symptoms

**Table S22**

*Effects of Covariates on Random intercepts*

|  | Girls | | | |  | Boys | | | |
| --- | --- | --- | --- | --- | --- | --- | --- | --- | --- |
| *path* | *b* | *p* | *CI* | *beta* |  | *b* | *p* | *CI* | *beta* |
| ETHNICITY → RIBV | -0.39*** | < .001 | [-0.46, -0.32] | -0.38 |  | -0.21*** | < .001 | [-0.28, -0.15] | -0.21 |
| SEN → RIBV | 0.40*** | < .001 | [0.29, 0.52] | 0.39 |  | 0.43*** | < .001 | [0.33, 0.52] | 0.42 |
| FSM → RIBV | 0.23*** | < .001 | [0.16, 0.30] | 0.22 |  | 0.09** | 0.01 | [0.02, 0.17] | 0.09 |
| ETHNICITY → RIFS | 0.15*** | < .001 | [0.08, 0.21] | 0.14 |  | 0.27*** | < .001 | [0.20, 0.33] | 0.26 |
| SEN → RIFS | -0.48*** | < .001 | [-0.60, -0.37] | -0.47 |  | -0.44*** | < .001 | [-0.52, -0.35] | -0.42 |
| FSM → RIFS | -0.31*** | < .001 | [-0.38, -0.23] | -0.3 |  | -0.32*** | < .001 | [-0.39, -0.24] | -0.3 |
| ETHNICITY → RIIS | -0.39*** | < .001 | [-0.45, -0.33] | -0.39 |  | -0.34*** | < .001 | [-0.40, -0.28] | -0.33 |
| SEN → RIIS | 0.18*** | < .001 | [0.09, 0.26] | 0.18 |  | 0.38*** | < .001 | [0.31, 0.46] | 0.37 |
| FSM → RIIS | 0.09** | 0.004 | [0.03, 0.15] | 0.09 |  | 0.12*** | < .001 | [0.06, 0.19] | 0.12 |

*Note.* *Note.* BV = Bullying victimization. FS = Friendship and social support. IS = Internalizing symptoms. RI = Random Intercept. FSM = free school meal eligibility. SEN = special educational needs.

*p < .05, **p <.01, ***p <.001

# Reference

Adachi, P., & Willoughby, T. (2015). Interpreting effect sizes when controlling for stability effects in longitudinal autoregressive models: Implications for psychological science. *European Journal of Developmental Psychology*, *12*(1), 116-128. https://doi.org/10.1080/17405629.2014.963549

Auerswald, M., & Moshagen, M. (2019). How to determine the number of factors to retain in exploratory factor analysis: A comparison of extraction methods under realistic conditions. *Psychological Methods*, *24*(4), 468-491. https://doi.org/10.1037/met0000200

Black, L., Humphrey, N., Panayiotou, M., & Marquez, J. (2024). Mental Health and Well-being Measures for Mean Comparison and Screening in Adolescents: An Assessment of Unidimensionality and Sex and Age Measurement Invariance. *Assessment*, 31(2), 219-236. https://doi.org/10.1177/10731911231158623

Chen, F. F. (2007). Sensitivity of Goodness of Fit Indexes to Lack of Measurement Invariance. *Structural Equation Modeling: A Multidisciplinary Journal*, *14*(3), 464-504. https://doi.org/10.1080/10705510701301834

Cheung, G. W., & Rensvold, R. B. (2002). Evaluating goodness-of-fit indexes for testing measurement invariance. *Structural Equation Modeling*, *9*(2), 233-255. https://doi.org/10.1207/S15328007SEM0902_5

Cohen, J. (1992). Statistical Power Analysis. *Current Directions in Psychological Science*, *1*(3), 98-101. https://doi.org/10.1111/1467-8721.ep10768783

Finney, S. J., & DiStefano, C. (2013). Nonnormal and categorical data in structural equation modeling. In *Structural equation modeling: A second course, 2nd ed.* (pp. 439-492). IAP Information Age Publishing.

Götz, F. M., Gosling, S. D., & Rentfrow, P. J. (2022). Small Effects: The Indispensable Foundation for a Cumulative Psychological Science. *Perspectives on Psychological Science*, *17*(1), 205-215. https://doi.org/10.1177/1745691620984483

Grosz, M. P., Schwartz, S. H., & Lechner, C. M. (2021). The longitudinal interplay between personal values and subjective well-being: A registered report. *European Journal of Personality*, *35*(6), 881-897. https://doi.org/10.1177/08902070211012923

Hall, R. J., Snell, A. F., & Foust, M. S. (1999). Item Parceling Strategies in SEM: Investigating the Subtle Effects of Unmodeled Secondary Constructs. *Organizational Research Methods*, *2*(3), 233-256. https://doi.org/10.1177/109442819923002

Hamaker, E. L. (2023). The within-between dispute in cross-lagged panel research and how to move forward. *Psychological Methods*, No Pagination Specified-No Pagination Specified. https://doi.org/10.1037/met0000600

Hamaker, E. L., Kuiper, R. M., & Grasman, R. P. P. P. (2015). A critique of the cross-lagged panel model. *Psychological Methods*, *20*, 102-116. https://doi.org/10.1037/a0038889

Jorgensen, T. D., Pornprasertmanit, S., Schoemann, A. M., & Rosseel, Y. (2022). semTools: Useful tools for structural equation modeling. https://CRAN.R-project.org/package=semTools

Kline, R. B. (2023). *Principles and Practice of Structural Equation Modeling*. The Guilford Press.

Little, R. J. A. (1988). A Test of Missing Completely at Random for Multivariate Data with Missing Values. *Journal of the American Statistical Association*, *83*(404), 1198-1202. <https://doi.org/10.1080/01621459.1988.10478722>

Little, T. D., Rioux, C., Odejimi, O. A., & Stickley, Z. L. (2022). *Parceling in Structural Equation Modeling: A Comprehensive Introduction for Developmental Scientists.* Cambridge: Cambridge University Press.

Marquez, J., Humphrey, N., Black, L., Cutts, M. & Khanna, D. (2023). Gender identity and sexual orientation inequalities in adolescent wellbeing: Early findings from the #BeeWell study. *BMC Public Health*, 23, 2211-2230.

Mulder, J. D., & Hamaker, E. L. (2021). Three extensions of the random intercept cross-lagged panel model. *Structural Equation Modeling*, *28*(4), 638-648. https://doi.org/10.1080/10705511.2020.1784738

Orth, U., Clark, D. A., Donnellan, M. B., & Robins, R. W. (2021). Testing prospective effects in longitudinal research: Comparing seven competing cross-lagged models. *Journal of Personality and Social Psychology*, *120*, 1013-1034. https://doi.org/10.1037/pspp0000358

Orth, U., Meier, L. L., Bühler, J. L., Dapp, L. C., Krauss, S., Messerli, D., & Robins, R. W. (2022). Effect size guidelines for cross-lagged effects. *Psychological Methods*. https://doi.org/10.1037/met0000499

Rhemtulla, M., Brosseau-Liard, P. É., & Savalei, V. (2012). When can categorical variables be treated as continuous? A comparison of robust continuous and categorical SEM estimation methods under suboptimal conditions. *Psychological Methods*, *17*(3), 354-373. https://doi.org/10.1037/a0029315

Robitzsch, A. (2020). Why Ordinal Variables Can (Almost) Always Be Treated as Continuous Variables: Clarifying Assumptions of Robust Continuous and Ordinal Factor Analysis Estimation Methods [Perspective]. *Frontiers in Education*, *5*. https://doi.org/10.3389/feduc.2020.589965

Rosseel, Y. (2012). lavaan: An R Package for Structural Equation Modeling. *Journal of Statistical Software*, *48*(2), 1 - 36. https://doi.org/10.18637/jss.v048.i02

Rubin, M. (2021). When to adjust alpha during multiple testing: a consideration of disjunction, conjunction, and individual testing. *Synthese*, *199*(3), 10969-11000. https://doi.org/10.1007/s11229-021-03276-4

Schermelleh-Engel, K., Moosbrugger, H., & Müller, H. (2003). Evaluating the Fit of Structural Equation Models: Tests of Significance and Descriptive Goodness-of-Fit Measures. *Methods of Psychological Research*, *8*(2), 23-74.

Usami, S., Todo, N., & Murayama, K. (2019). Modeling reciprocal effects in medical research: Critical discussion on the current practices and potential alternative models. *PloS one*, *14*(9), e0209133. https://doi.org/10.1371/journal.pone.0209133
